# Identification of an air- and moisture-tolerant MOF-based C–H amination catalyst

**DOI:** 10.1039/d6cc02735k

**Published:** 2026-06-09

**Authors:** John C. Izang, James R. Bour

**Affiliations:** a Wayne State University Department of Chemistry 5101 Cass Avenue Detroit MI 48202 USA bour@wayne.edu

## Abstract

PCN-222(Co), a mesoporous Zr-based cobalt–porphyrin MOF, catalyses benzylic C–H amination under air, with added water, and with limiting C–H substrate.

## Abstract 2

A MOF-based C–H amination catalyst that operates efficiently in the presence of air and moisture is described.

## Graphical abstract

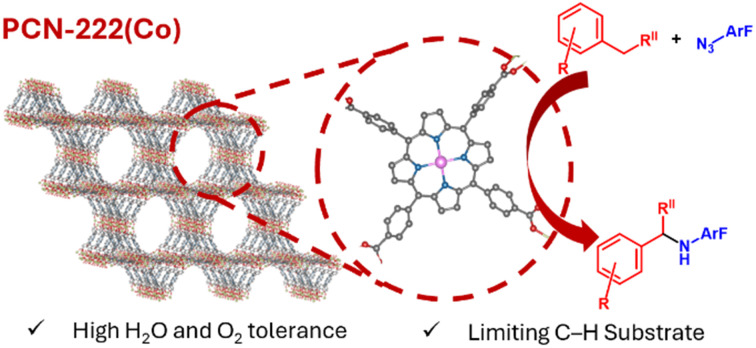

Direct conversion of C–H bonds into C–N bonds is a longstanding objective in the synthesis of organic amines because it bypasses prefunctionalized substrates to access valuable amine products.^1–4^ Among the available strategies, nitrene transfer from organic azides is especially attractive, and Co^II^ metalloradical porphyrins are particularly effective catalysts for this transformation.^5–17^ They combine low cost with high activity and excellent selectivity across a wide range of weak C–H bonds. The development of these catalysts represents a significant advance but a practical limitation remains: most high-performing systems generally require rigorously inert, anhydrous conditions. At least three deactivation pathways under such conditions have been invoked, including bimolecular decomposition of Co^III^–nitrene radical intermediates, oxidation by O_2_, and/or axial coordination of H_2_O at cobalt.^18–21^

Porphyrin metal–organic frameworks provide an attractive platform for stabilizing reactive intermediates and directing substrate–catalyst interactions to help overcome deactivation limitations.^22,23^ Incorporating the porphyrin as a structural linker limits bimolecular catalyst interactions without the typical steric bulk required to avoid similar interactions in molecular analogues. In addition, the polarity, substrate partitioning, and effective water activity within MOF pores can differ substantially from the bulk medium.^24,25^ Taken together, these effects and others often augment the activity and stability of catalytic centers confined in MOFs relative to their solution counterparts.^26–29^ Consistent with these observations, heterogenized metalloporphyrins have shown enhanced robustness in several catalytic contexts,^30,31^ and porphyrinic MOFs have been widely applied in oxidation, CO_2_ reduction, and photocatalysis.^32–35^ Their use in nitrene-transfer catalysis, however, remains comparatively limited. More importantly, to our knowledge, no study has directly tested whether embedding a cobalt porphyrin nitrene-transfer catalyst within a porous framework can preserve catalytic activity under the air- and moisture-exposed conditions that deactivate the corresponding molecular analogue. Recent studies showing that confinement of metalloradical porphyrins within MOFs can strongly influence active-site reactivity further support this hypothesis.^36,37^

Translating this hypothesis into a practical catalyst platform is not simple. The performance of nitrene-transfer C–H amination catalysts depends strongly on active site microenvironment, azide identity, solvent, and temperature, making it difficult to predict a productive MOF system a priori.^38^ High-throughput screening offers an attractive way to identify active catalysts and conditions,^39–41^ but applying such workflows to MOF catalysis remains nontrivial. MOFs are insoluble, crystalline powders, which are often mechanically unstable.^42^ Reliable and non-destructive dosing of sub-milligram quantities without advanced robotics is still a practical barrier to parallel reaction screens. As such, evaluation of MOF catalysts is still often limited to labour-intensive, manually weighed experiments.^39,40^

Here, we report the discovery, optimization, and evaluation of a MOF-based C–H amination catalyst that is highly tolerant of water and oxygen. Using a solution dispersion reactor-dosing workflow to screen reaction conditions (Fig. 1a), we identified PCN-222(Co), a mesoporous Zr-based framework containing a cobalt–porphyrin linker, as an effective catalyst for benzylic amination. Under inert, anhydrous conditions, PCN-222(Co) performs similarly to closely related molecular cobalt porphyrins. Under aerobic and moist conditions, however, its behaviour diverges sharply. Whereas the molecular catalysts are largely shut down by O_2_ and H_2_O, PCN-222(Co) retains high activity. The catalyst also remains effective under limiting C–H substrate conditions and after prolonged benchtop storage.

**Fig. 1:**
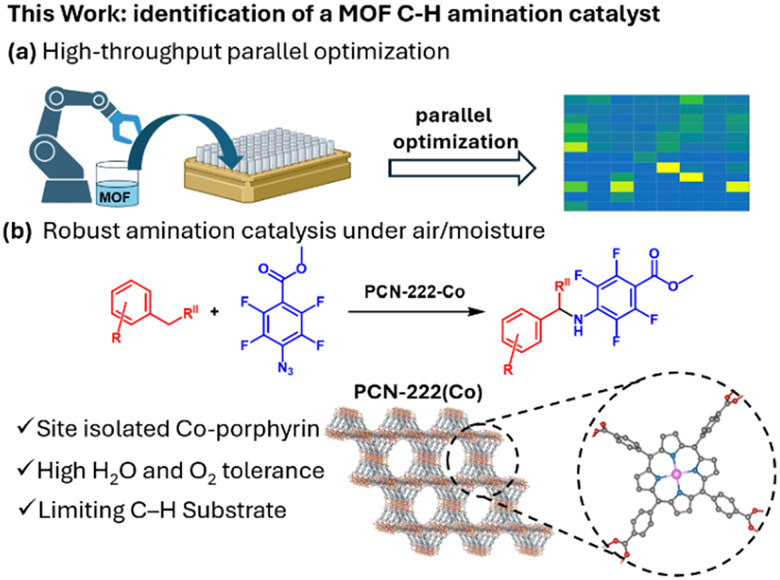
General overview of this work (a) Solution dispersion reactor dosing for high-throughput MOF catalyst assays used in this work and (b) general reaction scheme for the amination reaction in this communication, with a zoomed-out view of the porphyrin linker in PCN-222(Co).

Catalyst selection and dosing strategy. We selected PCN-222(Co) as the initial candidate for three reasons. First, PCN-222 possesses an isolated porphyrin within a mesoporous (3.7 nm) channel (Fig. 1b), which should reduce diffusional penalties that can limit catalysis in primarily microporous catalysts.^43,44^ Second, PCN-222 is thermally robust and generally stable to ambient moisture, tolerating aqueous and acidic media.^43,45^ Third, the tetra-carboxyphenyl porphyrin (TCPP) linker features a broader ligand environment similar to cobalt tetraphenyl porphyrin (CoTPP)-based catalysts that are widely reported to be effective for C–H amination.

We initially pursued high-throughput evaluation of PCN-222(Co) using our previously reported MOFBead protocol. In this protocol, pulverized MOF is dispersed in glass microbeads and dosed volumetrically to reactors using calibrated scoops.^39^ Application of this method to PCN-222(Co), however, produced significant losses in BET surface area (1642 to 549 m^2^ g^−1^) despite maintaining qualitatively similar PXRD patterns (Fig. S15, S17 and S18). PCN-222(Co) is apparently insufficiently stable to the mechanical grinding required for this approach. We therefore sought to apply a milder solution dispersion-based dosing protocol similar to that previously reported by Cohen.^40^

To test the feasibility of solution dispersion dosing techniques, PCN-222(Co) was suspended in THF, sonicated, then pipetted into reactor wells using an automated liquid handling robot. Gas adsorption isotherms and PXRD patterns of solution dispersed MOF confirm that this approach results in less structural degradation than solid dispersion strategies. Fig. 2b shows that the surface area was minimally affected by solution-dispersion dosing, as determined by N_2_ adsorption isotherms at 77 K (1642 to 1606 m^2^ g^−1^). Crystallinity is also maintained, as indicated by PXRD (Fig. S15).

**Fig. 2:**
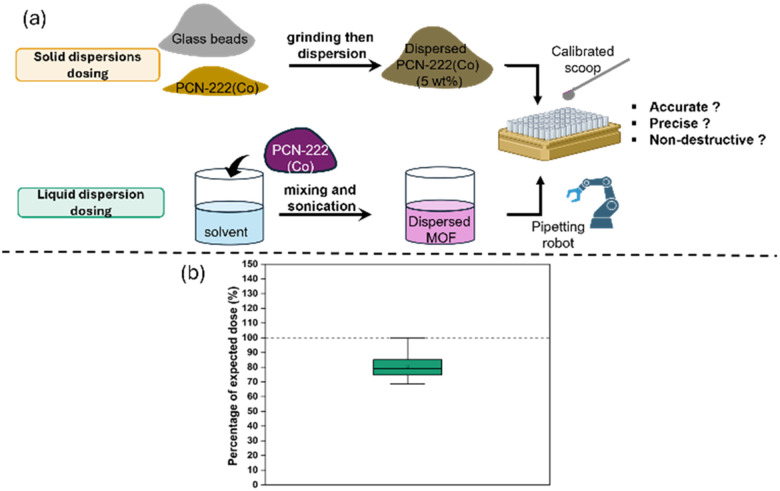
(a) Overview of the two reactor dosing approaches evaluated in this work. (b) Boxplot showing the accuracy and precision of the liquid dispersion loading of PCN-222(Co) into reactor wells a 250 µg per well.

Having demonstrated that the MOF maintains its structure using this approach, we next sought to characterize the well-to-well dosing consistency. PCN-222(Co) MOF was dosed into 24 reactor wells, digested in basic DMSO, and the absorbance of each well was measured by plate reader against a calibration curve.^39,46^ As shown in Fig. 2b, dosing precision and accuracy were good at just 250 µg of MOF per well. This method gave a well-to-well dosing coefficient of variation of 10.3%, with an average of 80% of the expected MOF delivered to each well (Fig. 2(b)), which is typical for reactor dosing precision in high-throughput chemistry workflows.^47–51^

Reaction optimization. With a non-destructive reactor dosing strategy in hand, we next carried out a three-dimensional C–H amination activity screen (Fig. 3). Four representative azides (Troc-N_3_,^52^ Tosyl-N_3_,^53^ Nosyl-N_3_,^54^ and Fluoro-N_3_^55^), six solvents (dichloromethane [DCM], MeCN, 1,4-dioxane, fluorobenzene, hexafluoroisopropanol [HFIP], and 1,2-difluorobenzene [DFB]), and four temperatures (23, 50, 80, and 120 °C), using ethylbenzene (10 equiv.) as the C–H partner with 5 mol% PCN-222(Co) were evaluated. Approximate yields were determined by GC analysis and higher-yielding conditions were confirmed by NMR spectroscopy against an internal standard. As shown in Fig. 3, methyl 4-azido-tetrafluorobenzoate emerged as a uniquely productive azide across all temperatures evaluated. The optimal yield was observed in PhF at 50 °C, giving a ^19^F NMR yield of 67%. The other azides, Troc-, Tosyl-, and Nosyl-N_3_, afforded almost no product until 120 °C. Solvent effects were broadly consistent with trends reported for related transformations.^5–17^ Aromatic solvents (PhF, DFB) outperformed other aprotic media. The temperature dependence was weak between 50 and 120 °C (67%, 64%, and 63% by ^19^F NMR).

**Fig. 3:**
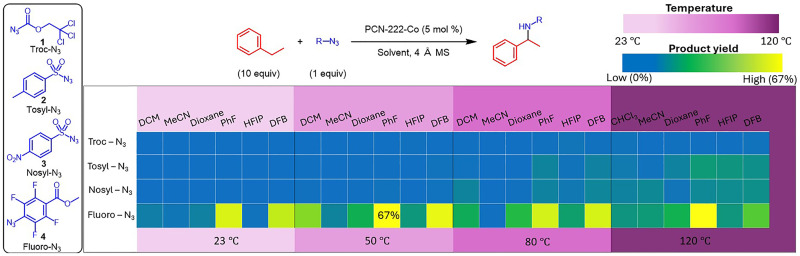
Initial azide, solvent and temperature screen to determine activity. Yield is as determined by GC-MS with high yielding conditions confirmed by quantitative NMR. DCM = dichloromethane, MeCN = acetonitrile, dioxane = 1,4-dioxane, PhF = fluorobenzene, DFB = 1,2-difluorobenzene, MS = molecular sieves. All reactions were conducted under an N_2_ atmosphere.

Air- and moisture-sensitivity studies. To test if catalyst confinement in the MOF facilitated air and moisture tolerance, we next evaluated the activity of PCN-222(Co) relative to close molecular analogues, Co^II^ tetraphenylporphyrin [Co(TPP)] and Co^II^ tetramethyl carboxyphenyl porphyrin [Co(TMCPP)]. Under N_2_, PCN-222(Co), Co(TPP), and Co(TMCPP) catalyze the benzylic amination of ethylbenzene with Fluoro-N_3_ at broadly comparable yields (88%, 79%, and 55%, respectively, Fig. 4a). However, the activities of the three catalysts under air diverge sharply (Fig. 4b). Co(TPP) and Co(TMCPP) both deliver <5% yield, consistent with the established sensitivity of molecular Co–porphyrin amination catalysts to air. The PCN-222(Co) catalyst, in contrast, retains 74% ^19^F NMR yield under the same open-flask conditions, a loss of only ∼14% yield relative to its inert-atmosphere performance. Because Co(TMCPP) bears a similar electronic environment to the framework linker, the divergence between Co(TMCPP) and PCN-222(Co) is consistent with a protective framework microenvironment, rather than a simple electronic effect.

**Fig. 4:**
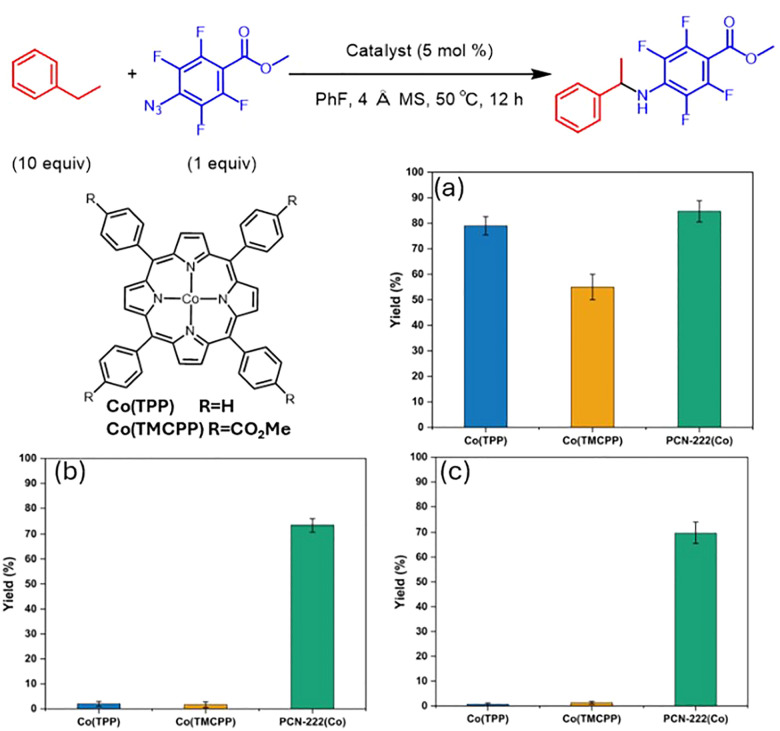
Evaluation of PCN-222(Co) in C–H amination reactions under inert and aerobic environments. Yields are averages of three trials. (a) Comparison to representative molecular catalysts under N_2_ atmospheres. (b) Comparison to representative molecular catalysts under ambient atmosphere. (c) Comparison of the catalysts with 3% v/v added water.

The robustness of the MOF catalyst to water was also investigated. Introduction of 3% v/v H_2_O shuts down both molecular catalysts entirely, whereas PCN-222(Co) delivers 69% yield under the same conditions (Fig. 4c). Increasing water content incrementally to 100% v/v produces a gradual attenuation of yield but still delivers 58% of the aminated product with water as the only added solvent (Fig. S12). We are cautious about interpreting the high-bulk-water results as a true measure of aqueous catalysis as these reactions were conducted with ten equivalents of ethylbenzene relative to azide. Both reaction partners are likely to partition into the hydrophobic PCN-222 pore, thereby reducing water activity at the active site. Nonetheless, these results demonstrate that the catalyst is overall quite robust to air and water. Consistent with this stability, the catalyst maintained its activity for 10 weeks when stored on the bench (Fig. S13 and S14).

Substrate scope. We evaluated the generality of PCN-222(Co)-catalysed benzylic amination across a panel of substrates with benzylic C–H bonds under aerobic conditions (Fig. 5), reporting each yield under two stoichiometries: condition A, with the C–H substrate limiting (1.0 equiv.) against Fluoro-N_3_ (2.0 equiv.), relevant to late-stage functionalization of a valuable substrate; and condition B, with the C–H substrate used as 10 equivalents relative to the azide, which generally gives higher yields. Under both conditions, molecular sieves were used because they boosted yields 5–10% relative to their absence. As shown in Fig. 5, the catalyst is tolerant of electron rich, neutral and poor substitution patterns. Electron-poor substrates (9–13) performed well with both conditions A and B, yielding moderate to good yields of isolated product. Electron-neutral to slightly rich substrates (14–23) afforded product in good yields as well. Carbocycles with multiple accessible benzylic C–H bonds such as tetrahydronaphthalene and indenes were tolerated under both conditions A and B but with a larger yield gap between A and B than other substrates, potentially due to difunctionalization at the other accessible C–H site. The reaction did not, however, tolerate pyridines or phenolic alcohols (30 and 31).

**Fig. 5:**
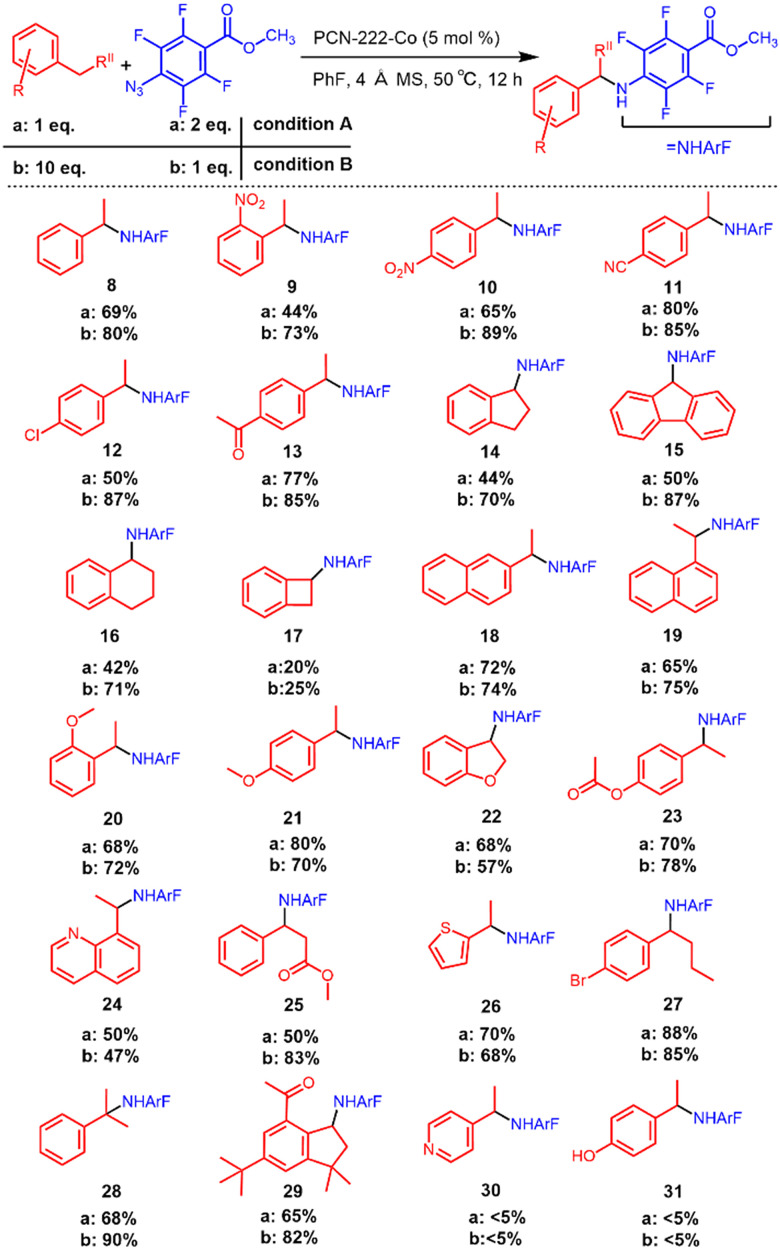
Substrate scope of PCN-222(Co) catalysed reaction under air. Reaction conditions: Fluoro-N_3_ (0.12 mmol for condition A, and 0.06 for condition B), benzylic substrate (0.06 mmol for condition A, and 0.6 mmol for condition B), PCN-222(Co) (0.003 mmol of Co, 5 mol%), PhF (0.3 mL), molecular sieves (15 mg). All reported yields are isolated yields.

A notable feature of the MOF-based catalyst is its effectiveness under limiting C–H conditions (condition A). Although condition B delivers higher yields for most substrates, particularly those with multiple accessible benzylic C–H bonds, yields under condition A remain synthetically useful. Practically, this allows the choice between A and B to be determined by which reagent is more valuable, not by catalyst activity limitations. Catalysts that deliver high-yielding C–H amination under limiting C–H conditions remain rare, and in this case the tolerance appears to originate, at least in part, from confinement in the framework. Repeating condition A under N_2_ with molecular Co(TPP) gave only 29% yield by ^19^F NMR, compared with 74% for PCN-222(Co).

In conclusion, this work demonstrates that embedding a Co^II^ porphyrin as the structural linker of the mesoporous MOF PCN-222(Co) confers air and moisture tolerance on a reaction class that is largely restricted to rigorously inert, anhydrous conditions using molecular catalysts. Direct comparison with the electronically similar molecular analogue Co(TMCPP), as well as Co(TPP), argues against a simple linker-electronic origin for this robustness and is instead consistent with a protective framework microenvironment. Although further mechanistic work is needed, we propose that the MOF may limit bimolecular interactions in a manner analogous to bulky peripheral substituents, but without introducing steric crowding at the active site.^56^ PCN-222(Co) shows good activity under C–H substrate-limiting conditions and remains active after prolonged bench storage, enhancing its synthetic utility. Together, the stability and activity of the present system suggest that MOFs merit broader consideration as accessible platforms for fine chemical transformations.

## Conflicts of interest

There are no conflicts to declare.

## Supplementary Material

CC-062-D6CC02735K-s001

## Data Availability

The data supporting this article have been included as part of the supplementary information (SI). Supplementary information: supplemental data, experimental details, and characterization data of all new compounds. See DOI: https://doi.org/10.1039/d6cc02735k.

## References

[cit1] Park Y., Kim Y., Chang S. (2017). Chem. Rev..

[cit2] Tsang W. C. P., Munday R. H., Brasche G., Zheng N., Buchwald S. L. (2008). J. Org. Chem..

[cit3] Tsang W. C. P., Zheng N., Buchwald S. L. (2005). J. Am. Chem. Soc..

[cit4] Morofuji T., Shimizu A., Yoshida J. (2014). J. Am. Chem. Soc..

[cit5] Lu H., Subbarayan V., Tao J., Zhang X. P. (2010). Organometallics.

[cit6] Lu H., Jiang H., Hu Y., Wojtas L., Zhang X. P. (2011). Chem. Sci..

[cit7] Ragaini F., Penoni A., Gallo E., Tollari S., Li Gotti C., Lapadula M., Mangioni E., Cenini S. (2003). Chem. - Eur. J..

[cit8] Cenini S., Tollari S., Penoni A., Cereda C. (1999). J. Mol. Catal. A: Chem..

[cit9] Lyaskovskyy V., Suarez A. I. O., Lu H., Jiang H., Zhang X. P., De Bruin B. (2011). J. Am. Chem. Soc..

[cit10] Zardi P., Intrieri D., Caselli A., Gallo E. (2012). J. Organomet. Chem..

[cit11] Goswami M., Lyaskovskyy V., Domingos S. R., Buma W. J., Woutersen S., Troeppner O., Ivanović-Burmazović I., Lu H., Cui X., Zhang X. P., Reijerse E. J., DeBeer S., van Schooneveld M. M., Pfaff F. F., Ray K., de Bruin B. (2015). J. Am. Chem. Soc..

[cit12] Zhu Y., Lee W.-C. C., Zhang X. P. (2025). J. Am. Chem. Soc..

[cit13] Lang K., Hu Y., Lee W.-C. C., Zhang X. P. (2022). Nat. Synth..

[cit14] Lee W.-C. C., Zhang X. P. (2024). Angew. Chem., Int. Ed..

[cit15] Lang K., Torker S., Wojtas L., Zhang X. P. (2019). J. Am. Chem. Soc..

[cit16] Caselli A., Gallo E., Fantauzzi S., Morlacchi S., Ragaini F., Cenini S. (2008). Eur. J. Inorg. Chem..

[cit17] Kuijpers P. F., Tiekink M. J., Breukelaar W. B., Broere D. L. J., van Leest N. P., van der Vlugt J. I., Reek J. N. H., de Bruin B. (2017). Chem. - Eur. J..

[cit18] Walker F. A. (1973). J. Am. Chem. Soc..

[cit19] Marianov A. N., Kochubei A. S., Roman T., Conquest O. J., Stampfl C., Jiang Y. (2021). ACS Catal..

[cit20] Nakazono T., Parent A. R., Sakai K. (2013). Chem. Commun..

[cit21] Goswami M., Rebreyend C., De Bruin B. (2016). Molecules.

[cit22] Zhang W., Nafady A., Shan C., Wojtas L., Chen Y.-S., Cheng Q., Zhang X. P., Ma S. (2021). Angew. Chem., Int. Ed..

[cit23] Wang X.-S., Chrzanowski M., Kim C., Gao W.-Y., Wojtas L., Chen Y.-S., Zhang X. P., Ma S. (2012). Chem. Commun..

[cit24] Xie L.-H., Xu M.-M., Liu X.-M., Zhao M.-J., Li J.-R. (2020). Adv. Sci..

[cit25] Li L., Yang Q., Chen S., Hou X., Liu B., Lu J., Jiang H.-L. (2017). Chem. Commun..

[cit26] Reiner B. R., Kassie A. A., Wade C. R. (2019). Dalton Trans..

[cit27] Burgess S. A., Kassie A., Baranowski S. A., Fritzsching K. J., Schmidt-Rohr K., Brown C. M., Wade C. R. (2016). J. Am. Chem. Soc..

[cit28] Piradi V., Chai W., Emslie S. K., Sikma R. E., Zhang C., Vasylevskyi S., Henkelman G., Humphrey S. M. (2025). J. Am. Chem. Soc..

[cit29] Dunning S. G., Nandra G., Conn A. D., Chai W., Sikma R. E., Lee J. S., Kunal P., Reynolds III J. E., Chang J., Steiner A., Henkelman G., Humphrey S. M. (2018). Angew. Chem., Int. Ed..

[cit30] Pereira M. M., Dias L. D., Calvete M. J. F. (2018). ACS Catal..

[cit31] Fukuzumi S., Mochizuki S., Tanaka T. (1987). Isr. J. Chem..

[cit32] Jin J. (2020). New J. Chem..

[cit33] Gao W.-Y., Chrzanowski M., Ma S. (2014). Chem. Soc. Rev..

[cit34] Fateeva A., Chater P. A., Ireland C. P., Tahir A. A., Khimyak Y. Z., Wiper P. V., Darwent J. R., Rosseinsky M. J. (2012). Angew. Chem., Int. Ed..

[cit35] Zou L., Sa R., Lv H., Zhong H., Wang R. (2020). ChemSusChem.

[cit36] Qiu Z., Bruzzese P. C., Wang Z., Deng H., Leutzsch M., Farès C., Chabbra S., Neese F., Schnegg A., Neumann C. N. (2025). J. Am. Chem. Soc..

[cit37] Qiu Z., Deng H., Neumann C. N. (2024). Angew. Chem., Int. Ed..

[cit38] Khatua H., Ghosh A., Das S., Patra S., Nandi S., Chattopadhyay B. (2026). Chem. Rev..

[cit39] Li S., Izang J. C., Duque J. B., Leonard G. D., Bour J. R. (2026). Inorg. Chem..

[cit40] Palomba J. M., Harvey S. P., Kalaj M., Pimentel B. R., DeCoste J. B., Peterson G. W., Cohen S. M. (2020). ACS Appl. Mater. Interfaces.

[cit41] McCullough K. E., King D. S., Chheda S. P., Ferrandon M. S., Goetjen T. A., Syed Z. H., Graham T. R., Washton N. M., Farha O. K., Gagliardi L., Delferro M. (2023). ACS Cent. Sci..

[cit42] Redfern L. R., Farha O. K. (2019). Chem. Sci..

[cit43] Feng D., Gu Z.-Y., Li J.-R., Jiang H.-L., Wei Z., Zhou H.-C. (2012). Angew. Chem., Int. Ed..

[cit44] Morris W., Volosskiy B., Demir S., Gándara F., McGrier P. L., Furukawa H., Cascio D., Stoddart J. F., Yaghi O. M. (2012). Inorg. Chem..

[cit45] Lv D., Shi R., Chen Y., Chen Y., Wu H., Zhou X., Xi H., Li Z., Xia Q. (2018). Ind. Eng. Chem. Res..

[cit46] Wang A., Barcus K., Cohen S. M. (2023). J. Am. Chem. Soc..

[cit47] Impastato A. C., Brown J. T. C., Wang Y., Tu N. P. (2023). ACS Med. Chem. Lett..

[cit48] Tu N. P., Wang Y. (2026). RSC Med. Chem..

[cit49] Gesmundo N. J., Tu N. P., Sarris K. A., Wang Y. (2023). ACS Med. Chem. Lett..

[cit50] Meng J., Zhou C. H., Yin L., Chen J.-A., Xi J., Chen C., Cai T., Gui Y. (2025). Mol. Pharmaceutics.

[cit51] Tu N. P., Dombrowski A. W., Goshu G. M., Vasudevan A., Djuric S. W., Wang Y. (2019). Angew. Chem., Int. Ed..

[cit52] Lu H., Subbarayan V., Tao J., Zhang X. P. (2010). Organometallics.

[cit53] Suarez A. I. O., Jiang H., Zhang X. P., de Bruin B. (2011). Dalton Trans..

[cit54] Lyaskovskyy V., Suarez A. I. O., Lu H., Jiang H., Zhang X. P., de Bruin B. (2011). J. Am. Chem. Soc..

[cit55] Jin L.-M., Xu X., Lu H., Cui X., Wojtas L., Zhang X. P. (2013). Angew. Chem., Int. Ed..

[cit56] Kuijpers P. F., Tiekink M. J., Breukelaar W. B., Broere D. L. J., van Leest N. P., van der Vlugt J. I., Reek J. N. H., de Bruin B. (2017). Chem. - Eur. J..

