# Supplementary material for: Identification of an air- and moisture-tolerant MOF-based C–H amination catalyst
Source: Chem Commun (Camb). 2026 Jun 9;62(53):13383–7. doi: 10.1039/d6cc02735k (PMC13284688; doi:10.1039/d6cc02735k)

Supplementary Information (SI) for ChemComm.
This journal is © The Royal Society of Chemistry 2026

Supporting information for

Identification of an Air- and Moisture-Tolerant MOF-Based C–H Amination Catalyst

John C. Izang, and James R. Bour*

Department of Chemistry
Wayne State University
5101 Cass Ave. Detroit, MI 48202
E-mail: bour@wayne.edu

TABLE OF CONTENTS

1. Materials and Methods S4
1.1. Chemicals S3
1.2. Analytical Techniques and Key Equipment S3
2. Syntheses of MOFs, Azides and Authentic Standards S5
2.1. Synthesis of Catalysts S5
2.2. Synthesis of Organic azides S6
2.3. Synthesis of Aminated Product S8
3.0 HTE workflow Development S10
3.1 HTE Solid Reactor Dosing (MOFBead) S10
3.2 HTE Solution Dispersion Reactor Dosing S10
3.3 Procedure for Parallel Optimizations S12
4.0 Catalysis S13
4.1 Reaction Screen S13
4.1.1 High-throughput Reaction (HTE) Screen S13
4.1.2 GC-MS Analysis S15
4.1.3 Aminated Products Quantitation S15
4.2 Reaction Optimization and Robustness S17
4.3 Substrate Scope S24
4.3.1 General Procedures A and B S24
5.0 Catalyst Characterization S36
5.1 PXRD Characterization S36
5.2 N_2 Adsorption Isotherms S37
5.3 SEM-EDS S38
5.4 Post-Catalysis Catalyst Characterization S39
6.0 Filtration and Recycling Experiments S41
7.0 References S43
8.0 NMR spectra of all compounds S45

1. Materials and Methods

1.1 Chemicals

Abbreviations used for chemicals: DMF (dimethylformamide), MeOH (methanol), DMSO (dimethyl sulfoxide), EtOH (ethanol), Et_2O (diethyl ether), DCM (dichloromethane), MeCN (acetonitrile), THF (tetrahydrofuran), DMA (dimethylacetamide), EtOAc (ethyl acetate), HFIP (hexafluoroisopropanol), PhF (fluorobenzene), DFB (1,2-difluorobenzene), Dioxane (1,4-dioxane), TEA (triethylamine), Co(TMCPP) meso-tetra(4-carboxyphenyl)porphin tetramethyl ester cobalt (II), Co(TPP) (5,10,15,20-tetraphenyl-21h,23h-porphine cobalt(II)).

Reagent sourcing and preparation: 5,10,15,20-tetraphenyl-21h,23h-porphine cobalt(ii), meso-tetra(4-carboxyphenyl)porphin tetramethyl ester, 4-nitrobenzenesulfonyl chloride, zirconyl chloride octahydrate, 2-ethylnitrobenzene, 4'-ethylacetophenone, 1,2,3,4-tetrahydronaphthalene,1-phenylethylamine, and methyl 4-ethylbenzoate, were purchased from Combi Blocks. Molecular sieves 4Å powder (activated, - 325 mesh), fluorobenzene, 2-fluorotoluene, 3-ethylpyridine, 1,1,1,3,3,3-hexafluoro-2-propanol, ethylbenzene, 4-ethylnitrobenzene, and 2,2,2-trichloroethyl chloroformate were purchased from Oakwood chemicals**.** 4,4-difluoro-1,1-biphenyl, methyl pentafluorobenzoate, 1-ethyl-4-methoxybenzene, 1-chloro-4-ethylbenzene, 1-ethyl-2-methoxybenzene, 2,3-dihydrobenzofuran, 4-ethylbenzonitrile, trimethyl benzene-1,3,5-tricarboxylate, 1-bromo-4-butylbenzene, 1-ethylnaphthalene, 2-ethylnaphthalene, 8-ethylquinoline, bicyclo[4.2.0]octa-1,3,5-triene, 9h-fluorene, 2-ethylthiophene, methyl 3-phenylpropanoate, and 4-ethylphenyl acetate were purchased from Ambeed. Cumene was purchased from TCI chemicals. Benzoic acid was purchased from Sigma aldrich. Aluminum-backed 200 µm particle size thin layer chromatography (TLC) plates were purchased from Sorbtech. CoClTPP was prepared via literature procedure.[1] ACS-grade solvents were purchased and used as received. All solvents used under inert atmospheres were deoxygenated via three cycles of freeze-pump-thaw degassing followed by backfilling with N_2. All reagents were used as received without further purification.

1.2 Analytical Techniques and Key Equipment:

<u>Vacuum Oven</u>

MOFs activated in parallel in a vacuum oven were activated in a Fisher Brand vacuum oven (~1 torr minimum pressure).

<u>Chromatography and purification</u>

Compounds were purified by flash chromatography using a Combi Flash *NEXTGEN* 300 with pre-column cartridges, RediSep Silver Silica Gel Disposable Flash Columns (24 gram; 40-60 µm particle size; 230-400 mesh size) purchased from Teledyne ISCO.

<u>Reactor Blocks</u>

High-throughput reaction screens were conducted using Para-dox aluminum reactor blocks purchased from Analytical Sales and Solutions in three sizes: 24-well (4 mL inserts), 48-well (2 mL inserts) and 96-well (1 mL inserts).

Nuclear Magnetic Resonance

Nuclear Magnetic Spectra (NMR) spectra were obtained on an Agilent MR-400 MHz (400 MHz for ^{1}H and 101 MHz for ^{13}C), a Bruker 500-MHz (500 MHz for ^{1}H, 126 MHz for ^{13}C and 471 MHz for ^{19}F) and an Agilent DD2-600 MHz (600 MHz for ^{1}H and 565.2MHz for ^{19}F) spectrometer. ^{1}H NMR chemical shifts are reported in parts per million (ppm) relative to tetramethylsilane (TMS), with sample residual solvent peak used as an internal reference ($CHCl_3$ = 7.26 ppm, $(CH_{3-n}D_n)_2CO$ = 2.05 ppm, $(CH_{3-n}D_n)_2SO$ = 2.5 ppm). ^{13}C NMR chemical shifts are reported in parts per million (ppm) relative to tetramethylsilane (TMS), with sample residual solvent peak used as an internal reference ($CDCl_3$ = 77.00 ppm). ^{19}F NMR were referenced to fluorobenzene (-113.15ppm). Quantitative ^{19}F NMR were acquired with a relaxation delay of 15s per scan. The following abbreviations are used to report NMR multiplicity s = singlet, bs = broad singlet, d = doublet, t = triplet, q = quartet, p= quintet, h=sextet, sept=septet, dd = doublet of doublets, ddd = doublet of doublet of doublets, td = triplet of doublets, m = multiplet, dm = doublet of multiplet. *Note that for many fluoroaryl compounds, C–F couplings beyond one bond could not be resolved. As such, they are reported as doublets of multiplets.*

Gas adsorption isotherms

Gas adsorption isotherm of activated samples were measured using Micromeritics 3Flex instrument. Sample activation involves heating samples in a pre-weighted glass analysis tube capped with TranSeal or CheckSeal at 150 °C on a Micromeritics VacPrep 061 (~$3x10^{-2}$ mmHg) for 2-4 hours under vacuum before analysis. Nitrogen adsorption isotherms were obtained using ultra-high purity nitrogen and a 77 K liquid-N_2 bath. Consistency criteria, as described by Rouquerol, were referenced for all samples. [2]

Powder X-ray Diffraction (PXRD)

PXRD Measurements were carried out at room temperature with 30 kV/10 mA radiation. 0.2 mm divergence slit, and 3 mm air scatter screen, using a Bruker Phaser II model X-ray diffractometer with a Cu anode and a Lynxeye detector. 0.5 s/step scanning speed, 2° to 30° range (2θ) with 0.02 increment and 1° PSD opening was used.

High-resolution mass spectrometry (HRMS)

Compound identities were confirmed using High-Resolution Mass Spectrometry with a Thermo Orbitrap Exploris 120 Electrospray Ionization (ESI) in negative mode.

Gas chromatography-mass spectrometry (GC-MS)

Gas chromatography-mass spectrometry (GC-MS) data were collected using a Shimadzu GCMS-QP2010 SE.

OT2 liquid handling robot

OT2 liquid handling robot equipped with second generations p20 and p1000 pipettes were used for pipetting MOFs and other solutions during reaction set-up and work-up. The Opentrons protocol designer was used for protocol developments.

Ultrasonication

Ultrasonication of all samples as described in this paper was conducted using a CPX5800 ultrasonic bath (40 kHz, 9.5 L bath)

2. Syntheses of MOFs, Azides and Authentic Standards

2.1 Synthesis of catalysts

Synthesis of PCN-222

PCN-222 was synthesized by slight modification of the literature.[3] Two stock solutions were prepared. Stock solution A was made by charging a 250 mL pyrex autoclave jar with 600 mg (1.86 mmol) $ZrOCl_2.8H_2O$, benzoic acid (9.00 g, 73.7 mmol) and 20 mL of DMF. Stock solution B was made by charging a 250 mL autoclave jar with Tetracarboxyphenyl porphyrin (TCPP, 300 mg (0.38 mmol) and 20 mL DMF. The solutions were sonicated in their respective jars for 40 min before being placed in a pre-heated oven at 100 °C for 1 h. After 1 h the two solutions were removed from the oven and allowed to cool at room temperature for 15 min. Solution A is then added into solution B. Then 2 mL of TFA was added to the combined solutions of A and B. The solutions were swirled 10 s to mix the TFA after addition. The solution is then immersed in an oil bath at 120 °C for 10 h. The resulting precipitate is collected by centrifugation (20 mins at 4500 rpm, Relative Centrifugal Factor (RCF) = 3,169 g). After centrifugation, the supernatant was carefully decanted, discarded, and replaced by 50 mL of fresh DMF. This washing process was repeated two additional times. After the third DMF wash, the MOF was transferred back into a 250 mL Pyrex autoclave jar before suspension in 100 mL of fresh DMF. To this suspension was added 3 mL of 8 M HCl. The jar was then kept in a pre-heated oven at 120 °C for 16 h. After 16 h, the PCN-222 MOF precipitate is collected after cooling by centrifugation. The precipitate was carefully decanted, discarded, and replaced by 50 mL of fresh DMF. This process was repeated three times with DMF followed by three times with acetone. After the third acetone wash, 50 mL of fresh acetone was added and allowed to stand for 14 h. After the final wash, the resultant purple colored PCN-222 MOF was collected by centrifugation (20 min, 4500 rpm, RCF = 3,162 g) then activated under vacuum (120 °C , 0.5 Torr). Figure (S1a)

Synthesis of PCN-222(Co)

The porphyrin linker of PCN-222 was metalated with Co^{II} using the following procedure. Under ambient atmosphere, a 100 mL Pyrex autoclave jar was charged with 30 mg of as-synthesized, activated PCN-222 and a solution of anhydrous $CoCl_2$ (6 mg) in DMF (10 mL). The mixture was sonicated for 20 min, after which, it is placed in a pre-heated oven at 150 °C for 16 h. After 16 h, the resultant PCN-222(Co), is collected after cooling by centrifugation (20 mins, 4500 rpm, RCF = 3,169 g), washed by centrifugation three times using DMF (3 x 50 mL) followed by acetone (3 x 50 mL). After the third acetone wash, 50 mL of fresh acetone was added and allowed to stand for 14 h. After which the PCN-222(Co) precipitate is again washed with acetone (3 x 50 mL). After the final wash, the resultant orange colored PCN-222(Co) (Figure (S1b)) is activated under vacuum at 120 °C before characterization. Cobalt metalation was supported by SEM/EDS which shows a Zr:Co ratio of 2.6:1 vs the expected 3:1 based on theoretical structure.

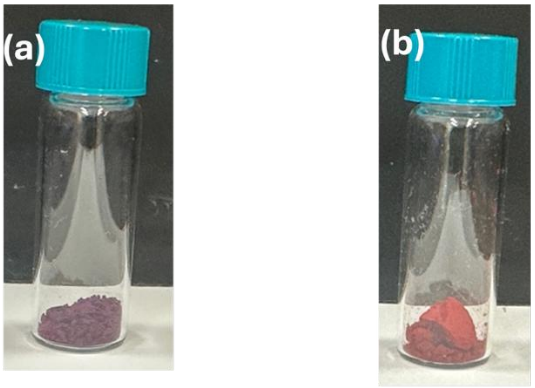

***Figure S1**: (a) As-synthesized PCN-222 (b) As-synthesized PCN-222(Co)*

2.2 Synthesis of Organic azides

***Safety Note*:** Sodium azide (NaN_3) and organic azides are potentially explosive, care should be taken when handling any azides. Small scale manipulations are recommended, and reaction should be done behind a shield in the fume hood. It is recommended that azide waste is diluted with inert diluent and carefully quenched before disposal.

Synthesis of 2,2,2-trichloroethyl carbonazidate (Troc-N_3)

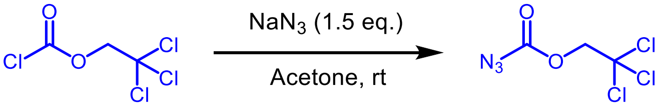

1

Compound **1** was synthesized by slight modification of literature procedure.[4,5] A 50 mL round bottom flask was charged with NaN_3 (6 mmol, 0.390 g, 1.5 equiv), 10 mL acetone, and a 1 cm PTFE magnetic stir bar. This solution was then cooled to 0 °C using an ice bath. Once cooled, a solution of 2,2,2-trichloroethylchloroformate (4 mmol, 0.847 g, 1 equiv) was added dropwise to the stirring suspension of the NaN_3 in acetone. The reaction was allowed to warm to room temperature over the course of 1 h and stirred at room temperature for 16 h. The solvent was then removed under reduced pressure using a rotary evaporator, then 20 mL each of DCM and H_2O was added to the dry solid. This mixture was extracted with DCM (3 x 20). The resultant organic layer was then dried with $MgSO_4$ and concentrated under reduced pressure using a rotary evaporator to obtain desired product (0.699 g, 80% yield). **^{1}H NMR (500 MHz, $CDCl_3$)** δ 4.79 (s, 2H). **^{13}C NMR (126 MHz, $CDCl_3$)** δ 156.53, 93.87, 76.51.

Synthesis of 4-methylbenzenesulfonyl azide (Tosyl- N_3)

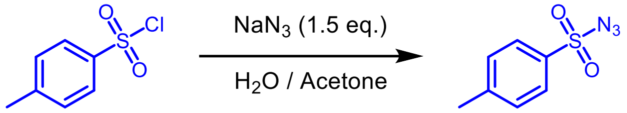

2

Compound **2** was synthesized through slight modification of literature procedure.[6] A 100 mL round bottom flask was charged with *p*-Toluenesulfonylchloride (5 mmol, 0.953 g, 1.0 equiv) acetone (25 mL) and a 1 cm PTFE magnetic stir bar. It was then cooled to 0 °C. Next, a NaN_3 solution (7.5 mmol, 0.486 g, 1.5 equiv in 25 mL of water) was added slowly to the stirring solution of p-toluenesulfonyl chloride. The reaction was stirred at 0 °C for 1 h, before warming to room temperature at which point it was stirred for an additional 3 h. Upon reaction completion, the solvent was removed under reduced pressure using a rotary evaporator. The crude residue was then extracted with Et_2O (3 x 20 mL). The combined organic layers were then dried with $MgSO_4$ and concentrated under reduced pressure to obtain the desired product as a colorless liquid (0.897 g, 91% yield). **^{1}H NMR (500 MHz, $CDCl_3$)** δ 7.71 (d, *J* = 6.5, 3.2 Hz, 2H), 7.30 (d, *J* = 8.4, 3.6 Hz, 2H), 2.36 (s, 3H). **^{13}C NMR (126 MHz, $CDCl_3$)** δ 146.38, 130.34, 127.48, 21.69.

Synthesis of 4-nitrobenzenesulfonyl azide (Nosyl- N_3)

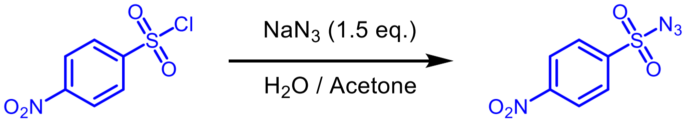

3

Compound **3** was synthesized through slight modification of the literature procedure.[6] A 100 mL round bottom flask was charged with, 4-nitrobenzenesulfonyl chloride (5 mmol, 1.108 g, 1.0 equiv), acetone (25 mL), and a 1 cm PTFE magnetic stir bar. This solution was then cooled to 0 °C. Next a NaN_3 solution (7.5 mmol, 0.486 g, 1.5 equiv in 25 mL of water) was added slowly into the rapidly stirring solution of Nosyl chloride. The reaction was stirred at 0 °C for 1 h, before warming to room temperature and stirred for 3 h at room temperature. The solvent was then removed under reduced pressure by rotary evaporation, and the crude reaction mixture was extracted with Et_2O (3x 20 mL). The combined organic layer is then dried with $MgSO_4$, filtered and concentrated with rotovap to obtain the desired product as an off-white solid (1.084 g, 95% yield). **^{1}H NMR (500 MHz, $CDCl_3$)** δ 8.46 (dd, *J* = 8.8, 1.7 Hz, 2H), 8.17 (dd, *J* = 8.7, 1.5 Hz, 2H). **^{13}C NMR (126 MHz, $CDCl_3$)** δ 151.25, 143.75, 128.93, 124.96.

Synthesis of methyl 4-azido-2,3,5,6-tetrafluorobenzoate (Fluoro-N_3)

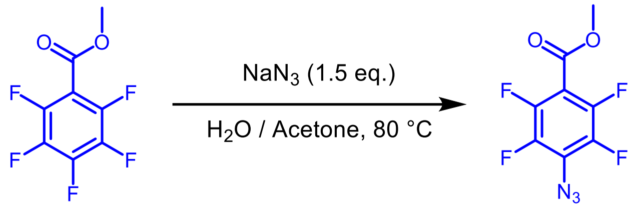

4

Compound **4** was synthesized with slight modification from the literature.[7,8] A 50 mL round bottom flask is charged with acetone (8 mL) and 3mL of H_2O and 1 cm PTFE magnetic stir bar. Methyl pentafluorobenzoate (2.2 mmol, 0.534 g, 1 equiv) was then added and the solution stirred for 30 min at 23 °C. Next, NaN_3 (3.3 mmol, 0.215 g 1.5 equiv.) was added slowly into the solution. The mixture was then stirred at 80 °C for 16 h at which point the reaction was complete as determined by TLC. After the completion of reaction, 10 mL of water is used to dilute the reaction, and the resulting mixture was extracted with Et_2O (20 mL x 3). The combined organic layers were dried using $NaSO_4$, filtered and concentrated using a rotary evaporator to obtain the desired product as off-white colored solid (0.537 g, 98%). **^{1}H NMR (500 MHz, $CDCl_3$)** δ 3.96 (s, 3H). **^{13}C NMR (126 MHz, $CDCl_3$)** δ 159.82, 145.33 (dm, J = 262.1 Hz), 140.42 (dm, J = 140.4 Hz), 107.52, 53.25. **^{19}F NMR (471 MHz, $CDCl_3$)** δ -138.67 (m, 2F), - 150.97 (m, 2F).

2.3 Synthesis of Aminated Products

Synthesis of 2,2,2-trichloroethyl (1-phenylethyl) carbamate

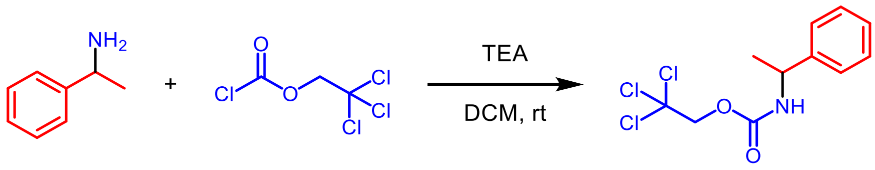

5

Compound **5** was synthesized with slight modification from the literature.[9,10] A 50 mL round bottom flask was charged with 2,2,2-trichloroethyl chloroformate (1 mmol, 0.218 g, 1 equiv), anhydrous triethylamine (TEA, 3 mmol, 0.304 g, 3 equiv), DCM (10 mL) and a 1 cm PTFE magnetic stir bar. The solution was then cooled to 0 °C in an ice bath. Next, 1-phenylethan-1-amine (1 mmol, 0.121 g 1 equiv) was added slowly to the DCM/TEA solution. The solution was allowed to warm up to room temperature and reaction progress was monitored by TLC. After the completion of reaction, 20 mL of water is used to dilute the reaction, and the resulting mixture was extracted with DCM (20 mL x 3). The combined organic layers were dried using $MgSO_4$, filtered, and concentrated using a rotary evaporator to obtain the desired product as off-white. (0.252 g, 85%). **^{1}H NMR (500 MHz, $CDCl_3$)** δ 7.29 – 7.21 (m, 4H), 7.20 – 7.15 (m, 1H), 5.24 (q, J = 7.9 Hz, 1H), 4.79 (s, 1H), 4.59 (s, 2H), 1.43 (d, J = 7.0 Hz, 3H). **^{13}C NMR (126 MHz, $CDCl_3$)** δ 153.78, 142.90, 128.75, 127.59, 126.00, 95.67, 74.53, 51.09, 22.26.

Synthesis of 4-methyl-*N*-(1-phenylethyl)benzenesulfonamide

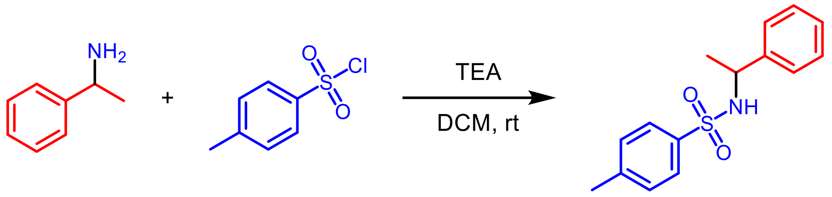

6

Compound **6** was synthesized with slight modification from the literature.[9,10] A 50 mL round bottom flask was charged with 1-phenylethan-1-amine (8.3 mmol, 1.005 g, 1 equiv), anhydrous TEA (25 mmol, 2.529 g, 3 equiv), anhydrous DCM (25 mL) and a 1 cm PTFE magnetic stir. The solution was then cooled to 0 °C. 4-methylbenzenesulfonyl chloride (10 mmol, 1.906 g, 1.2 equiv) was added and the reaction was stirred at 23°C for 16 h. After the completion of reaction, 20 mL of water is used to dilute the reaction, and the resulting mixture was extracted with DCM (20 mL x 3). The combined organic layers were dried using $MgSO_4$, filtered, and concentrated using a rotary evaporator to obtain the desired product a white colored solid (2.079 g, 91%). **^{1}H NMR (500 MHz, $CDCl_3$)** δ 7.65 – 7.60 (m, 2H), 7.17 (ddt, *J* = 6.2, 4.4, 2.3 Hz, 5H), 7.10 (dd, *J* = 7.6, 2.2 Hz, 2H), 5.31 (s, 1H), 4.46 (p, *J* = 6.9 Hz, 1H), 2.38 (s, 3H), 1.41 (dd, *J* = 6.9, 1.7 Hz, 3H). **^{13}C NMR (126 MHz, $CDCl_3$)** δ 143.08, 142.16, 137.71, 129.43, 128.50, 127.40, 127.12, 126.15, 53.67, 23.58, 21.48.

Synthesis of 4-nitro-*N*-(1-phenylethyl)benzenesulfonamide

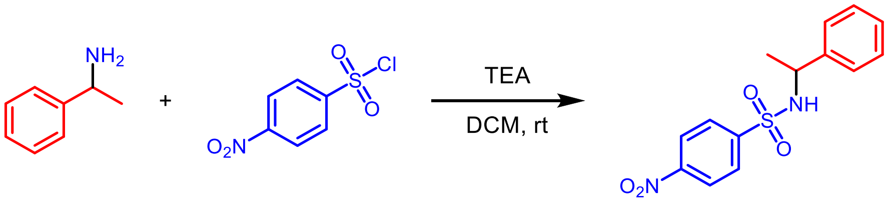

7

Compound **7** was synthesized with slight modification from the literature.[9,10] A 50 mL round bottom flask was charged with 1-phenylethan-1-amine (8.3 mmol, 1.005 g, 1 equiv), TEA (25 mmol, 2.529 g, 3 equiv), anhydrous DCM (25 mL) and a 1 cm PTFE magnetic stir. The solution was then cooled to 0 °C. Next, 4-nitrobenzenesulfonyl chloride (10 mmol, 2.216 g, 1.2 equiv) was added and the reaction was stirred at 23 °C for 16 h. After the completion of reaction, 20 mL of water is used to dilute the reaction, and the resulting mixture was extracted with DCM (20 mL x 3). The combined organic layers were dried using $MgSO_4$, filtered, and concentrated using a rotary evaporator to obtain the desired product an off-white colored solid. Analytical data are consistent with literature reports. **^{1}H NMR (500 MHz, DMSO-d_6)** δ 8.61 (d, *J* = 8.6 Hz, 1H), 8.26 – 8.19 (m, 2H), 7.87 – 7.81 (m, 2H), 7.18 – 7.07 (m, 5H), 4.49 – 4.40 (m, 1H), 3.35 – 3.31 (m, 1H), 1.28 (d, *J* = 7.0 Hz, 3H). **^{13}C NMR (126 MHz, DMSO-d_6)** δ 149.53, 147.53, 143.08, 128.56, 128.37, 127.32, 126.57, 124.56, 53.72, 23.98.

Synthesis of methyl 2,3,5,6-tetrafluoro-4-((1-phenylethyl)amino)benzoate

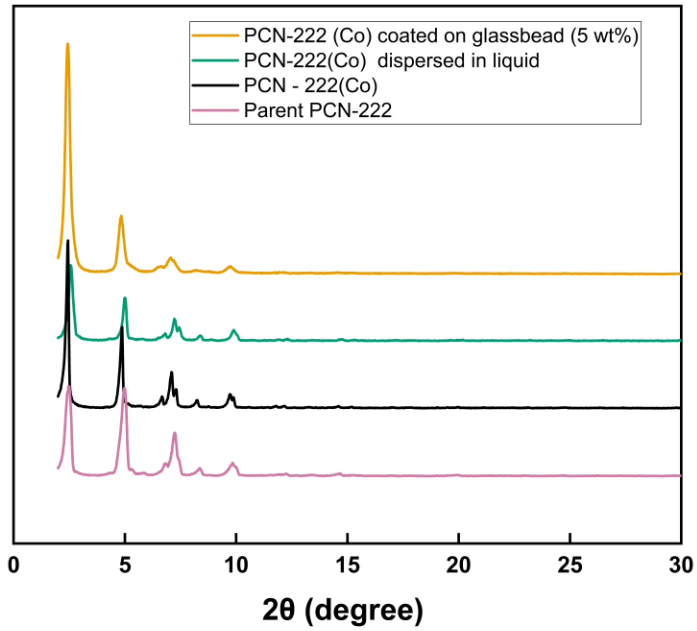

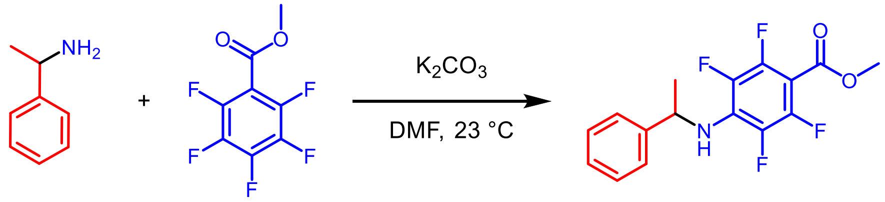

8

Compound **8** was synthesized using the following procedure.[11] A 50 mL round bottom flask was charged with 1-phenylethan-1-amine (5.0 mmol, 0.606 g, 1 equiv), methyl 2,3,4,5,6-pentafluorobenzoate (7.5 mmol, 1.695 g, 1.5 equiv), DMF (25 mL), K_2CO_3 (10 mmol, 1.382 g, 2 equiv) and a 1 cm PTFE magnetic stir. The reaction mixture is stirred at 23 °C and the reaction progress was monitored by TLC. Upon reaction completion, the reaction mixture is poured into ice water to precipitate product. The product is then collected by filtration, washed with cold water, then cold pentane. The desired product, a white solid, is then dried under reduced pressure using a rotary evaporator. (1.227 g, 75%). **^{1}H NMR (500 MHz, CDCl$_3$)** δ 7.36 (dd, *J* = 8.0, 6.8 Hz, 2H), 7.32 – 7.28 (m, 3H), 5.06 (p, *J* = 7.2 Hz, 1H), 4.57 – 4.48 (m, 1H), 3.91 (s, 3H), 1.61 (d, *J* = 6.8 Hz, 3H). **^{13}C NMR (126 MHz, CDCl$_3$)** δ 161.11 (t, *J* = 3.0 Hz), 146.42 (dm, *J* = 255.8 Hz), 143.73, 136.76 (dm, *J* = 239.4 Hz), 130.27 (tt, *J* = 11.4, 3.9 Hz), 128.88, 127.67, 125.50, 99.08 (t, *J* = 14.2 Hz), 54.60, 52.50, 24.58. **^{19}F NMR (471 MHz, CDCl$_3$)** δ -140.38 (m, 2F), -159.46 (m, 2F).

3.0 HTE Workflow Development

3.1 HTE solid reactor dosing (MOFBead)

We previously developed and optimized the MOFBead solid dispersion method for reactor dosing of MOF catalyst.[12] This method involves mixing known quantities of glass beads and MOF and loading into reactors using 3D-printed pre-calibrated scoops. While the glass dispersions are stable, the mechanical mixing is a liability with some MOFs. In the case of PCN-222(Co) prepared in our lab, a significant loss of surface area was observed (Figure S18). For this reason, an alternative solution-based dosing method similar to the method of Cohen et al. was developed.[13]

3.2 Dispersion reactor dosing

General Overview. MOF was dosed into reactor vials via stock solution with the aid of an Opentrons OT2 liquid handling robot. Stock solutions of MOF dispersion in solvent were prepared by mixing a measured amount of MOF with a precise quantity of solvent. For this project, THF was the easiest to handle as other potentially suitable solvents such as DCM or Et_2O were too low viscosity for the pipetting process (they dripped out of the pipette). The suspended MOF mixture was sonicated for 20 mins to evenly disperse the MOF in the solvent before delivery to the vial inserts. The resulting dispersion was then pipetted into reactor vials (1 mL, 2 mL, and 4 mL) inserts in high-throughput reactor blocks (24-well, 48-well, and 96-well plates) with continuous stirring. For example, 6 mg of MOF can be mixed with 12 mL of THF and sonicated for 20 mins to obtain a stock solution which delivers 0.25mg or 0.1mg of MOF to each well upon pipetting of 500 µL, or 200 µL into reactor vials, respectively (figure S2). After pipetting, the MOFs are re-activated in the reactor vials under vacuum with heating in a vacuum oven (16 h, 150 °C) before use in catalyst screens. The accuracy and precision of pipetting was characterized by plate reader in accordance with our previous report (figure S3 and S4).[12] Preservation of the MOF structure was evaluated via powder x-ray diffraction and N_2 adsorption isotherms (figure S15)

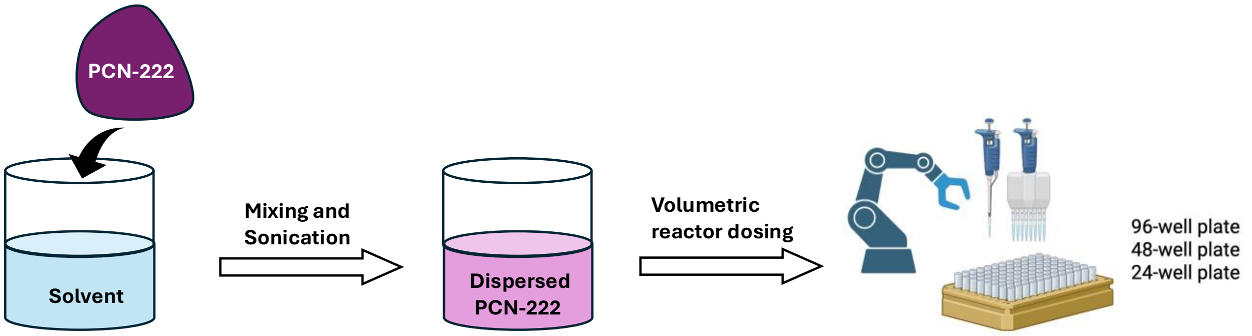

Figure S2 *Workflow for MOF solution-based dosing*

Note: In principle, any solvent that can be efficiently evacuated from the MOF without compromising its structure can be used to disperse the MOF. In practice, however, both the viscosity and density of the solvent affect optimization of the automated pipetting. High density but low viscosity solvents such as dichloromethane can be difficult to process as they drip out of

pipette as it moves between reactor wells. For these studies, we found that THF had the best mix of volatility, low density, and ease of removal but note that for other applications with other MOFs, this may not be the case and should be carefully considered.

Brief overview of reactor dosing precision characterization: The accuracy and precision of solution reactor dosing was characterized using our previously developed plate reader method.[12] In this method, PCN-222(Co) was dosed into 4 mL vials (24 vials, 250 µg of PCN-222(Co)) and activated to remove THF solvent. Next, 1 mL of DMSO (10 vol% NBu_4OH (40% v/v in H_2O) was added to all 24 vials. 3x5 mm PTFE magnetic stir bars were added, and the reactor block was then sealed and PCN-222(Co) allowed to digest at 60 °C, and 500 rpm for 16 hours. After 16 hours, the solutions are diluted with DMSO by a factor of 10 (25 µg theoretical per well). The absorbance of the resultant solutions were recorded using the plate reader at λ_{max}. Experimental yields were determined using a standard calibration curve (Figure S3) plotted from an accurately weighed sample and digested.

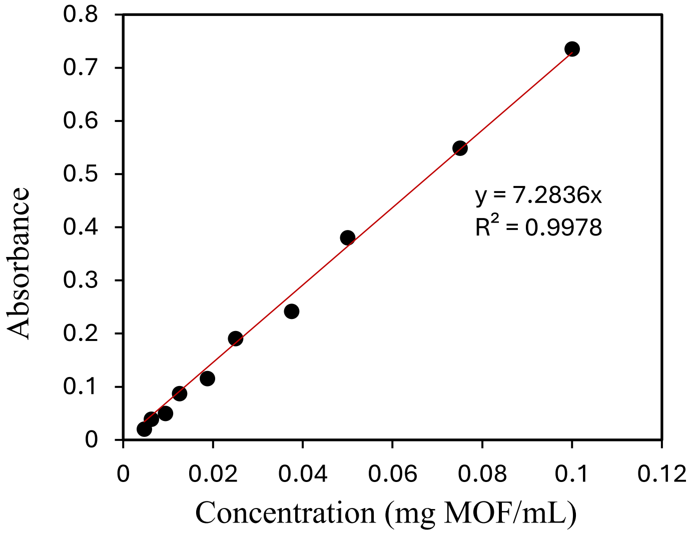

***Figure S3**: Standard calibration curve of PCN-222(Co) following digestion of known quantities of PCN-222*

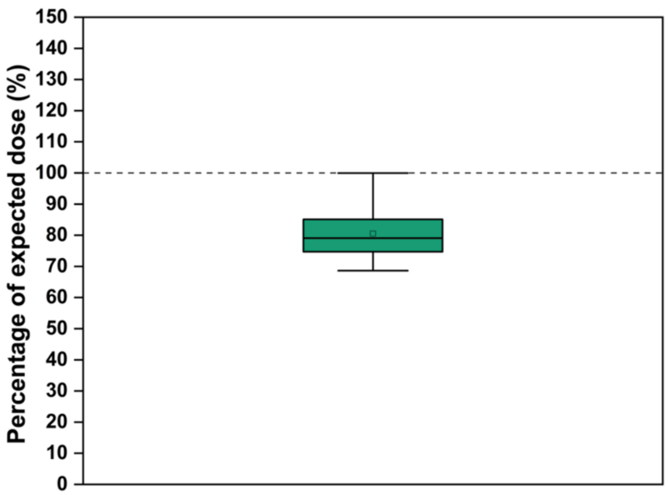

***Figure S4**: Box plot showing the efficiency of the pipetted MOF dispersion (24 samples). Delivering 250 µg of PCN-222(Co) per well with an accuracy of 80% of expected and a coefficient of variation of 10.3%*

3.3 Procedure for Parallel Optimization

Reactions are set-up in either 4 mL or 2 mL vials using 24-well or 48-well reactor blocks respectively. Upon reaction completion, the reactions were allowed to cool to room temperature, the reactor block top removed, then using the OT2 liquid handling robot, each reaction vial was diluted with 1 mL of THF. Each reaction mixture was then pipetted into a 96-well filtration set-up made of 96-well filter plates, (2mL, glass fiber, 5.0µm) and 2 mL 96-well receiver using the liquid handling robot. Next, 1 mL of fresh THF was added to the reaction vials, mixed by drawing the THF into then out of the pipette multiple times, then transferred to the filter plate. To accelerate the filtration, a 3D-printed manifold was used to apply overhead pressure sourced from a fish tank oxygenator (Figure S5). The filtrates are then loaded into GC-MS vials and characterized by GC-MS and/or NMR.

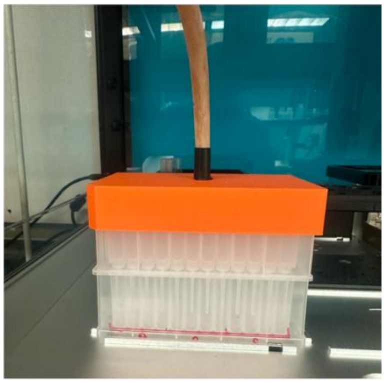

***Figure S5:** filtration set-up showing filter, receiver and our customized manifold for delivering overhead pressure to the filter apparatus*

4.0 Catalysis

4.1 Reaction Screen Conditions

Catalytic reactions were performed both under air on the bench and in inert atmosphere of N_2, in the glovebox. The relevant conditions are stated for each experiment. Though the dispersion dosing method was validated to work down to 100 μg/reaction scales, the following assays were set up at ~3mg of catalyst per reaction to facilitate additional analyses and minimize complications from small solvent volumes, a known issue in high-throughput reaction screening.[14]

4.1.1 High-throughput Reaction (HTE) Screen

In the glovebox, 24 4 mL vials loaded with activated PCN-222(Co) (0.003 mmols, 3.7 mg, 5 mol % Co) in a 24-well aluminum reaction block were charged with 3×5 mm PTFE stir bars, 4 Å molecular sieve (15 mg), ethylbenzene (0.6 mmol, 63.7 mg, 10 equiv), 0.2 mL of solvent (Figure S6), 1 equiv of azide (Troc-N_3 (13.1 mg), Tosyl- N_3 (11.8 mg), Nosyl- N_3 (13.7 mg) and Fluoro-N_3 (14.9 mg)) from stock solutions in 0.1 mL of the respective solvents. Note that the azide is always added last to the mixture of ethylbenzene, MOF, and solvent. The aluminum reaction block is sealed and allowed to react at 23 °C with stirring (350 rpm) for 12 hours. This process is repeated with three (3) other aluminum reaction blocks, allowing reactions at three additional temperatures (50 °C, 80 °C and 120 °C). After 12 hours, the reactions are worked up as described in the procedure for parallel optimization (section 3.3). In short, the samples were diluted with THF, transferred to a filter plate, the wells were washed with an additional 1 mL of THF, then these were added to the filter plate, before filtration. The liquid handling robot was also used to dilute the reactions for subsequent GC-MS analysis.

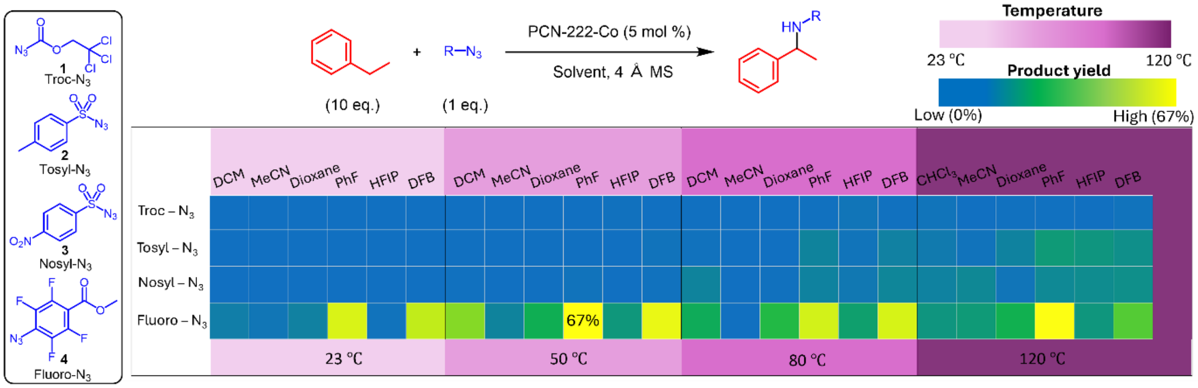

Figure S6: *Heatmap showing the initial reaction screen using 4 azides, 6 solvents at 4 different temperatures.*

As shown in figure S6 above, Fluoro-N_3 had the best yield at all four temperatures as determined by GC-MS area (figure S7) as an approximate measure of yield. As described below, authentic products were shown to have similar response factors by MS. However, because GC-MS is only semi-quantitative, to further validate the results of the GC-MS assay, a sampling of high and low yielding conditions by GC-MS were also evaluated by NMR. In all cases, the GC yields were in good qualitative agreement with the quantitative NMR yields.

The NMR yields of the best conditions with Troc-N_3, Tosyl-N_3 and Nosyl-N_3 were all below 10%. Fluoro-N_3 yielded better yields of product with the best yield being 67% in PhF as determined by ^{19}F NMR. Fluoro-N_3 was therefore chosen as the azide for our study with PhF and 50 °C as selected solvent and temperature respectively.

4.1.2 GC-MS method

Figure S7 shows the GC-MS method used to characterize our catalysis reactions.

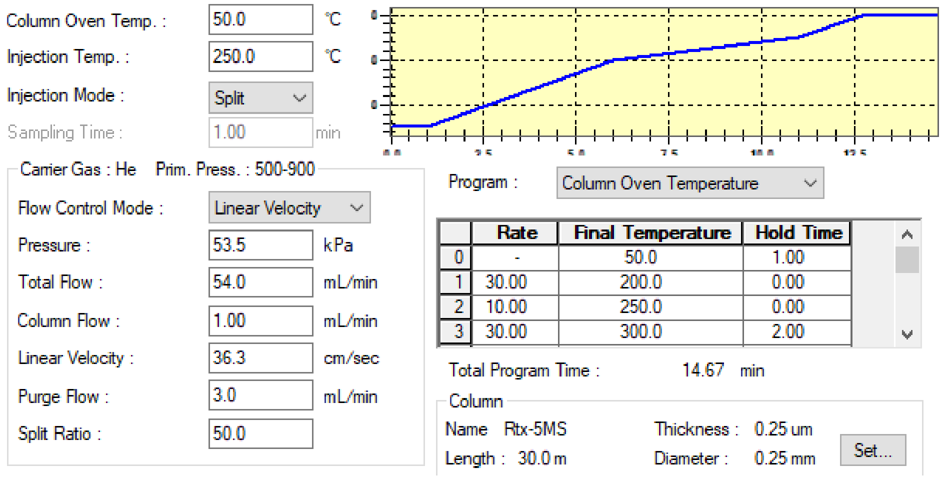

Figure S7: *GC-MS method used to characterize catalysis*

4.1.3 Aminated product yield by GC-MS

(a)

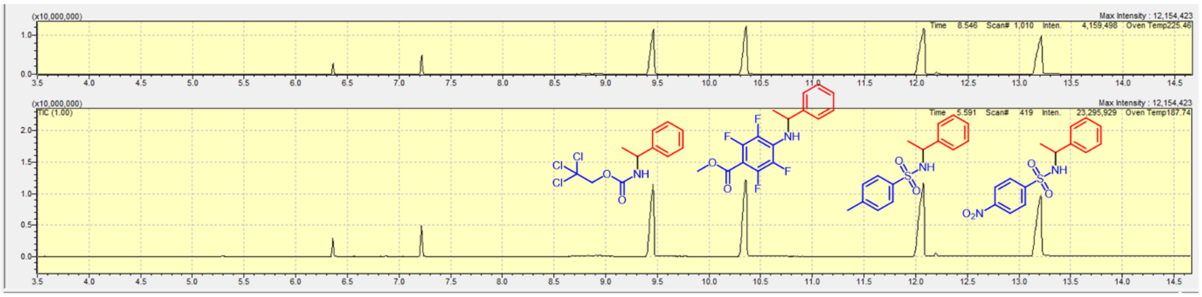

(b)

	23 °C						50 °C						80 °C						120 °C					
	DCM	MeCN	Dioxane	PhF	HFIP	DFB	DCM	MeCN	Dioxane	PhF	HFIP	DFB	DCM	MeCN	Dioxane	PhF	HFIP	DFB	$CHCl_3$	MeCN	Dioxane	PhF	HFIP	DFB
Troc – N_3	0	0	0	0	0	0	3E+05	0	6E+05	2E+05	3E+06	2E+05	1E+06	2E+05	5E+05	2E+06	8E+06	3E+06	7E+06	3E+05	2E+06	2E+06	1E+06	5E+06
Tosyl – N_3	0	0	0	0	0	0	2E+06	3E+05	1E+06	1E+06	6E+05	7E+06	5E+06	0	8E+05	3E+07	7E+06	3E+07	2E+07	6E+06	3E+07	7E+07	6E+07	5E+07
Nosyl – N_3	0	0	0	0	0	0	2E+06	0	1E+06	7E+06	5E+05	4E+06	4E+07	4E+05	3E+06	3E+07	1E+07	4E+07	3E+07	4E+07	9E+06	4E+07	5E+07	5E+07
Fluoro – N_3	2E+07	1E+07	3E+07	2E+08	5E+06	2E+08	2E+08	3E+07	1E+08	2E+08	7E+07	2E+08	1E+08	1E+06	1E+08	2E+08	8E+07	2E+08	6E+07	7E+07	1E+08	2E+08	7E+07	1E+08

Figure S8: *(a) GC chromatogram of authentic aminated products, used to estimate reaction yield (b) actual GC-peak areas of the HTE reaction screen in section 4.1.1 above*

To estimate the products yields of our catalysis reactions with the different azides, we synthesized authentic products using simple amine coupling (section 2.3). We then prepared a GC-MS sample containing equal amount of each potential aminated product (0.03 mmol) at concentrations expected at the end of catalysis. Figure S8 shows that the GC peak areas (sensitivity) of the four different products are similar. Table S1 shows the exact areas of each products. The similarities in the sensitivity of the different products means that we can roughly compare the GC peak area of the four aminated products after catalysis to identify the best yield which we can validate by NMR. In all cases, NMR yields were consistent with approximate yields by GC but we note that the GC yields should not be taken to be exact.

Table S1: *The GC-MS area of the 0.03 mmol of the different aminated products.*

Product	Retention time (Mins)	GC-MS Area
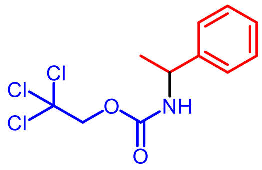 **5**	9.58	1.57E+08
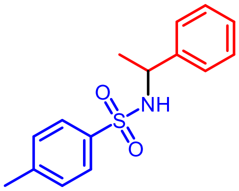 **6**	12.25	1.58E+08
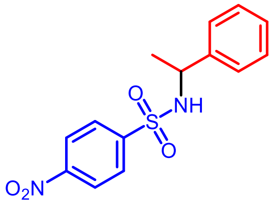 **7**	13.42	2.19E+08
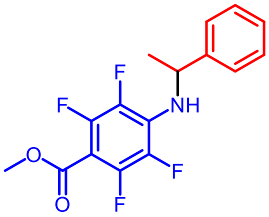 **8**	10.49	1.94E+08

4.2 Reaction Optimization

Catalyst loading experiment:

Having identified PCN-222(Co) as a catalyst for benzylic amination using Fluoro-N_3. Next, we evaluated catalyst loading (Table S2). Five (5) 4 mL scintillation vials were transferred into the glovebox. One of the vials was empty while the other four contains different quantities of pre-dosed PCN-222(Co) (0.074 mg (0.1 mol%, estimated), 0.4 mg (0.5 mol%), 0.7 mg (1 mol%), 3.7 mg (5 mol%) using the general procedure in section 3.2 above. Note that Co content was determined relative to Zr based on SEM-EDX. The vials were charged with 3x5 mm PTFE

magnetic stir bars, molecular sieve (4 Å, 15 mg), ethylbenzene (0.6 mmol, 63.7 mg, 10 equiv) and Fluoro-N_3 (14.9 mg, 0.06 mmol in 0.1 mL of PhF) was added via stock solution. More PhF (0.2 mL) was added, then the vials are capped, and allowed to react in the glovebox at 50 °C with stirring (350 rpm). After 12 hours, the reactions are allowed to cool, solvent was removed under reduced pressure in the vacuum oven. Then internal standard was added in $CDCl_3$ and allowed to stir for 20 min. The resulting solution is then centrifuged and the supernatant characterized by quantitative ^{19}F NMR.

As shown in table S2, catalyst loadings down to 0.5% gave yields above 50% using ethylbenzene in excess as a model substrate. Lower loadings resulted in high turnover numbers (~200) but with overall poor conversion. Though 1 mol% gave good yields of the aminated product, 5% loading was used in the substrate scope to facilitate good conversion with more challenging substrates and because it was expected to be more effective for substrate-limiting cases (Condition A). For practical application of these catalysts, however, these data suggest that lower catalysts loadings may be well tolerated and should be explored on a substrate-by-substrate basis.

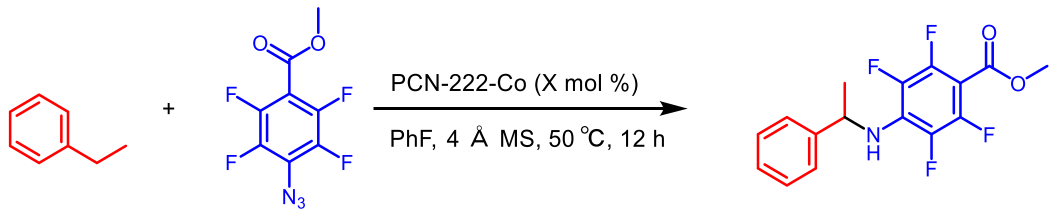

Table S2: *Optimization table with different control reactions. Yields are NMR yields*

Entry	Catalyst Loading (Mol %)	NMR Product Yield (%)
1	No catalyst	-
2	0.1*	20
3	0.5	51
4	1	80
5	5	88

*estimated as this is slightly outside the validated window for slurry reactor dosing.

Evaluation other MOFs structurally similar to PCN-222 (Co)

To confirm that amination is catalyzed by the unique structure of PCN-222(Co) (porphyrin linker) and not just surface deposited Co^{II} or SBU Zr centers, we carried out control reactions using other MOFs having similar structure to PCN-222(Co). NU-1000 was selected as the model material to test if surface deposited cobalt is responsible for the catalysis as it has the same topology, same SBU, and similar pore size to PCN-222.

As shown in table S3, no reaction was observed with both un-metalated PCN-222 and NU-1000. We also carried out post-synthetic modification (PSM) of NU-1000 with $CoCl_2$ using similar procedure as the metalation of PCN-222 (section 2.1). No conversion of the azide or formation of

the aminated product was observed with the PSM NU-1000. Collectively, these results suggest that the activity of the PCN-222(Co) is due to cobalt metalated in the porphyrin, not cobalt deposited on the SBU.
Note: this reaction was done in the glovebox using the procedure as in the catalyst loading experiment.

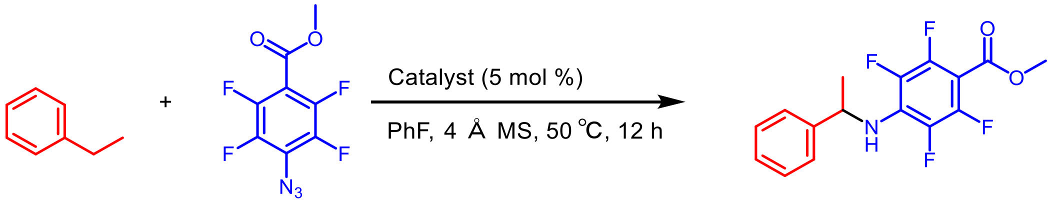

***Table S3:** Optimization table with different control reactions*

Entry	**Catalyst**	**Yield/remark**
1	PCN-222 (no cobalt)	No product
2	NU-1000 MOF	No product
3	NU-1000(Co)	No product

Comparisons of PCN-222(Co) to Molecular Analogs

To test if site isolation confers air and moisture tolerance, standard ethyl benzene amination reactions were carried out under air using four different complexes: PCN-222(Co), Co(TPP), CoCl(TPP) and Co(TMCPP). These molecular analogs were chosen on the basis of their prevalence in this class of reaction (CoTPP) or their electronic similarity to the porphyrin linker in the MOF Co(TMCPP). This experiment was set-up using our general procedure, but with bench solvent under ambient atmosphere. 4 mL scintillation vials were charged with 5 mol% of the different catalysts of interest (2.0 mg of Co(TPP), 2.5 mg of CoCl (TPP), 2.5 mg of Co (TMCPP) and 3.7 mg of PCN-222(Co)), 3x5 mm PTFE magnetic stir bars, molecular sieve (4 Å, 15 mg), ethylbenzene (0.6 mmol, 63.7 mg, 10 equiv), 0.2 mL PhF, and Fluoro-N_3 (14.9 mg, 0.06 mmol, in 0.1 mL PhF) was added via stock solution via micropipette. The vials are capped, and allowed to react for 12 hours at 50 °C with stirring (350 rpm). After 12 hours, the reactions are allowed to cool, solvent was removed under reduced pressure in the vacuum oven. Then internal standard was added in $CDCl_3$ and allowed to stir for 20 min. The resulting solution is then centrifuged and the supernatant characterized by quantitative ^{19}F NMR.

To confirm that the MOF activity is not likely due to an oxidized Co(porphyrin analog), presumably CoX(porphrin) the activity of the MOF (PCN-222(Co)) was also compared to CoCl(TPP).[1] As shown in figure S9 and S10, the CoCl(TPP) catalyst was also inactive under

aerobic/moist conditions, indicating that observed activity is indeed related to confinement within the MOF.

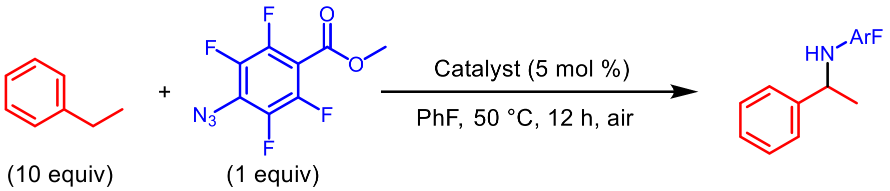

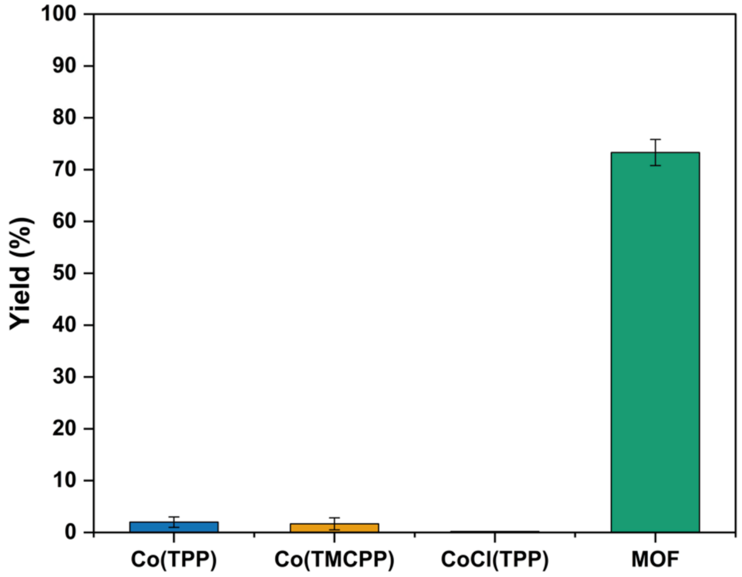

Figure S9: *Reaction under air. Yields are* ^{19}*F NMR yields using 4,4-difluorobiphenyl as internal standard*

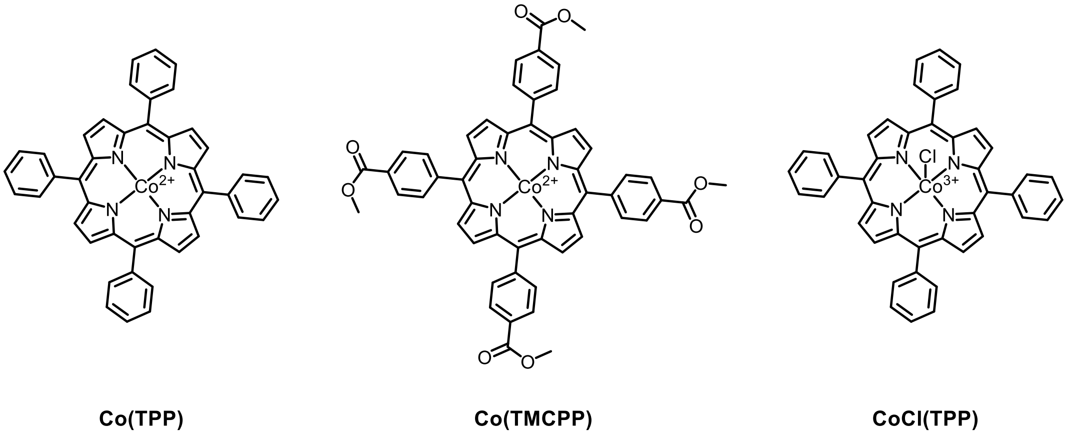

Figure S10*: Structures of molecular catalysts used for compariso*

Reaction in the presence of H_2O

Having observed the superior activity of PCN-222(Co) catalyst over the molecular catalyst in the presence of air, we also hypothesized that the site isolation in our PCN-222(Co) catalyst will also make is more robust in withstanding H_2O. Air/water sensitivity are generally recognized limitation in related nitrene-transfer C–H amination chemistry, although some sterically bulky Co porphyrin systems are unusually robust.[15]

Procedure: Outside of the glovebox, 4 mL scintillation vials were charged with 5 mol% of the respective cobalt catalyst, 3x5 mm PTFE magnetic stir bars, ethylbenzene (0.6 mmol, 63.7 mg, 10 equiv), 0.2 mL PhF, and Fluoro-N_3 (14.9 mg, 0.06 mmol, in 0.1 mL PhF) was added via stock solution via micropipette followed by 10 µL of H_2O. The vials were capped, and allowed to react for 12 hours at 50 °C with stirring (350 rpm). After 12 hours, the reactions were allowed to cool, solvent was removed under reduced pressure in the vacuum oven. Then internal standard was added in $CDCl_3$ and allowed to stir for 20 min. The resulting solution is then centrifuged and the supernatant characterized by quantitative ^{19}F NMR.

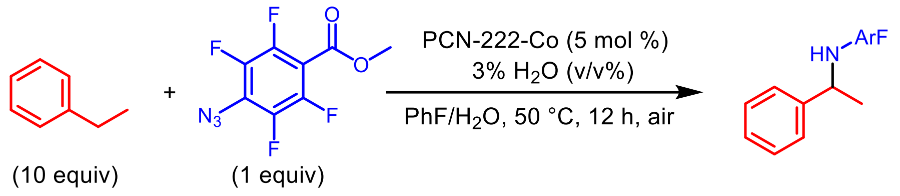

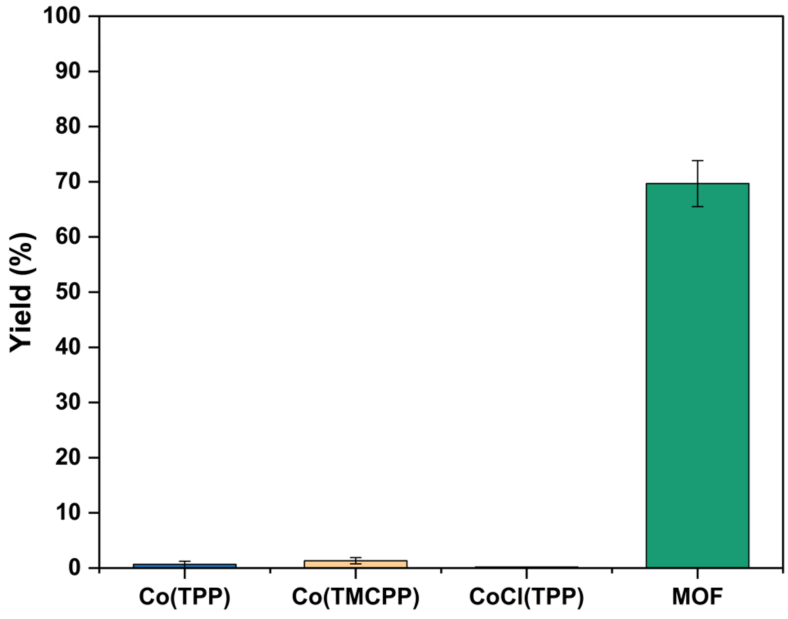

Figure S11: *Reaction containing 3% v/v of H_2O. Yields are ^{19}F NMR yields using 4,4-difluorobiphenyl as internal standard*

Yield Dependence on Bulk Water

In this experiment, the impact of bulk water on reactions yields was examined by conducting the standard experimental conditions but with different volume fractions of water in PhF/H_2O mixtures, again under air. Reactions were repeated in triplicate as some variability was noted between reactions.

Procedure: Outside of the glovebox, 4 mL scintillation vials were charged with 5 mol% of the respective cobalt catalyst, 3x5 mm PTFE magnetic stir bars, ethylbenzene (0.6 mmol, 63.7 mg, 10 equiv) and Fluoro-N_3 (14.9 mg, 0.06 mmol, 1 equiv) with the 0.3 mL of the respective solvent mixture. The vials were capped, and allowed to react for 12 hours at 50 °C with stirring (350 rpm). After 12 hours, the reactions were allowed to cool, solvent was removed under reduced pressure in the vacuum oven. Then internal standard was added in $CDCl_3$ and allowed to stir for 20 min. The resulting solution is then centrifuged and the supernatant characterized by quantitative ^{19}F NMR.

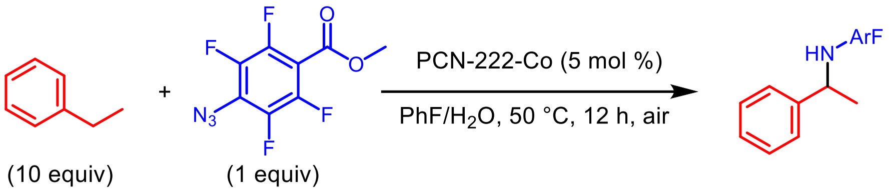

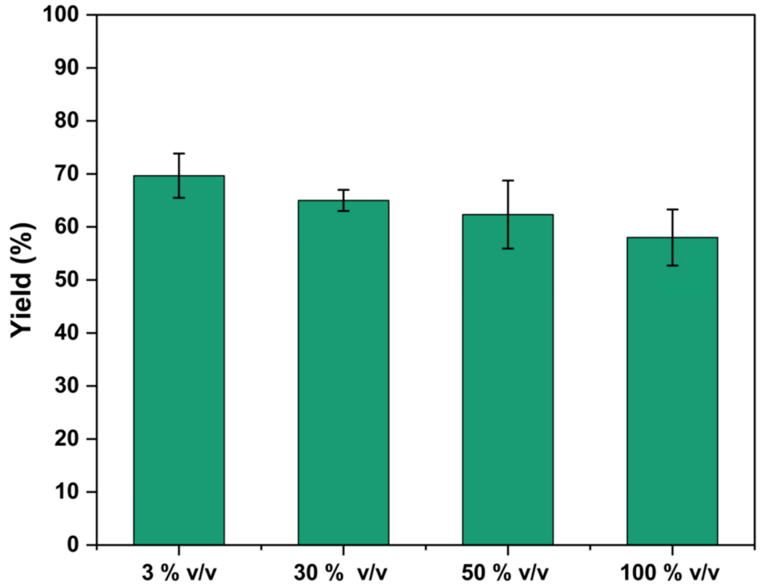

Figure S12: *Impact of bulk water on PCN-222(Co) catalyzed reaction. Yields are ^{19}F NMR yields using 4,4-difluorodiphenyl as internal standard*

As shown in figure S12, the yields monotonically decrease with increasing water volume fraction of the solvent. We note, however, that these data should not be interpreted as aqueous catalysis as the ethylbenzene is likely to partition into the MOF pore, making the water activity of the catalytic microenvironment considerably lower than the bulk solvent. Nonetheless, it shows that water does not catastrophically inhibit catalysis.

Bench Stability Test

In this experiment, a batch of catalyst stored on the bench in a 20 mL scintillation vial was periodically assayed under the standard conditions. Note that the catalyst was stored on the bench under air/moisture but the catalyst was assayed under N_2 atmosphere and with molecular sieves. High activity was also seen under aerobic conditions but these were not assayed as extensively due to the highly time consuming nature of this experiment.

Outside the box, 4 mL scintillation vials were charged with 3.7 mg of PCN-222(Co), 3x5 mm PTFE magnetic stir bars. Then the vials were brought in the glovebox and charged with molecular sieve (4 Å, 15 mg), ethylbenzene (0.6 mmol, 63.7 mg, 10 equiv), 0.2 mL PhF, and Fluoro-N_3 (14.9 mg, 0.06 mmol, in 0.1 mL PhF) via micropipette as a stock solution. The vials were capped, and allowed to react for 12 hours at 50 °C with stirring (350 rpm). After 12 hours, the reactions were allowed to cool, solvent was removed under reduced pressure in the vacuum oven. Then internal standard was added in $CDCl_3$ and allowed to stir for 20 min. The resulting solution is then centrifuged and the supernatant characterized by quantitative ^{19}F NMR.

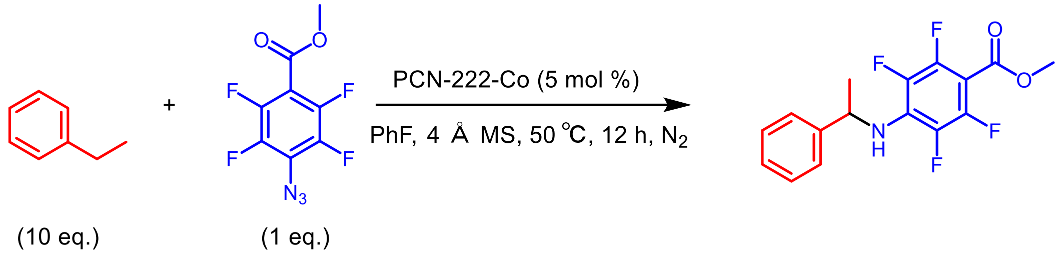

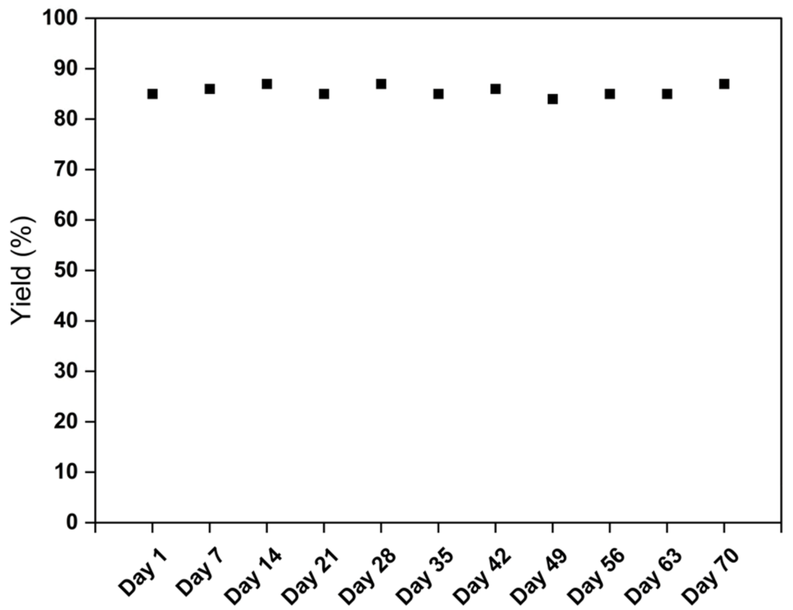

Figure S13: *Bench stability study using 10 equivalents of C–H bond. Yields are* ^{19}*F NMR yields using 4,4-difluorobiphenyl as internal standard*

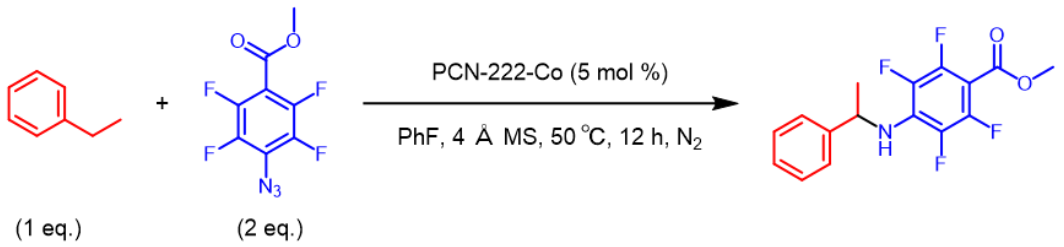

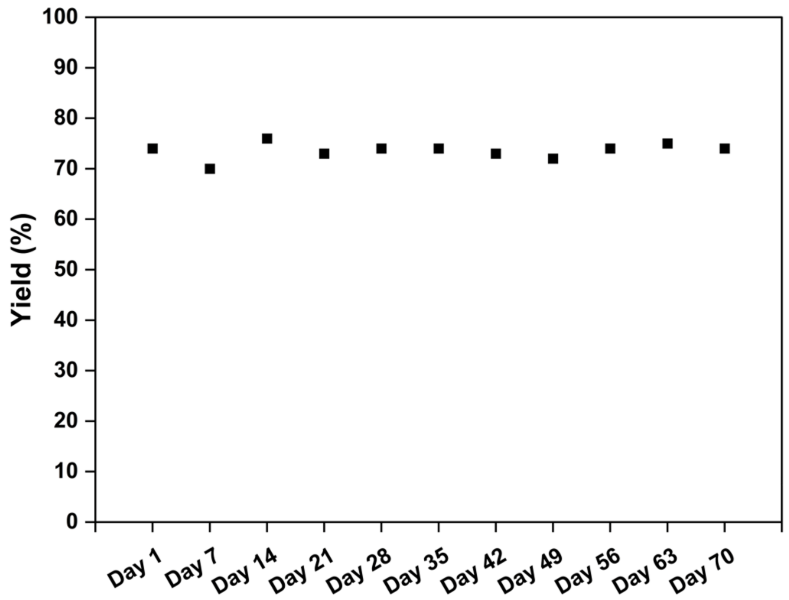

Figure S14: *Bench stability study using conditions A. Yields are ^{19}F NMR yields using 4,4-difluorobiphenyl as internal standard*

4.3 Substrate Scope

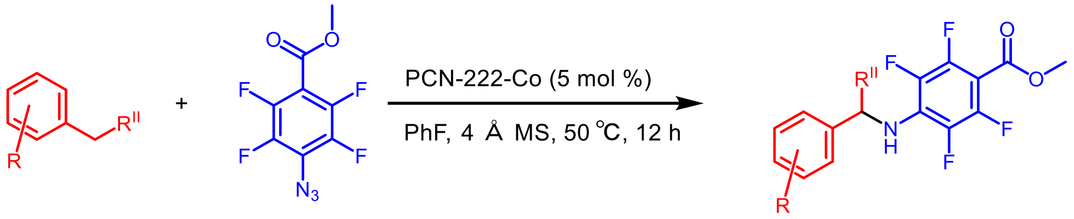

4.3.1 <u>General procedure:</u>

Procedure A – limiting C-H

Using a 24-well aluminum reaction block, 4 mL scintillation vials were charged with 3×5 mm PTFE magnetic stir bars, PCN-222(Co) catalyst (5 mol%, 0.003 mmol, 3.7 mg, pre-dosed into vials using liquid handler dosing methods), molecular sieve (4 Å, 15 mg), C-H substrate (0.06 mmol, 1 equiv), PhF (0.2 mL), and Fluoro-N_3 (30.1 mg, 0.12 mmol in 0.1 mL of PhF) in that order. The reaction block is then sealed, and the reaction mixture is allowed to react (50 ºC, 350 rpm, 12 h). After 12 hours, the reaction is stopped and allowed to cool to room temperature. The solvent is then removed under reduced pressure in the vacuum oven. The crude residues from each of these reactions was then loaded directly on to silica for purification.

Procedure B – 10 equiv of C-H

Using a 24-well aluminum reaction block, 4 mL scintillation vials were charged with 3×5 mm PTFE magnetic stir bars, PCN-222(Co) catalyst (5 mol%, 0.003 mmol, 3.7 mg, as pre-dosed in vials using liquid handler dosing methods), molecular sieve (4 Å, 15 mg), C-H substrate (0.6 mmol, 10 equiv), PhF (0.2 mL), and Fluoro-N_3(14.9 mg, 0.06 mmol in 0.1 mL of PhF) . The reaction block is then sealed, and the reaction mixture is allowed to react (50 ºC, 350 rpm, 12 h). After 12 hours, the reaction is stopped and allowed to cool to room temperature. The solvent is then removed under reduced pressure in the vacuum oven. The crude residues from each of these reactions was then loaded directly on to silica for purification.

Note: Both condition A and B were set-up using bench solvent under ambient conditions and molecular sieve was used which was shown to improve yield by 5-10 %

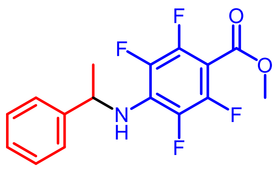

8

Methyl-2,3,5,6-tetrafluoro-4-((1-phenylethyl)amino)benzoate

Compound 8 is prepared using both general procedures A and B above. Purified by flash chromatography (ethyl acetate/hexane 2-20%) to obtain the product as a white solid (13.5mg, 69% for procedure A and 15.7 mg, 80% yield for procedure B).

^{1}H NMR (500 MHz, $CDCl_3$) δ 7.36 (dd, *J* = 8.0, 6.8 Hz, 2H), 7.32 – 7.28 (m, 3H), 5.06 (p, *J* = 7.2 Hz, 1H), 4.57 – 4.48 (m, 1H), 3.91 (s, 3H), 1.61 (d, *J* = 6.8 Hz, 3H).

^{13}C NMR (126 MHz, CDCl3) δ 161.08 (t, *J* = 3.0 Hz), 146.42 (dm, *J* = 255.8 Hz), 143.73, 136.76 (dm, *J* = 239.4 Hz), 130.27 (tt, *J* = 11.4, 3.9 Hz), 128.88, 127.67, 125.50, 99.08 (t, *J* = 14.2 Hz), 54.60, 52.50, 24.58.

^{19}F NMR (471 MHz, $CDCl_3$) δ -140.38 (m, 2F), -159.46 (m, 2F).

HRMS (ESI-TOF) m/z: $[M - H]^-$ Calculated for $[C_{16}H_{12}O_2NF_4]^-$: 326.0810, found: 326.0813

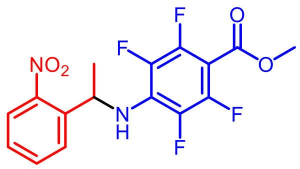

9

Methyl-2,3,5,6-tetrafluoro-4-((1-(2-nitrophenyl)ethyl)amino)benzoate
Compound 9 is prepared using both general procedures A and B above. Purified by flash chromatography (ethyl acetate/hexane 2-20%) to obtain the product as a yellowish solid (9.8 mg, 44% for procedure A and 16.3 mg, 73% yield for procedure B).
^{1}H NMR (500 MHz, CDCl$_3$) δ 7.95 (dd, *J* = 8.2, 1.3 Hz, 1H), 7.60 (td, *J* = 7.6, 1.3 Hz, 1H), 7.51 (dd, *J* = 7.9, 1.5 Hz, 1H), 7.42 (ddd, *J* = 8.5, 7.2, 1.5 Hz, 1H), 5.66 – 5.57 (m, 1H), 4.68 – 4.63 (bs, 1H), 3.87 (s, 3H), 1.70 (d, *J* = 6.6 Hz, 3H).
^{13}C NMR (126 MHz, CDCl$_3$) δ 160.87, 148.22, 146.39 (dm, *J* = 268.38 Hz), 139.70, 136.46 (dm, *J* = 243.2 Hz), 133.72, 129.33 (m), 128.32, 126.85, 125.15, 99.52, 52.57, 50.73, 24.35.
^{19}F NMR (471 MHz, CDCl$_3$) δ -139.75 (m, 2F), -160.39 (m, 2F).
HRMS (ESI-TOF) m/z: [M - H]$^-$ Calculated for $[C_{16}H_{11}O_4N_2F_4]^-$: 371.0660, found: 371.0662

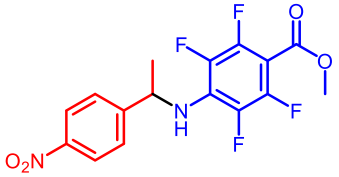

10

Methyl-2,3,5,6-tetrafluoro-4-((1-(4-nitrophenyl)ethyl)amino)benzoate
Compound 10 is prepared using both general procedures A and B above. Purified by flash chromatography (ethyl acetate/hexane 2-20%) to obtain the product as a yellowish solid (14.5 mg, 65% for procedure A and 19.9 mg, 89% yield for procedure B).
^{1}H NMR (500 MHz, CDCl$_3$) δ 8.22 – 8.15 (m, 2H), 7.48 – 7.42 (m, 2H), 5.15 – 5.06 (m, 1H), 4.56 (dt, *J* = 7.6, 3.2 Hz, 1H), 3.87 (s, 3H), 1.61 (d, *J* = 6.8 Hz, 3H).
^{13}C NMR (126 MHz, CDCl$_3$) δ 160.81 (q, *J* = 3.0 Hz), 151.39, 147.41, 146.35 (dm, *J* = 257.1 Hz), 136.62 (dm, *J* = 239.4 Hz), 129.43 (tt, *J* = 11.3, 3.8 Hz), 126.40, 124.23, 99.85 (t, *J* = 14.3 Hz), 54.28 (t, *J* = 4.2 Hz), 52.60, 24.68.
^{19}F NMR (471 MHz, CDCl$_3$) δ -139.69 (m, 2F), -159.35 (d, *J* = 16.4 Hz, 2F).
HRMS (ESI-TOF) m/z: [M - H]$^-$ Calculated for $[C_{16}H_{11}O_4N_2F_4]^-$: 371.0660, found: 371.0662

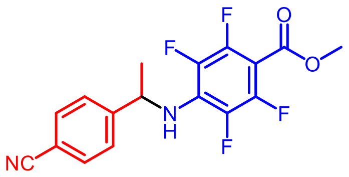

11

Methyl-4-((1-(4-cyanophenyl)ethyl)amino)-2,3,5,6-tetrafluorobenzoate

Compound 11 is prepared using both general procedures A and B above. Purified by flash chromatography (ethyl acetate/hexane 2-20%) to obtain the product as an off-white colored solid (16.9 mg, 80% for procedure A and 18.0 mg, 85% yield for procedure B).

^{1}H NMR (500 MHz, CDCl$_3$) δ 7.66 – 7.61 (m, 2H), 7.41 – 7.36 (m, 2H), 5.05 (p, J = 7.1 Hz, 1H), 4.45 (d, J = 8.3 Hz, 1H), 3.89 (s, 3H), 1.59 (d, J = 6.8 Hz, 3H).

^{13}C NMR (126 MHz, CDCl$_3$) δ 160.83, 149.29, 146.40 (dm, J = 259.2 Hz), 136.63 (dm, J = 223.7 Hz), 132.81, 129.43, 126.27, 118.50, 111.65, 99.89 (t, J = 14.3 Hz), 54.42 (t, J = 4.2 Hz), 52.62, 24.68.

^{19}F NMR (471 MHz, CDCl$_3$) δ -139.27 (m, 2F), -159.39 (d, J = 15.9 Hz, 2F).

HRMS (ESI-TOF) m/z: [M - H]$^-$ Calculated for [$C_{17}H_{11}O_2N_2F_4$]$^-$: 351.0762, found: 351.0764

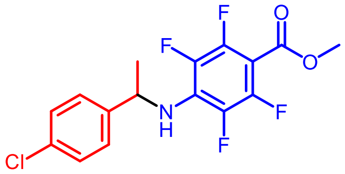

12

Methyl-4-((1-(4-chlorophenyl)ethyl)amino)-2,3,5,6-tetrafluorobenzoate

Compound 12 is prepared using both general procedures A and B above. Purified by flash chromatography (ethyl acetate/hexane 2-20%) to obtain the product as an off-white colored solid (10.6 mg, 50% for procedure A and 18.9 mg, 87% yield for procedure B).

^{1}H NMR (500 MHz, CDCl$_3$) δ 7.31 (m, 2H), 7.22 – 7.18 (m, 2H), 4.99 (p, J = 7.2 Hz, 1H), 4.44 – 4.37 (m, 1H), 3.88 (d, J = 1.1 Hz, 3H), 1.57 (d, J =6.8 Hz, 3H).

^{13}C NMR (126 MHz, CDCl$_3$) δ 160.98 (d, J = 3.2 Hz), 146.21 (dm, J = 260.8 Hz), 142.33, 142.33 (dm, J = 238.1 Hz), 133.37, 129.90 (t, J = 11.4 Hz), 129.05, 126.91, 99.35, 54.08 (t, J = 4.3 Hz), 52.54, 24.61.

^{19}F NMR (471 MHz, CDCl$_3$) δ -140.16 (m, 2F), -159.36 (d, J = 15.4 Hz, 2F).

HRMS (ESI-TOF) m/z: [M - H]$^-$ Calculated for [$C_{16}H_{11}O_2NClF_4$]$^-$: 360.0420, found: 360.0420

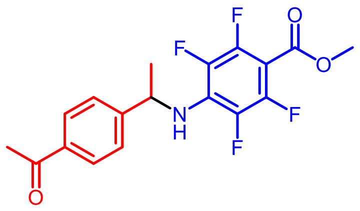

13

Methyl-4-((1-(4-acetylphenyl)ethyl)amino)-2,3,5,6-tetrafluorobenzoate
Compound 13 is prepared using both general procedures A and B above. Purified by flash chromatography (ethyl acetate/hexane 2-20%) to obtain the product as an off-white colored solid (17.1 mg, 77% for procedure A and 18.9 mg, 85% yield for procedure B).
^{1}H NMR (500 MHz, $CDCl_3$) δ 7.95 – 7.89 (m, 2H), 7.39 – 7.33 (m, 2H), 5.07 (q, J = 7.3 Hz, 1H), 4.50 (s, 1H), 3.87 (s, 3H), 2.58 (s, 3H), 1.60 (d, J = 6.8 Hz, 3H).
^{13}C NMR (126 MHz, $CDCl_3$) δ 197.47, 160.93, 149.16, 146.61(dm, J = 289.8 Hz), 136.58, 138.38 (dm, J = 244.4 Hz), 129.82 (t, J = 11.5 Hz), 129.03, 125.68, 99.56 (t, J = 14.3 Hz), 54.47 (t, J = 4.3 Hz), 52.56, 26.59, 24.61.
^{19}F NMR (471 MHz, $CDCl_3$) δ -140.15 (m, 2F), -159.41 (m, 2F).
HRMS (ESI-TOF) m/z: $[M - H]^-$ Calculated for $[C_{18}H_{14}O_3NF_4]^-$: 368.0915, found: 368.0917

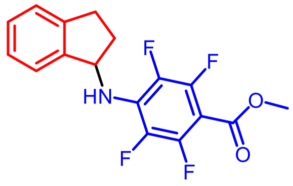

14

Methyl-4-((2,3-dihydro-1H-inden-1-yl)amino)-2,3,5,6-tetrafluorobenzoate
Compound 14 is prepared using both general procedures A and B above. Purified by flash chromatography (ethyl acetate/hexane 2-20%) to obtain the product as a yellowish solid (9.0 mg, 44% for procedure A and 14.2 mg, 70% yield for procedure B).
^{1}H NMR (500 MHz, $CDCl_3$) δ 7.36 (dd, J = 7.2, 1.2 Hz, 1H), 7.35-7.25 (m, 3H), 5.48 – 5.36 (m, 1H), 4.35 (s, 1H), 3.06 (ddd, J = 16.1, 8.6, 4.6 Hz, 1H), 2.91 (dt, J = 15.9, 7.7 Hz, 1H), 2.62 (dddd, J = 13.0, 8.2, 7.2, 4.6 Hz, 1H), 2.03 – 1.91 (m, 1H).
^{13}C NMR (126 MHz, $CDCl_3$) δ 161.20 (d, J = 3.1 Hz), 146.65 (dm, J = 264.7 Hz), 143.40, 143.11, 136.54 (dm, J = 258.4 Hz), 130.83 (d, J = 11.7 Hz), 128.64, 127.10, 125.12, 124.15, 98.52, 60.38 (t, J = 4.4 Hz), 52.52, 35.29, 30.01.
^{19}F NMR (471 MHz, $CDCl_3$) δ -140.25 (m, 2F), -160.31 (m, 2F).
HRMS (ESI-TOF) m/z: $[M - H]^-$ Calculated for $[C_{17}H_{12}O_2NF_4]^-$: 338.0810, found: 338.0814

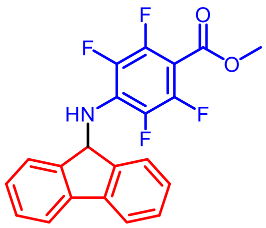

15

Methyl-4-((9H-fluoren-9-yl)amino)-2,3,5,6-tetrafluorobenzoate

Compound 15 is prepared using both general procedures A and B above. Purified by flash chromatography (ethyl acetate/hexane 2-20%) to obtain the product as a yellowish solid (11.6 mg, 50% for procedure A and 20.2 mg, 87% yield for procedure B).

^{1}H NMR (500 MHz, $CDCl_3$) δ 7.72 (d, J = 7.6 Hz, 2H), 7.53 (d, J = 7.5 Hz, 2H), 7.44 (t, J = 7.5 Hz, 2H), 7.35 – 7.29 (m, 2H), 5.98 (d, J = 9.8 Hz, 1H), 4.40 (d, J = 10.2 Hz, 1H), 3.95 (s, 3H).

^{13}C NMR (126 MHz, $CDCl_3$) δ 161.08 (t, J = 3.0 Hz), 146.57 (dm, J = 278.5 Hz), 144.36, 140.22, 136.96 (dm, J = 239.6 Hz), 131.11 (tt, J = 10.9, 3.8 Hz), 129.23, 128.06, 124.89, 120.24, 99.44 (t, J = 14.2 Hz), 60.05 (t, J = 5.1 Hz), 52.63.

^{19}F NMR (471 MHz, $CDCl_3$) δ -139.73 (m, 2F), -158.84 (m, 2F).

HRMS (ESI-TOF) m/z: $[M - H]^-$ Calculated for $[C_{21}H_{12}O_2NF_4]^-$: 386.0814, found: 386.0814

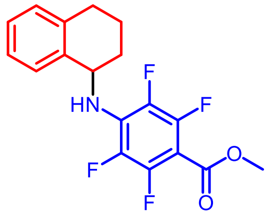

16

Methyl-2,3,5,6-tetrafluoro-4-((1,2,3,4-tetrahydronaphthalen-1-yl)amino)benzoate

Compound 16 is prepared using both general procedures A and B above. Purified by flash chromatography (ethyl acetate/hexane 2-20%) to obtain the product as an off-white colored liquid (9.0 mg, 42% for procedure A and 15.1 mg, 71% yield for procedure B).

^{1}H NMR (500 MHz, $CDCl_3$) δ 7.40 (dd, J = 7.2, 2.0 Hz, 1H), 7.28 – 7.17 (m, 2H), 7.15 (dd, J = 7.3, 1.8 Hz, 1H), 5.05 (dt, J = 9.6, 5.0 Hz, 1H), 4.36 (s, 1H), 3.93 (s, 3H), 2.87 (dt, J = 17.0, 5.7 Hz, 1H), 2.78 (ddd, J = 16.9, 8.3, 5.9 Hz, 1H), 2.06 (dddd, J = 13.2, 9.5, 4.8, 3.5 Hz, 1H), 1.97 – 1.77 (m, 1H).

^{13}C NMR (126 MHz, $CDCl_3$) δ 160.17, 145.62 (dm, J = 252.0 Hz), 136.45, 135 (dm, J = 239.0 Hz), 135.37, 129.44 (m), 128.39, 128.21, 126.87, 125.43, 97.67, 51.85 (t, J = 4.6 Hz), 51.48, 29.62, 28.12, 17.76.

^{19}F NMR (471 MHz, $CDCl_3$) δ -140.22 (m, 2F), -159.81 (m, 2F).

HRMS (ESI-TOF) m/z: $[M - H]^-$ Calculated for $[C_{18}H_{14}O_2NF_4]^-$: 352.0966, found: 352.0970

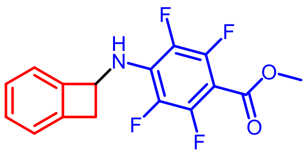

17

Methyl-4-(bicyclo[4.2.0]octa-1,3,5-trien-7-ylamino)-2,3,5,6-tetrafluorobenzoate

Compound 17 is prepared using both general procedures A and B above. Purified by flash chromatography (ethyl acetate/hexane 2-20%) to obtain the product as a yellowish liquid (3.9 mg, 20% for procedure A and 3.9 mg, 25% yield for procedure B).

^{1}H NMR (500 MHz, CDCl$_3$) δ 7.27 (d, J = 7.4 Hz, 1H), 7.22 (t, J = 7.4 Hz, 1H), 7.16 (t, J = 7.4 Hz, 1H), 7.11 (d, J = 7.2 Hz, 1H), 5.35 (dq, J = 7.8, 2.5 Hz, 1H), 4.69 (d, J = 7.9 Hz, 1H), 3.65 (s, 3H), 3.66 (dd, J = 14.3, 4.6 Hz, 1H), 3.04 – 2.98 (m, 1H).

^{13}C NMR (126 MHz, CDCl$_3$) δ 161.13, 146.59 (dm, J = 245.5 Hz), 145.05, 142.60, 136.55 (dm, J = 256.8 Hz), 129.81, 128.61, 127.75, 123.83, 122.30, 98.89, 55.41 (t, J = 4.7 Hz), 52.55, 41.63 (t, J = 1.6 Hz).

^{19}F NMR (471 MHz, CDCl$_3$) δ -140.21 (m, 2F), -161.09 (m, 2F)

Note HRMS could not be obtained for this compound

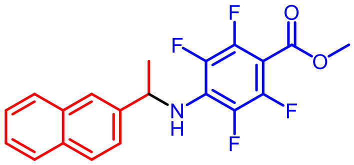

18

Methyl-2,3,5,6-tetrafluoro-4-((1-(naphthalen-2-yl)ethyl)amino)benzoate

Compound 18 is prepared using both general procedures A and B above. Purified by flash chromatography (ethyl acetate/hexane 2-20%) to obtain the product as a yellowish solid (16.3 mg, 72% for procedure A and 16.8 mg, 74% yield for procedure B).

^{1}H NMR (500 MHz, CDCl$_3$) δ 7.86 – 7.78 (m, 3H), 7.71 (d, J = 1.9 Hz, 1H), 7.52 – 7.43 (m, 2H), 7.40 (dd, J = 8.5, 1.9 Hz, 1H), 5.19 (q, J = 6.9 Hz, 1H), 4.56 (s, 1H), 3.87 (s, 3H), 1.67 (d, J = 6.7 Hz, 3H).

^{13}C NMR (126 MHz, CDCl$_3$) δ 161.03 (m), 146.49 (dm, J = 249.5 Hz), 141.13, 136.79 (dm, J = 249.5 Hz), 133.35, 132.90, 130.26 (ddt, J = 11.4, 7.5, 3.7 Hz), 128.88, 127.93, 127.70, 126.40, 126.07, 124.26, 123.61, 99.07, 54.75 (t, J = 4.3 Hz), 52.48, 24.70.

^{19}F NMR (471 MHz, CDCl$_3$) δ -140.28 (m, 2F), -159.34 (m, 2F).

HRMS (ESI-TOF) m/z: $[M - H]^-$ Calculated for $[C_{20}H_{14}O_2NF_4]^-$: 376.0966, found: 376.0971

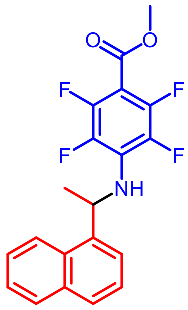

19

Methyl-2,3,5,6-tetrafluoro-4-((1-(naphthalen-1-yl)ethyl)amino)benzoate
Compound 19 is prepared using both general procedures A and B above. Purified by flash chromatography (ethyl acetate/hexane 2-20%) to obtain the product as a yellowish solid (14.7 mg, 65% for procedure A and 17.0 mg, 75% yield for procedure B).
^{1}H NMR (500 MHz, $CDCl_3$) δ 8.09 (d, J = 8.5 Hz, 1H), 7.92 – 7.88 (m, 1H), 7.79 (t, J = 4.7 Hz, 1H), 7.58 (ddd, J = 8.5, 6.8, 1.5 Hz, 1H), 7.55 – 7.50 (m, 1H), 7.44 (d, J = 4.9 Hz, 2H), 5.91 (p, J = 7.0 Hz, 1H), 4.60 (d, J = 8.0 Hz, 1H), 3.89 (s, 3H), 1.73 (d, J = 6.7 Hz, 3H).
^{13}C NMR (126 MHz, $CDCl_3$) δ 161.21 (m), 146.44 (dm, J = 247.0 Hz), 139.16, 136.64 (dm, J = 224.3 Hz), 134.11, 130.53, 130.17 (ddt, J = 11.2, 7.4, 4.0 Hz), 129.08, 128.30, 126.55, 125.86, 125.42, 122.48, 121.50, 98.90 (t, J = 14.2 Hz), 52.47, 50.49 (t, J = 4.5 Hz), 23.67.
^{19}F NMR (471 MHz, $CDCl_3$) δ -140.18 (m, 2F), -160.11 (m, 2F).
HRMS (ESI-TOF) m/z: $[M - H]^-$ Calculated for $[C_{20}H_{14}O_2NF_4]^-$: 376.0966, found: 376.0969

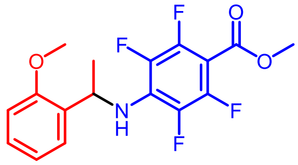

20

Methyl-2,3,5,6-tetrafluoro-4-((1-(2-methoxyphenyl)ethyl)amino)benzoate
Compound 20 is prepared using both general procedures A and B above. Purified by flash chromatography (ethyl acetate/hexane 2-20%) to obtain the product as a yellowish solid (14.6 mg, 68% for procedure A and 15.4 mg, 72% yield for procedure B).
^{1}H NMR (500 MHz, $CDCl_3$) δ 7.28 (ddd, J = 15.7, 7.6, 1.6 Hz, 1H), 7.17 (d, J = 7.2 Hz, 1H), 6.93 – 6.89 (m, 2H), 5.08 (p, J = 7.3 Hz, 1H), 4.97 (d, J = 8.9 Hz, 1H), 3.80 (s, J = 1.5 Hz, 3H), 3.79 (s, J = 1.5 Hz, 3H), 1.51 (d, J = 6.6 Hz, 3H).
^{13}C NMR (126 MHz, $CDCl_3$) δ 161.22, 156.88, 146.41 (dm, J = 248.4 Hz), 136.96 (dm, J = 242.0 Hz), 131.16, 130.94, 128.67, 127.32, 120.81, 111.09, 98.58 (t, J = 14.1 Hz), 55.27, 52.89 (t, J = 4.8 Hz), 52.42, 22.43.
^{19}F NMR (471 MHz, $CDCl_3$) δ -140.80 (m, 2F), -160.08 (m, 2F).
HRMS (ESI-TOF) m/z: $[M - H]^-$ Calculated for $[C_{17}H_{14}O_3NF_4]^-$: 356.0915, found: 356.0919

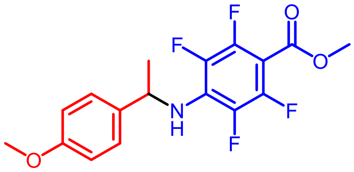

21

Methyl-2,3,5,6-tetrafluoro-4-((1-(4-methoxyphenyl)ethyl)amino)benzoate

Compound 21 is prepared using both general procedures A and B above. Purified by flash chromatography (ethyl acetate/hexane 2-20%) to obtain the product as a yellowish liquid (17.1 mg, 80% for procedure A and 15.0 mg, 70% yield for procedure B).

^{1}H NMR (500 MHz, CDCl$_3$) δ 7.23 – 7.16 (m, 2H), 6.89 – 6.82 (m, 2H), 5.01 – 4.94 (m, 1H), 4.39 (s, 1H), 3.88 (s, 3H), 3.78 (s, 3H), 1.56 (d, *J* = 6.7 Hz, 3H).

^{13}C NMR (126 MHz, CDCl$_3$) δ 161.11, 159.02, 146.64 (dm, *J* = 258.0 Hz), 137.02 (dm, 235.6), 135.78, 130.35 (t, *J* = 11.5 Hz), 126.73, 114.20, 99.03 (t, *J* = 14.2 Hz), 55.27, 54.05 (t, *J* = 4.3 Hz), 52.49, 24.44.

^{19}F NMR (471 MHz, CDCl$_3$) δ -140.45 (m, 2F), -159.42 (m, 2F).

HRMS (ESI-TOF) m/z: [M - H]$^-$ Calculated for [$C_{17}H_{14}O_3NF_4$]$^-$: 356.0915, found: 356.0920

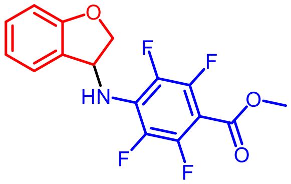

22

Methyl-4-((2,3-dihydrobenzofuran-3-yl)amino)-2,3,5,6-tetrafluorobenzoate

Compound 22 is prepared using both general procedures A and B above. Purified by flash chromatography (ethyl acetate/hexane 2-20%) to obtain the product as a yellowish liquid (14.0 mg, 68% for procedure A and 11.7 mg, 57% yield for procedure B).

^{1}H NMR (500 MHz, CDCl$_3$) δ 7.37 (d, *J* = 7.5 Hz, 1H), 7.34 – 7.28 (m, 1H), 6.98 (td, *J* = 7.5, 1.0 Hz, 1H), 6.92 (d, *J* = 8.1 Hz, 1H), 5.52 (tq, *J* = 6.6, 3.1 Hz, 1H), 4.67 (dd, *J* = 10.3, 7.1 Hz, 1H), 4.52 (ddt, *J* = 10.2, 3.0, 1.5 Hz, 1H), 4.39 – 4.33 (m, 1H), 3.93 (s, 3H).

^{13}C NMR (126 MHz, CDCl$_3$) δ 160.94, 160.25, 146.57 (dm, *J* = 242.8 Hz), 136.50 (dm, *J* = 249.9 Hz), 131.08, 129.40, 126.19, 125.27, 121.46, 110.80, 99.44 (d, *J* = 14.2 Hz), 78.06 (t, *J* = 2.7 Hz), 57.14 (t, *J* = 4.2 Hz), 52.63.

^{19}F NMR (471 MHz, CDCl$_3$) δ -139.69 (m, 2F), -160.21 (m, 2F).

HRMS (ESI-TOF) m/z: [M - H]$^-$ Calculated for [$C_{16}H_{10}O_3NF_4$]$^-$: 342.0398, found: 342.0398

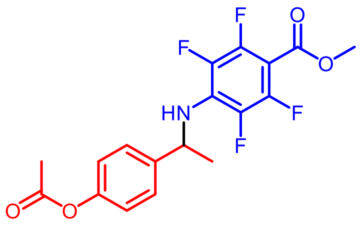

23

Methyl-4-((1-(4-acetoxyphenyl)ethyl)amino)-2,3,5,6-tetrafluorobenzoate

Compound 23 is prepared using both general procedures A and B above. Purified by flash chromatography (ethyl acetate/hexane 2-20%) to obtain the product as a yellowish solid (16.2 mg, 70% for procedure A and 18.0 mg, 78% yield for procedure B). Compound 23 co-elutes with the corresponding aniline of Fluoro-N_3. Yields account for this impurity by estimating the molar ratio by ^{19}F NMR.

^{1}H NMR (500 MHz, $CDCl_3$) δ 7.30 – 7.26 (m, 2H), 7.08 – 7.03 (m, 2H), 5.02 (dd, *J* = 10.3, 4.2 Hz, 1H), 4.44 (d, *J* = 8.5 Hz, 1H), 3.88 (s, 3H), 2.28 (s, 3H), 1.56 (d, *J* = 6.7 Hz, 3H).

^{13}C NMR (126 MHz, $CDCl_3$) δ 169.41, 161.06 (m), 150.00, 146.35 (dm, *J* = 254.5 Hz), 141.23, 136.62 (dm, *J* = 238.1 Hz), 130.23 – 129.84 (m), 126.65, 121.93, 99.19 (t, *J* = 14.2 Hz), 54.05 (t, *J* = 4.4 Hz), 52.50, 24.48, 21.10.

^{19}F NMR (471 MHz, $CDCl_3$) δ -140.26 (m, 2F), -159.47 (m, 2F).

HRMS (ESI-TOF) m/z: $[M - H]^-$ Calculated for $[C_{18}H_{14}O_4NF_4]^-$: 384.0864, found: 384.0866

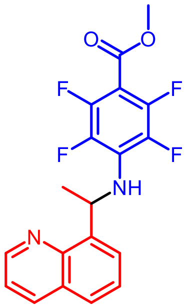

24

Methyl-2,3,5,6-tetrafluoro-4-((1-(quinolin-8-yl)ethyl)amino)benzoate

Compound 24 is prepared using both general procedures A and B above. Purified by flash chromatography (ethyl acetate/hexane 2-20%) to obtain the product as a yellowish solid (11.3 mg, 50% for procedure A and 10.7 mg, 47% yield for procedure B).

^{1}H NMR (500 MHz, $CDCl_3$) δ 8.92 (dd, *J* = 4.2, 1.8 Hz, 1H), 8.18 (dd, *J* = 8.3, 1.8 Hz, 1H), 7.73 (dd, *J* = 8.2, 1.4 Hz, 1H), 7.57 (dd, *J* = 7.1, 1.5 Hz, 1H), 7.50 – 7.41 (m, 2H), 6.80 (s, 1H), 5.65 (q, *J* = 6.9 Hz, 1H), 3.86 (s, 3H), 1.83 (d, *J* = 6.8 Hz, 3H).

^{13}C NMR (126 MHz, $CDCl_3$) δ 161.32 (m), 148.96, 146.09 (dm, *J* = 247.0 Hz), 145.80, 140.47, 136.89, 136.57 (dm, *J* = 236.9 Hz), 131.24 (t, *J* = 11.3 Hz), 129.06, 127.73, 127.56, 126.35, 121.15, 98.00 (t, *J* = 13.9 Hz), 55.44 (t, *J* = 5.1 Hz), 52.36, 24.05.

^{19}F NMR (471 MHz, $CDCl_3$) δ -140.97 (m, 2F), -159.90 (m, 2F).

HRMS (ESI-TOF) m/z: $[M - H]^-$ Calculated for $[C_{19}H_{13}O_2N_2F_4]^-$: 377.0919, found: 377.0923

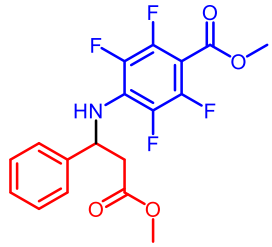

25

Methyl-2,3,5,6-tetrafluoro-4-((3-methoxy-3-oxo-1-phenylpropyl)amino)benzoate
Compound 25 is prepared using both general procedures A and B above. Purified by flash chromatography (ethyl acetate/hexane 2-20%) to obtain the product as a yellowish liquid (11.6 mg, 50% for procedure A and 19.2 mg, 83% yield for procedure B).
^{1}H NMR (500 MHz, $CDCl_3$) δ 7.25 – 7.22 (m, 2H), 7.19 (dd, *J* = 7.3, 3.0 Hz, 3H), 5.23 (d, *J* = 3.8 Hz, 1H), 3.79 (s, 3H), 3.58 (s, 3H), 2.95 – 2.68 (m, 2H).
^{13}C NMR (126 MHz, $CDCl_3$) δ 171.04, 160.98 (t, *J* = 3.0 Hz), 146.33 (dm, *J* = 254.5 Hz), 140.89, 137.02 (dm, *J* = 244.4 Hz), 129.93 (m), 128.98, 128.10, 125.80, 99.71 (t, *J* = 14.2 Hz), 55.70 (t, *J* = 4.8 Hz), 52.54, 52.04, 42.11.
^{19}F NMR (471 MHz, $CDCl_3$) δ -140.21 (m, 2F), -158.89 (m, 2F)
HRMS (ESI-TOF) m/z: $[M - H]^-$ Calculated for $[C_{18}H_{14}O_4NF_4]^-$: 384.0864, found: 384.0867

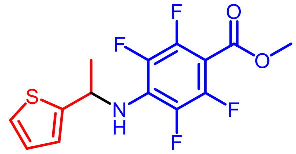

26

Methyl-2,3,5,6-tetrafluoro-4-((1-(thiophen-2-yl)ethyl)amino)benzoate
Compound 26 is prepared using both general procedures A and B above. Purified by flash chromatography (ethyl acetate/hexane 2-20%) to obtain the product as a yellowish liquid (14.0 mg, 70% for procedure A and 13.6 mg, 68% yield for procedure B).
^{1}H NMR (500 MHz, $CDCl_3$) δ 7.21 (dd, *J* = 5.0, 1.3 Hz, 1H), 6.96 (d, *J* = 3.5 Hz, 1H), 6.93 (dd, *J* = 5.0, 3.6 Hz, 1H), 5.38 – 5.22 (m, 1H), 4.36 (d, *J* = 9.4 Hz, 1H), 3.90 (s, 3H), 1.69 (d, *J* = 6.7 Hz, 3H).
^{13}C NMR (126 MHz, $CDCl_3$) δ 161.02 (m), 147.54, 147.15 (dm, *J* = 259.6 Hz), 136.42 (dm, *J* = 240.7 Hz), 129.87 (m), 126.88, 124.61, 123.93, 99.71 (t, *J* = 14.2 Hz), 52.56, 50.50 (t, *J* = 4.9 Hz), 24.33.
^{19}F NMR (471 MHz, $CDCl_3$) δ -140.19 (m, 2F), -158.77 (m, 2F).
HRMS (ESI-TOF) m/z: $[M - H]^-$ Calculated for $[C_{14}H_{10}O_2NF_4S]^-$: 332.0374, found: 332.0378

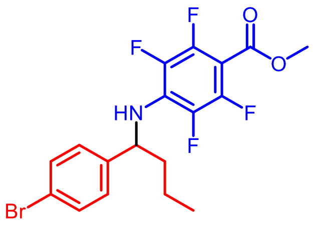

27

Methyl-4-((1-(4-bromophenyl)butyl)amino)-2,3,5,6-tetrafluorobenzoate
Compound 27 is prepared using both general procedures A and B above. Purified by flash chromatography (ethyl acetate/hexane 2-20%) to obtain the product as a yellowish liquid (23.0 mg, 88% for procedure A and 22.1 mg, 85% yield for procedure B).
^{1}H NMR (500 MHz, CDCl$_3$) δ 7.36 (d, *J* = 8.3 Hz, 2H), 7.03 (d, *J* = 8.2 Hz, 2H), 4.69 (q, *J* = 6.9 Hz, 1H), 4.35 (s, 1H), 3.67 (s, 3H) 1.83 – 1.72 (m, 1H), 1.65 (ddt, *J* = 13.3, 9.8, 6.4 Hz, 1H), 1.36 (dddd, *J* = 12.4, 9.9, 7.8, 4.3 Hz, 1H), 1.30 – 1.20 (m, 1H), 0.87 (t, *J* = 7.4 Hz, 3H).
13C NMR (126 MHz, CDCl$_3$) δ 161.10 (m), 146.36 (dm, *J* = 254.5 Hz), 141.95, 136.66 (dm, *J* = 243.2 Hz), 131.91, 130.16 (ddd, *J* = 11.4, 7.6, 3.8 Hz), 127.74, 121.41, 99.48, 58.59 (t, *J* = 4.1 Hz), 52.52, 40.76, 19.49, 13.68.
^{19}F NMR (471 MHz, CDCl$_3$) δ -140.19 (m, 2F), -159.25 (m, 2F).
HRMS (ESI-TOF) m/z: [M - H]$^-$ Calculated for [$C_{18}H_{15}O_2NF_4Br$]$^-$: 432.0233, found: 432.0231

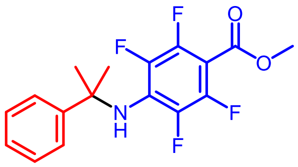

28

Methyl-2,3,5,6-tetrafluoro-4-((2-phenylpropan-2-yl)amino)benzoate
Compound 28 is prepared using both general procedures A and B above. Purified by flash chromatography (ethyl acetate/hexane 2-20%) to obtain the product as a yellowish liquid (13.9 mg, 68% for procedure A and 18.4 mg, 90% yield for procedure B).
^{1}H NMR (500 MHz, CDCl$_3$) δ 7.41 – 7.37 (m, 2H), 7.32 (dd, *J* = 8.5, 6.9 Hz, 2H), 7.25 – 7.21 (m, 1H), 4.71 (s, 1H), 3.88 (s, 3H), 1.73 (s, 6H).
^{13}C NMR (126 MHz, CDCl$_3$) δ 161.02 (m), 146.90 (t, *J* = 2.3 Hz), 146.85 (dm, *J* = 253.3 Hz), 136.36 (dm, *J* = 241.9 Hz), 129.39 (m), 128.42, 126.71, 124.60 (d, *J* = 1.3 Hz), 98.97 (t, *J* = 14.3 Hz), 57.99, 52.49, 31.99 (t, *J* = 2.5 Hz).
^{19}F NMR (471 MHz, CDCl$_3$) δ -140.33 (m, 2F), -156.00 (m, 2F).
HRMS (ESI-TOF) m/z: [M - H]$^-$ Calculated for [$C_{17}H_{14}O_2NF_4$]$^-$: 340.0966, found: 340.0967

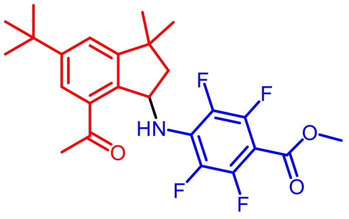

29

Methyl-4-((7-acetyl-5-(tert-butyl)-3,3-dimethyl-2,3-dihydro-1H-inden-1-yl)amino)-2,3,5,6-tetrafluorobenzoate

Compound 29 is prepared using both general procedures A and B above. Purified by flash chromatography (ethyl acetate/hexane 2-20%) to obtain the product as a yellowish liquid (18.1 mg, 65% for procedure A and 22.9 mg, 82% yield for procedure B).

^{1}H NMR (500 MHz, CDCl$_3$) δ 7.76 (d, *J* = 1.8 Hz, 1H), 7.44 (d, *J* = 1.8 Hz, 1H), 5.66 (dq, *J* = 7.3, 2.3 Hz, 1H), 4.23 (s, 1H), 3.91 (s, 3H), 2.60 (s, 3H), 2.33 (dd, *J* = 13.7, 7.3 Hz, 1H), 2.22 – 2.15 (m, 1H), 1.39 (s, 9H), 1.35 (d, *J* = 12.5 Hz, 6H).

^{13}C NMR (126 MHz, CDCl$_3$) δ 199.75, 161.35 (m), 155.05, 153.49, 146.63 (dm, *J* = 277.2 Hz), 137.90, 136.65 (dm, *J* = 238.14 Hz), 134.40, 131.64 (m), 126.06, 123.94, 98.13 (t, *J* = 13.9 Hz), 58.61 (t, *J* = 3.9 Hz), 52.39, 49.85 (d, *J* = 1.7 Hz), 42.65, 35.05, 31.88, 31.39, 29.46, 28.28.

^{19}F NMR (471 MHz, CDCl$_3$) δ -141.29 (m, 2F), -161.26 (m, 2F).

HRMS (ESI-TOF) m/z: [M - H]$^-$ Calculated for [$C_{25}H_{26}O_3NF_4$]$^-$: 464.1854, found: 464.185

5.0 Catalyst Characterization

5.1 PXRD characterization

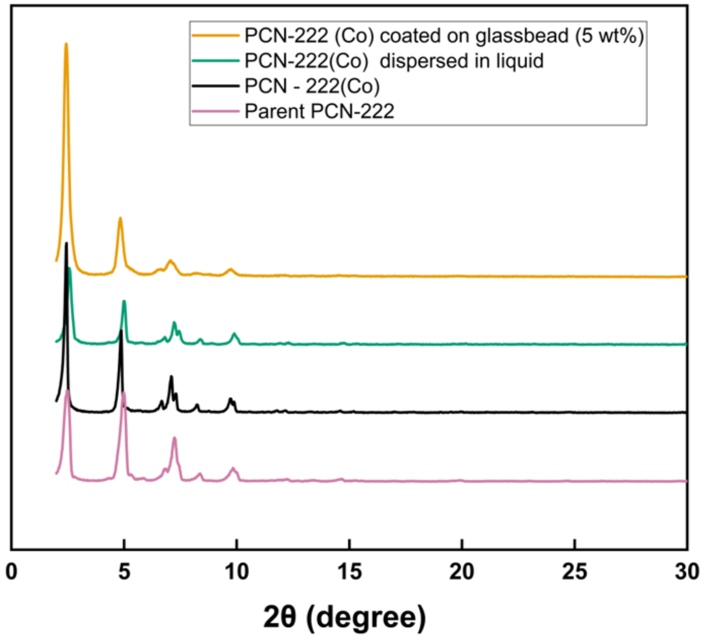

***Figure S15**: Powder X-Ray Diffraction (PXRD)*

5.2 Isotherm characterization

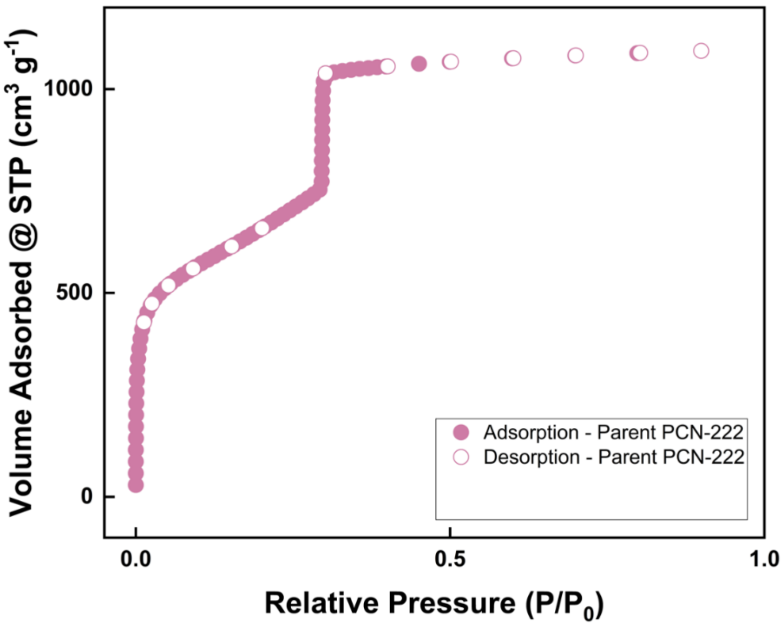

Figure S16: *N_2 isotherm of PCN-222 at 77K, Surface area = 2341 m^2 / g^{-1}*

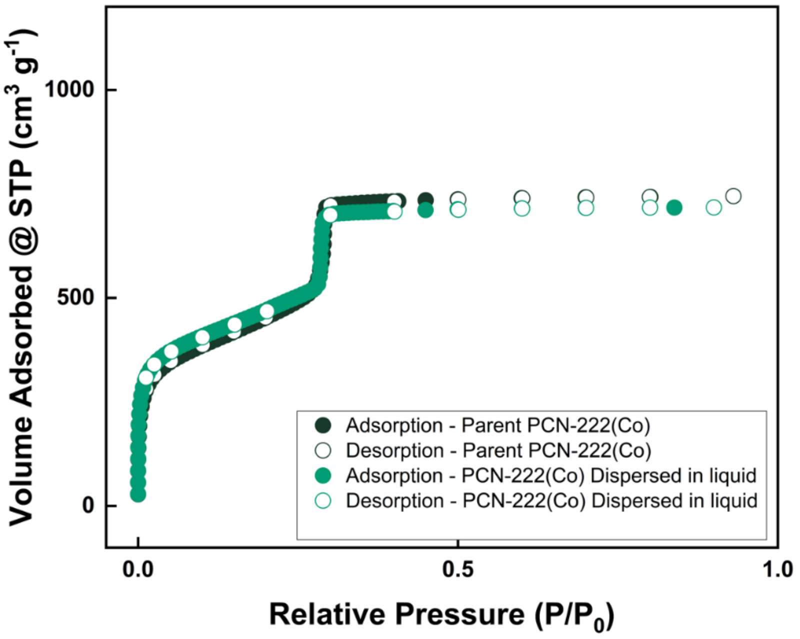

Figure S17: *N_2 Isotherm of PCN-222(Co) at 77K, Surface areas: parent PCN-222(Co) = 1642 m^2 / g, PCN-222(Co) dispersed in liquid = 1606 m^2 / g^{-1}*

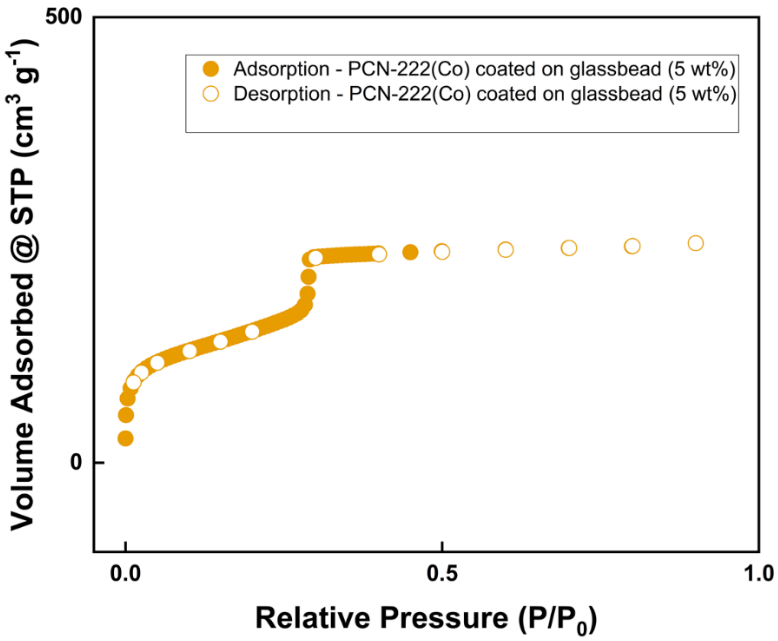

Figure S18: *N_2 isotherm PCN-222(Co) dispersed on glass beads (MOF) at 77K. Surface area =549 m^2 / g^{-1}*

5.3 SEM-EDS

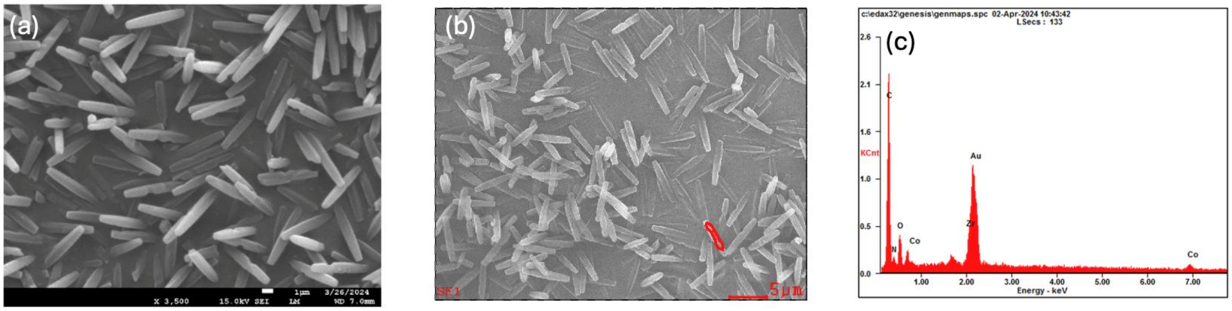

Figure S19: *(a) SEM of PCN-222 (b) SEM of PCN-222(Co) (c) EDS of PCN-222(Co) an average Atom ratio of 2.6 to 1 (Zr to Co) was determined which is close to the expected ratio of 3 to 1 (Zr to Co) if all the porphyrins linkers are metalated with Co.*

PCN-222(Co) Catalyst loading
The catalyst loading reported in this work is based-on complete metalation porphyrin linkers which is the maximum loading possible which is consistent with SEM/EDS characterization.

5.4 Post-Catalysis Catalyst Characterization

We characterized our PCN-222(Co) catalyst after catalysis to observe possible structural changes that may have occurred during reaction. Post-catalysis PXRD of PCN-222(Co) suggests that it retains crystallinity after catalysis (Figure S20). EDS characterization (Figure S22 and S23) also shows no reasonable difference in the atom ratio of Zr to Co (~ 3 to 1). SEM images, however, show some polydispersity in particle size in the post-catalysis PCN-222(Co). The pre-catalysis PCN-222(Co) had a more uniform particle size (Figure S21).

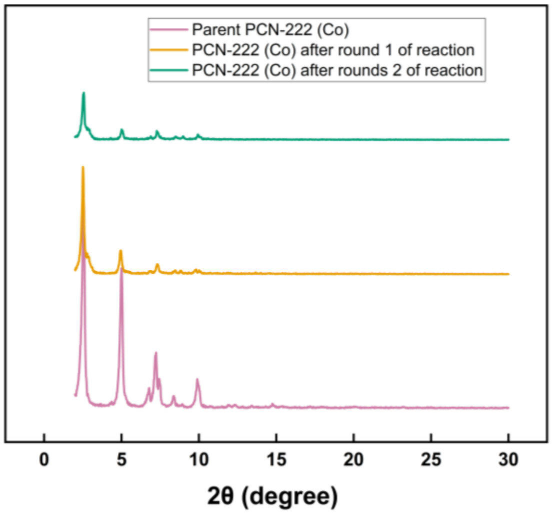

Figure S20: *PXRD of PCN-222(Co) before and after catalysis*

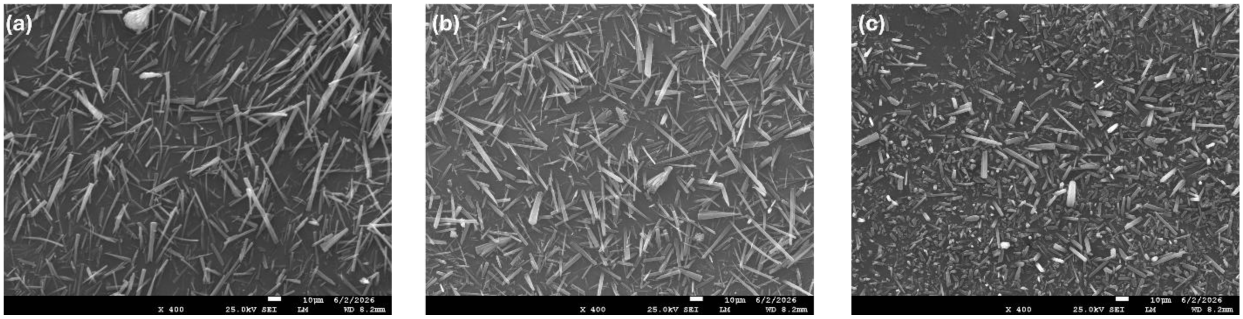

Figure S21: *SEM images (a) Parent PCN-222 (b) pre-catalysis PCN-222(Co) (c)Post-catalysis PCN-222(Co)*

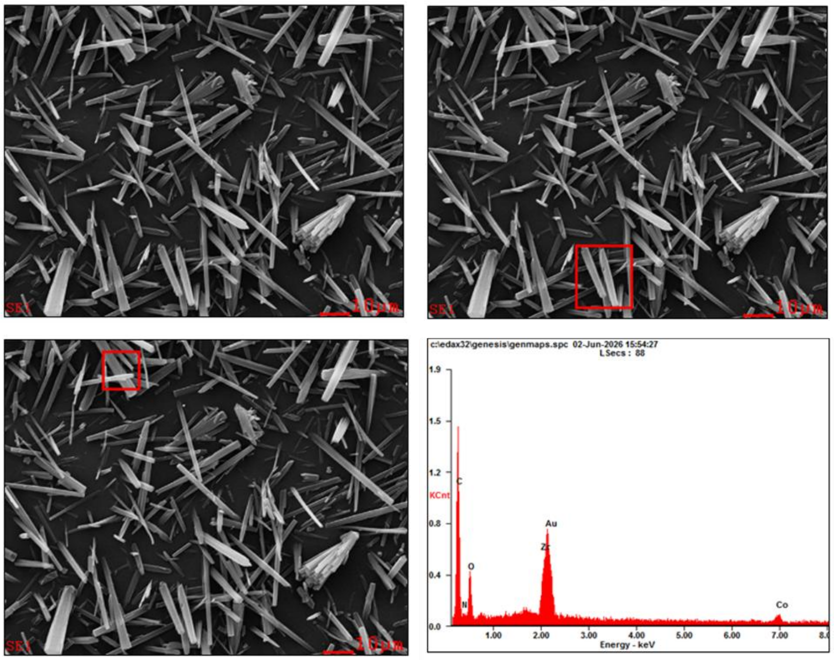

Figure S22: *PCN-222(Co) before catalysis, showing regions where elemental measurements were carried out. Average atom ratio of Zr to Co = 3 to 1.*

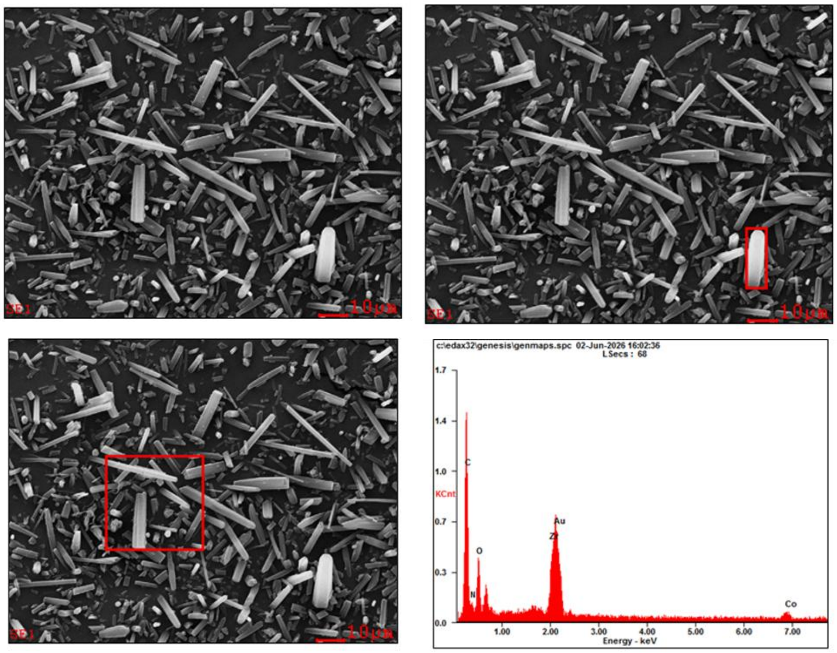

Figure S23: *PCN-222(Co) after catalysis, showing regions where elemental measurements were carried out. Average atom ratio of Zr to Co = 2.7 to 1.*

Note: There is batch to batch variation in the atom ratio of Zr to Co. However, we observe consistent ~3 to 1 ratio.

6.0 Filtration and Recycling Experiments

Filtration Experiment
To ensure catalysis is promoted by the MOF, and not any leached catalysts from the PCN-222 framework, a split filtration experiment (Figure S24) was carried out. Reactions were set up using procedure B (10 equiv C-H) in section 4.3.1 above with some modification. Using a 24-well aluminum reaction block, eleven 4 mL scintillation vials were charged with 3×5 mm PTFE magnetic stir bars, PCN-222(Co) catalyst (5 mol%, 0.003 mmol, 3.7 mg), ethylbenzene (0.6 mmol, 63.7 mg, 10 equiv), PhF (0.2 mL), and Fluoro-N_3 (14.9 mg, 0.06 mmol in 0.1 mL of PhF). The vials are capped, and the reaction mixture is allowed to react for 30 minutes (50 ºC, 350 rpm). After 30 minutes, six of the vials were filtered (filtered in S24) using a 200 nm PVDF syringe filter. The filtrates were then allowed to continue reacting alongside the other set of five vials that were not filtered (unfiltered in S24) at 50 ºC. At each desired time point, two vials (one filtered, one unfiltered) are removed from heating and allowed to cool to room temperature; the unfiltered reaction is then filtered using a 200 nm PVDF syringe filter. Internal standards are then added to both reactions and characterized by NMR. Product yield is obtained by ^{19}F NMR using 4,4-difluorobiphenyl as internal standard *Molecular sieve was not used in these reactions.*

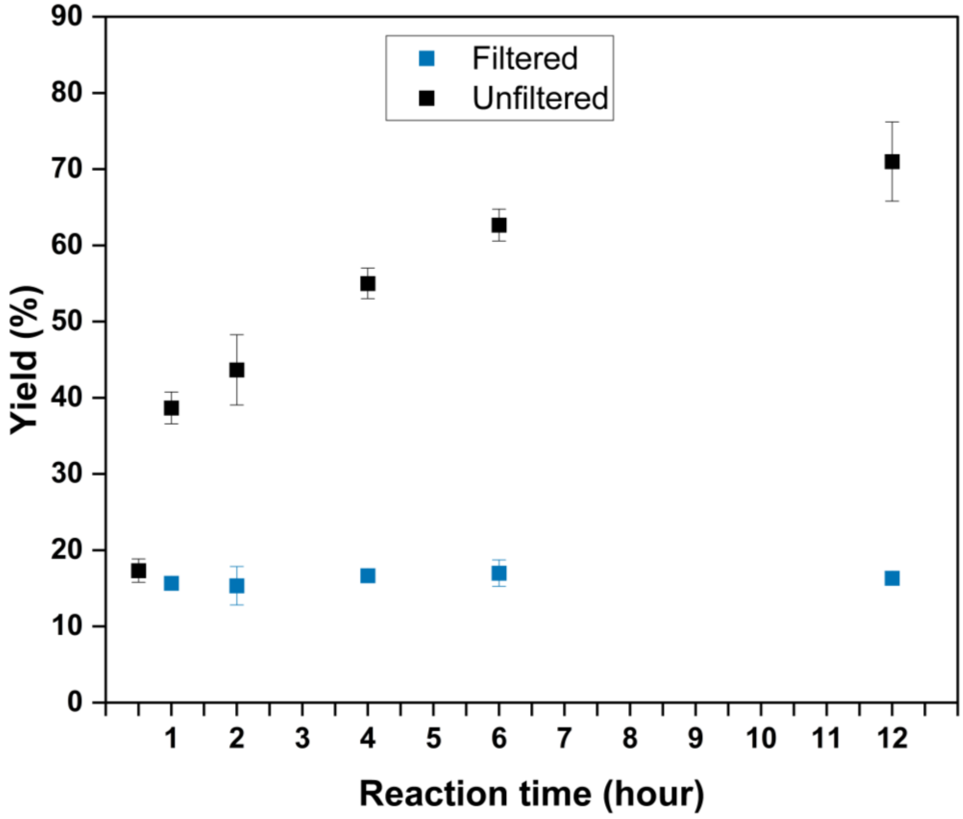

Figure S24: *Plot of Filtration experiment, showing that reaction does not proceed when MOF is filtered from the reaction mixture. Catalysis was done under air and molecular sieve was not used*

Catalyst Recycling Experiment

To test the recyclability of our PCN-222(Co), a recycling experiment was carried out (figure S25) Reactions were set up using procedure B (10 equiv C–H) in section 4.3.1 above with some modification. A 4 mL scintillation vial was charged with a 3×5 mm PTFE magnetic stir bars, PCN-222(Co) catalyst (5 mol%, 0.003 mmol, 3.7 mg), ethylbenzene (0.6 mmol, 63.7 mg, 10 equiv), PhF (0.2 mL), and Fluoro-N_3 (14.9 mg, 0.06 mmol in 0.1 mL of PhF). The vial is capped, and the reaction mixture is allowed to react (50 ºC, 350 rpm, 12 h). After 12 hours, the reaction mixture is diluted with 1 mL of PhF, vortex mixed and centrifuged (3000 rpm, 5 mins). After centrifugation the supernatant was pipetted into a new 4 mL vial and fresh 1 mL of PhF benzene is added, vortexed mixed and centrifuged to collect the supernatant once again. Next, the process is repeated twice with DCM. The combined reaction supernatants is concentrated under reduced pressure in a vacuum oven, then internal standard is added and yield is characterized by ^{19}F NMR. The PCN-222(Co) pallet in the initial reaction vial is also activated (5 h) to remove leftover solvent in the vacuum oven. The same vial is reused for the next reaction (run 2). The recycle sequence was repeated and we did 3 runs of catalysis with the same starting PCN-222(Co) catalyst. Consistent decrease (figure S25) in product yield was observed in more runs. This may be due to collapse of MOF structure which enables reaction under air

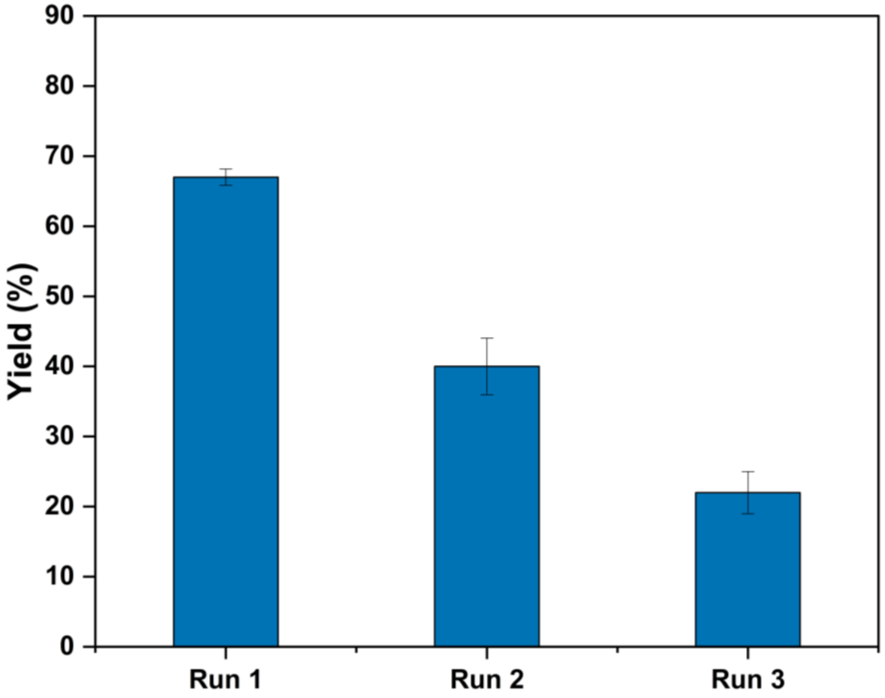

***Figure S25**: Recycling experiment showing how the activity of PCN-222(Co) decreases after reaction. Catalysis was done under air and molecular sieve was not used in these reactions.*

7.0 References

(1) Sugimoto, H.; Kuroda, K. The Cobalt Porphyrin−Lewis Base System: A Highly Selective Catalyst for Alternating Copolymerization of CO2 and Epoxide under Mild Conditions. Macromolecules **2008**, *41*, 312

(2) Thommes, M.; Kaneko, K.; Neimark, A. V.; Olivier, J. P.; Rodriguez-Reinoso, F.; Rouquerol, J.; Sing, K. S. W. Physisorption of Gases, with Special Reference to the Evaluation of Surface Area and Pore Size Distribution (IUPAC Technical Report). Pure Appl. Chem. **2015**, 87 (9–10), 1051–1069. https://doi.org/10.1515/pac-2014-1117.

(3) Liu, Y.; Moon, S.-Y.; Hupp, J. T.; Farha, O. K. Dual-Function Metal–Organic Framework as a Versatile Catalyst for Detoxifying Chemical Warfare Agent Simulants. ACS Nano **2015**, 9 (12), 12358–12364. https://doi.org/10.1021/acsnano.5b05660.

(4) Zhang, Y.; Ge, X.; Lu, H.; Li, G. Catalytic Decarboxylative C−N Formation to Generate Alkyl, Alkenyl, and Aryl Amines. Angew. Chem. Int. Ed. **2021**, 60 (4), 1845–1852. https://doi.org/10.1002/anie.202010974.

(5) Hwang, Y.; Baek, S. B.; Kim, D.; Chang, S. Chain Walking as a Strategy for Iridium-Catalyzed Migratory Amidation of Alkenyl Alcohols to Access α-Amino Ketones. J. Am. Chem. Soc. **2022**, 144 (9), 4277–4285. https://doi.org/10.1021/jacs.2c00948.

(6) Souza, V. V.; Accioly Jr, M.; Raminelli, C. White Light-Induced Slow Kinetics Generation of Arynes. Eur. J. Org. Chem. **2025**, 28 (36), e202500589. https://doi.org/10.1002/ejoc.202500589.

(7) Deng, T.; Mazumdar, W.; Yoshinaga, Y.; Patel, P. B.; Malo, D.; Malo, T.; Wink, D. J.; Driver, T. G. Rh2(II)-Catalyzed Intermolecular N-Aryl Aziridination of Olefins Using Nonactivated N Atom Precursors. J. Am. Chem. Soc. **2021**, 143 (45), 19149–19159. https://doi.org/10.1021/jacs.1c09229.

(8) Chehade, K. A. H.; Spielmann, H. P. Facile and Efficient Synthesis of 4-Azidotetrafluoroaniline: A New Photoaffinity Reagent. J. Org. Chem. **2000**, 65 (16), 4949–4953. https://doi.org/10.1021/jo000402p.

(9) Shi, M.; Ye, N.; Chen, W.; Wang, H.; Cheung, C.; Parmentier, M.; Gallou, F.; Wu, B. Simple Synthesis of Amides via Their Acid Chlorides in Aqueous TPGS-750-M. Org. Process Res. Dev. **2020**, 24 (8), 1543–1548. https://doi.org/10.1021/acs.oprd.0c00303.

(10) Ankner, T.; Hilmersson, G. Instantaneous Deprotection of Tosylamides and Esters with SmI2/Amine/Water. Org. Lett. **2009**, 11 (3), 503–506. https://doi.org/10.1021/ol802243d.

(11) Huang, X.; Webster, R. D.; Harms, K.; Meggers, E. Asymmetric Catalysis with Organic Azides and Diazo Compounds Initiated by Photoinduced Electron Transfer. J. Am. Chem. Soc. **2016**, 138 (38), 12636–12642. https://doi.org/10.1021/jacs.6b07692.

(12) Li, S.; Izang, J. C.; Duque, J. B.; Leonard, G. D.; Bour, J. R. High-Throughput Microscale MOF Catalysis via Solid Dispersion Reactor Dosing. Inorg. Chem. **2026**, 65 (13), 7368–7378. https://doi.org/10.1021/acs.inorgchem.6c00374.

(13) Palomba, J. M.; Harvey, S. P.; Kalaj, M.; Pimentel, B. R.; DeCoste, J. B.; Peterson, G. W.; Cohen, S. M. High-Throughput Screening of MOFs for Breakdown of V-Series Nerve Agents. ACS Appl. Mater. Interfaces **2020**, 12 (13), 14672–14677. https://doi.org/10.1021/acsami.9b21693.

(14) Stevens, R.; Palmer, H. E. P.; Miah, A. H.; Burley, G. A. Factors to Consider for Synthesis in 1536-Well Plates─An Amide Coupling Case Study for PROTAC Synthesis. J. Org. Chem. **2025**, 90 (6), 2192–2200. https://doi.org/10.1021/acs.joc.4c02456.
(15) Kuijpers, P. F.; Tiekink, M. J.; Breukelaar, W. B.; Broere, D. L. J.; van Leest, N. P.; van der Vlugt, J. I.; Reek, J. N. H.; de Bruin, B. Cobalt-Porphyrin-Catalysed Intramolecular Ring-Closing C−H Amination of Aliphatic Azides: A Nitrene-Radical Approach to Saturated Heterocycles. Chem. – Eur. J. **2017**, 23 (33), 7945–7952. https://doi.org/10.1002/chem.201700358.

8.0 NMR spectra of all compounds

NMR Spectra

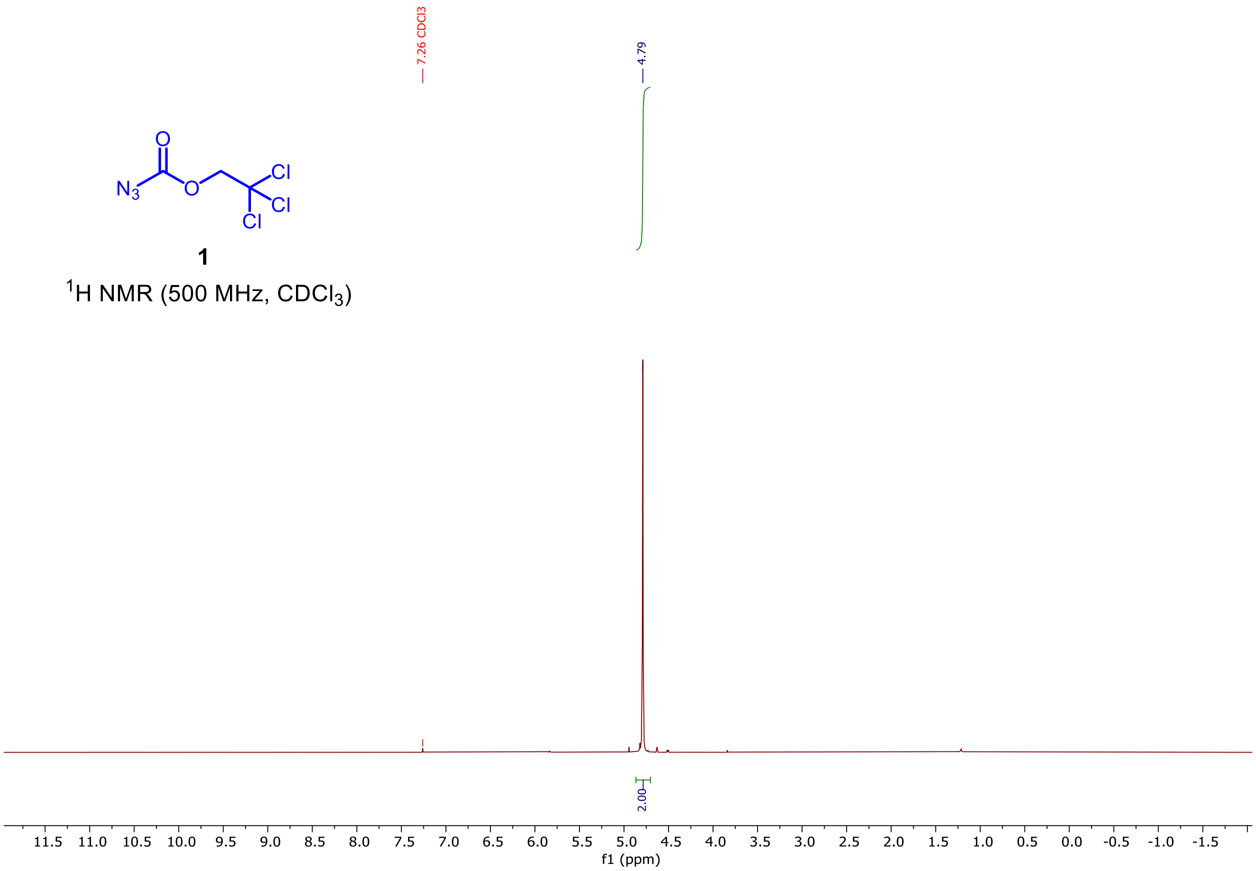

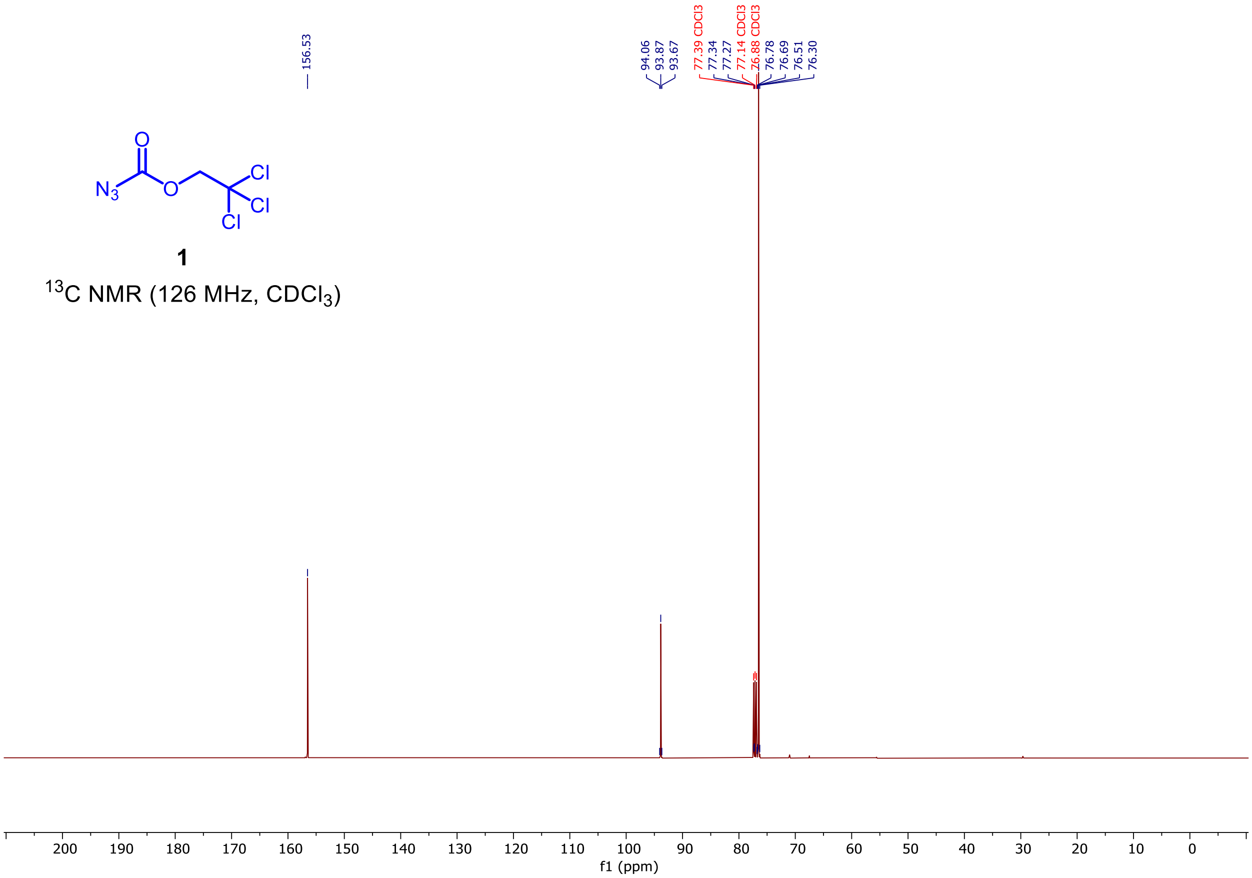

1

^{13}C NMR (126 MHz, $CDCl_3$)

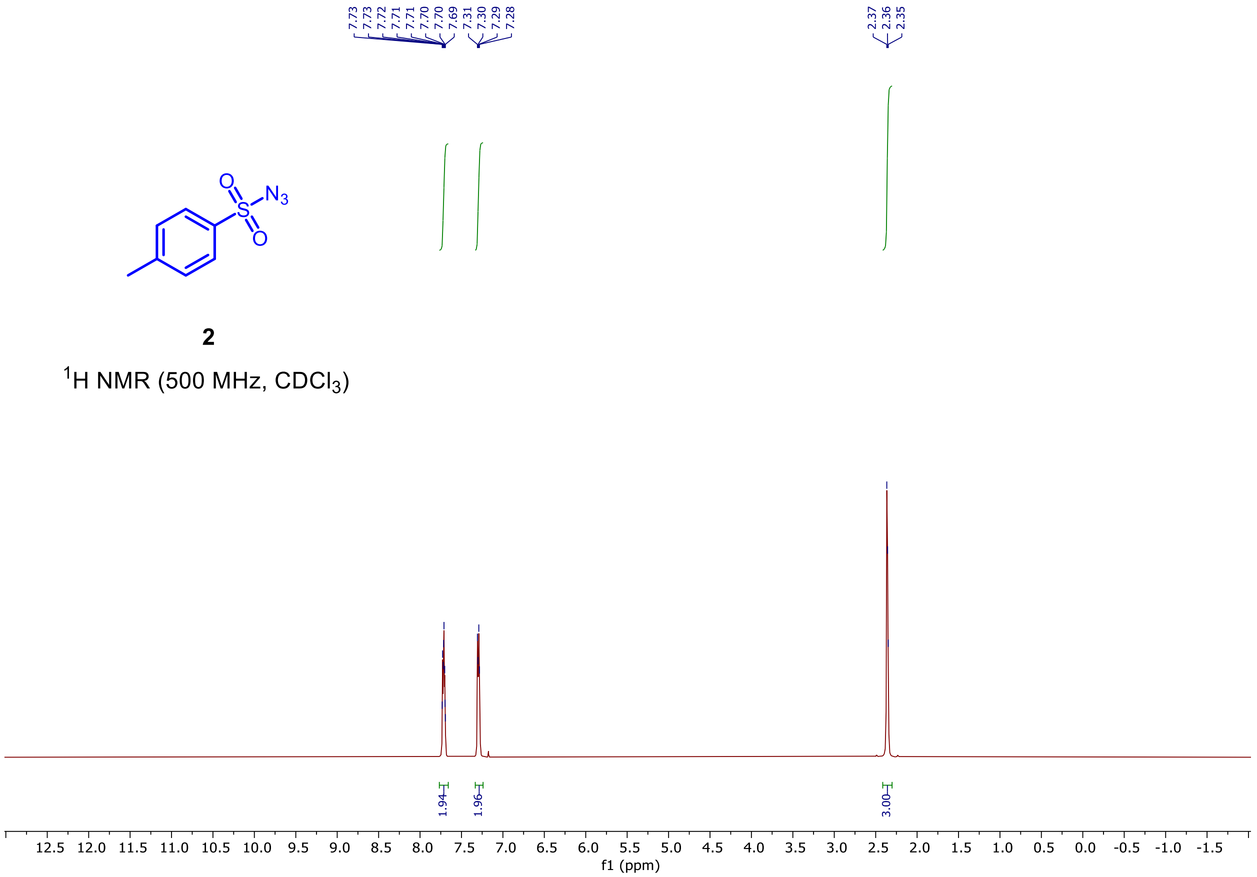

2

^{1}H NMR (500 MHz, $CDCl_3$)

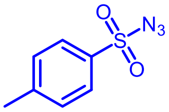

2

^{13}C NMR (126 MHz, $CDCl_3$)

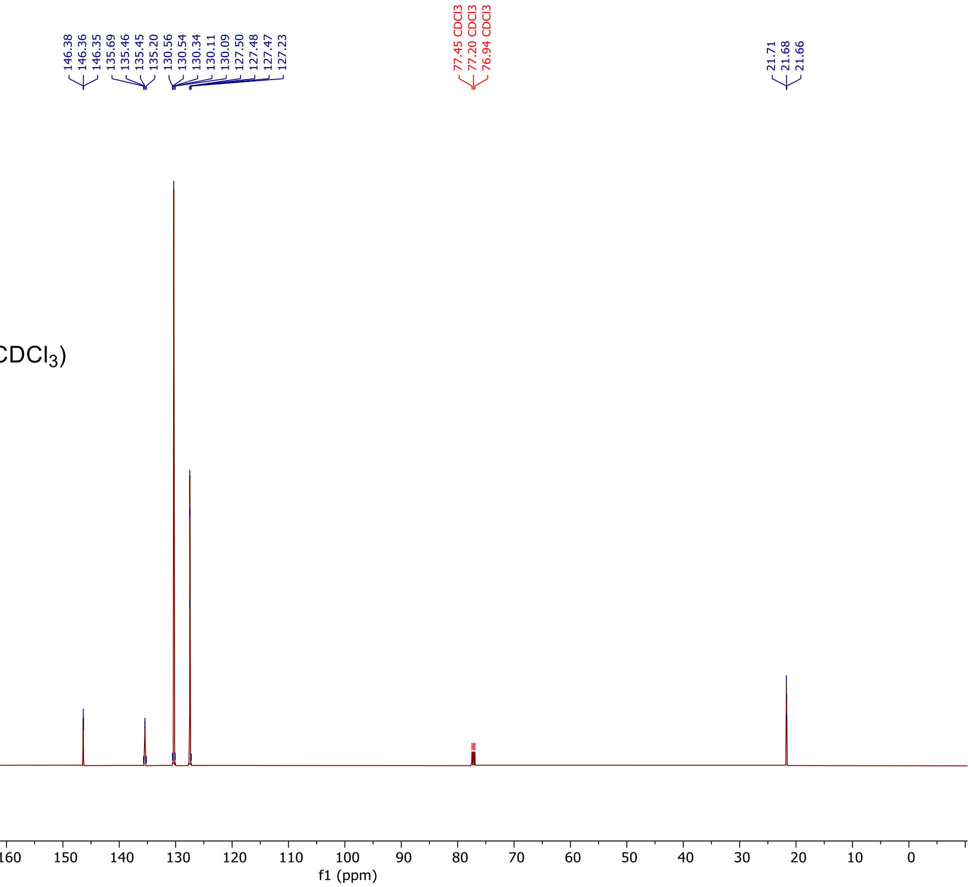

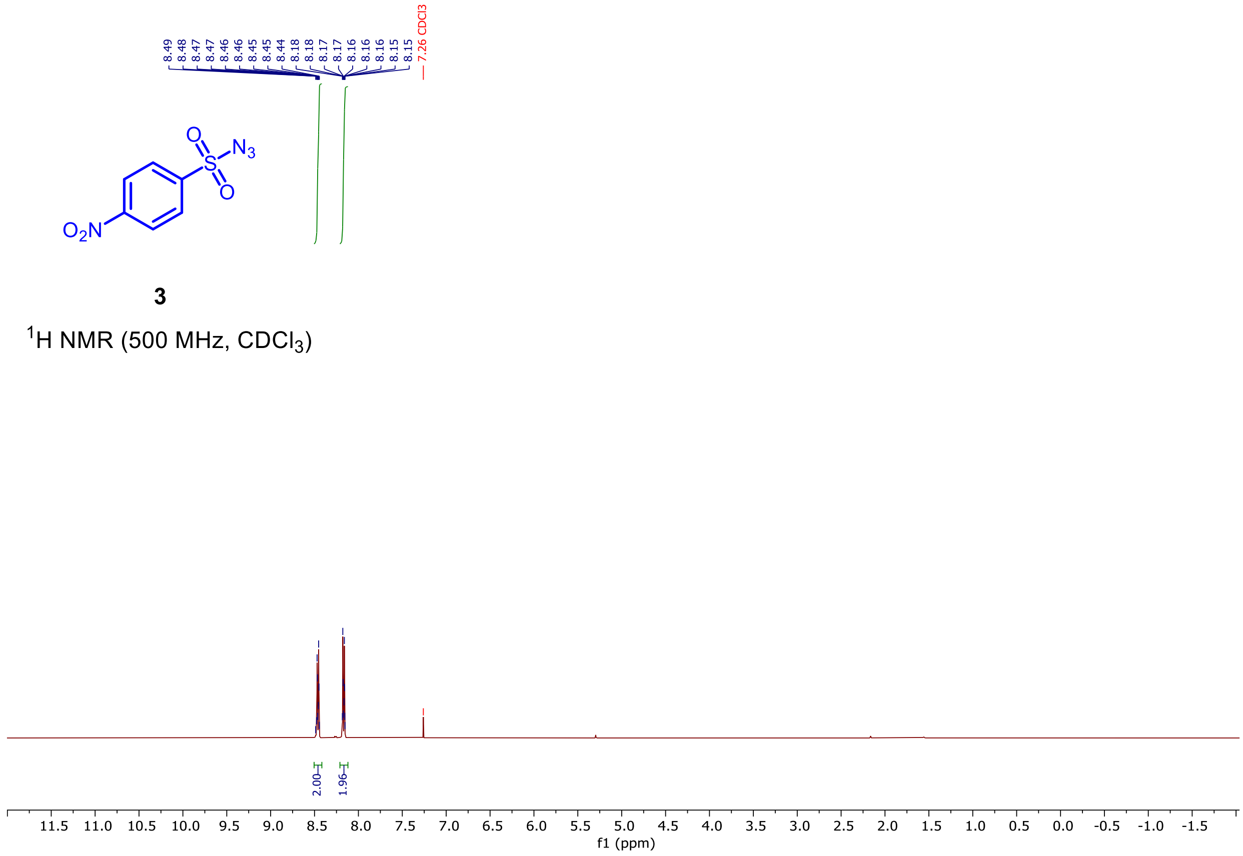

3

^{1}H NMR (500 MHz, $CDCl_3$)

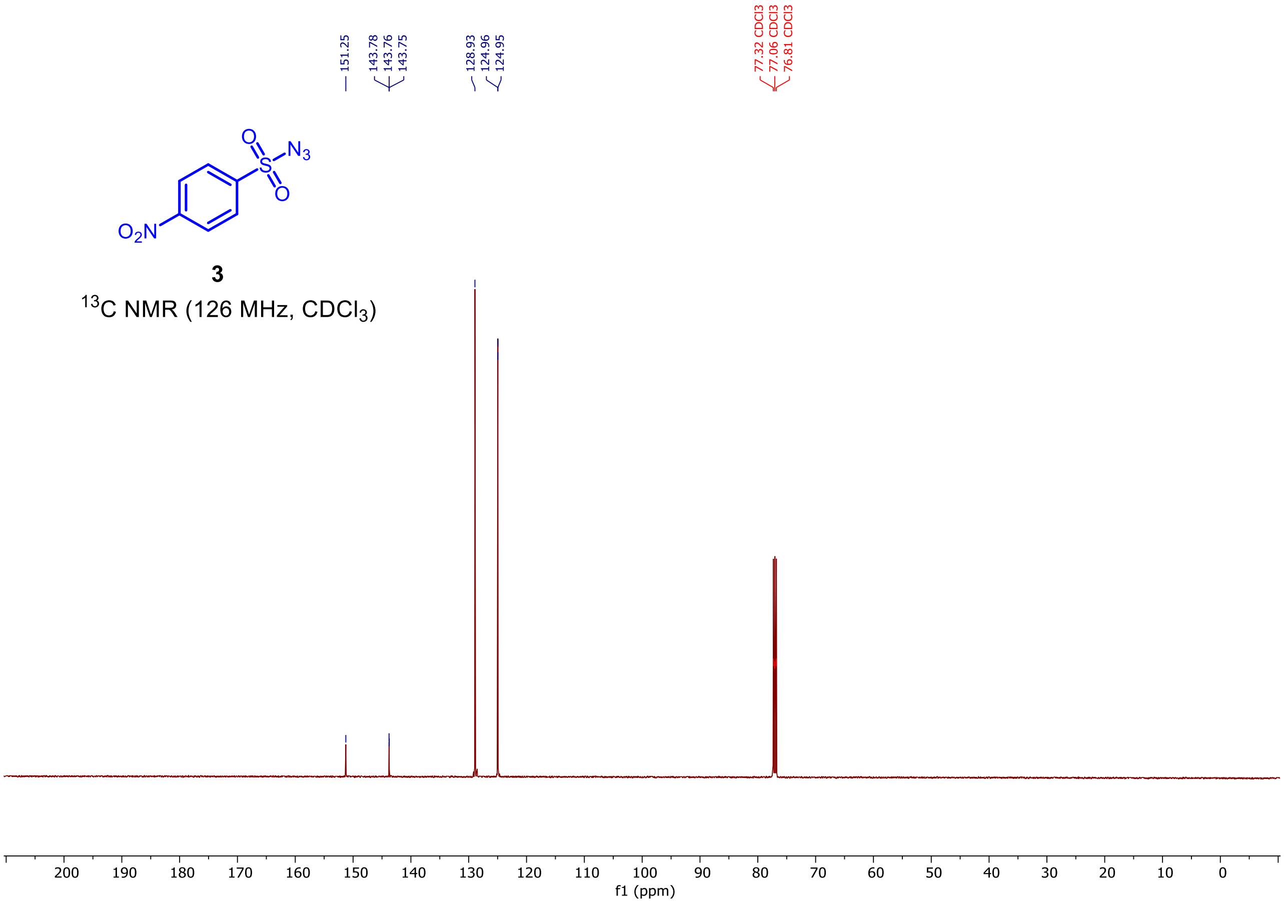

3

^{13}C NMR (126 MHz, $CDCl_3$)

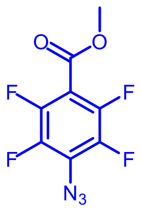

4
^{1}H NMR (500 MHz, $CDCl_3$)

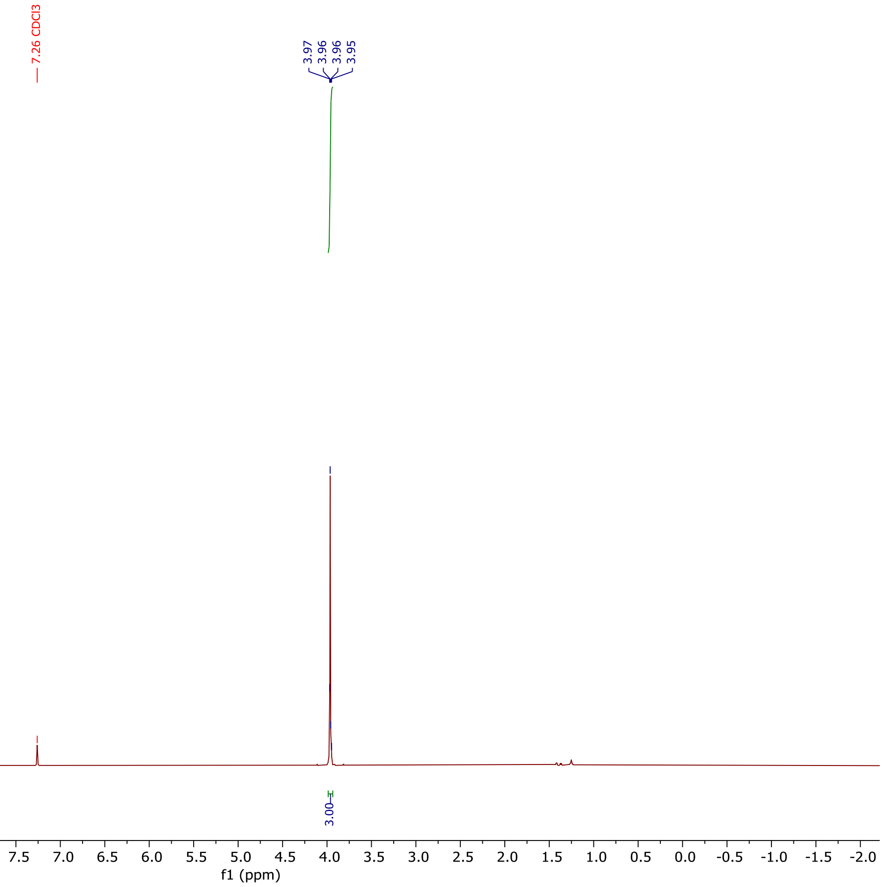

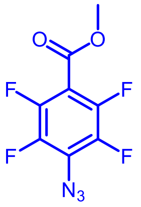

4

^{19}F NMR (471 MHz, $CDCl_3$)

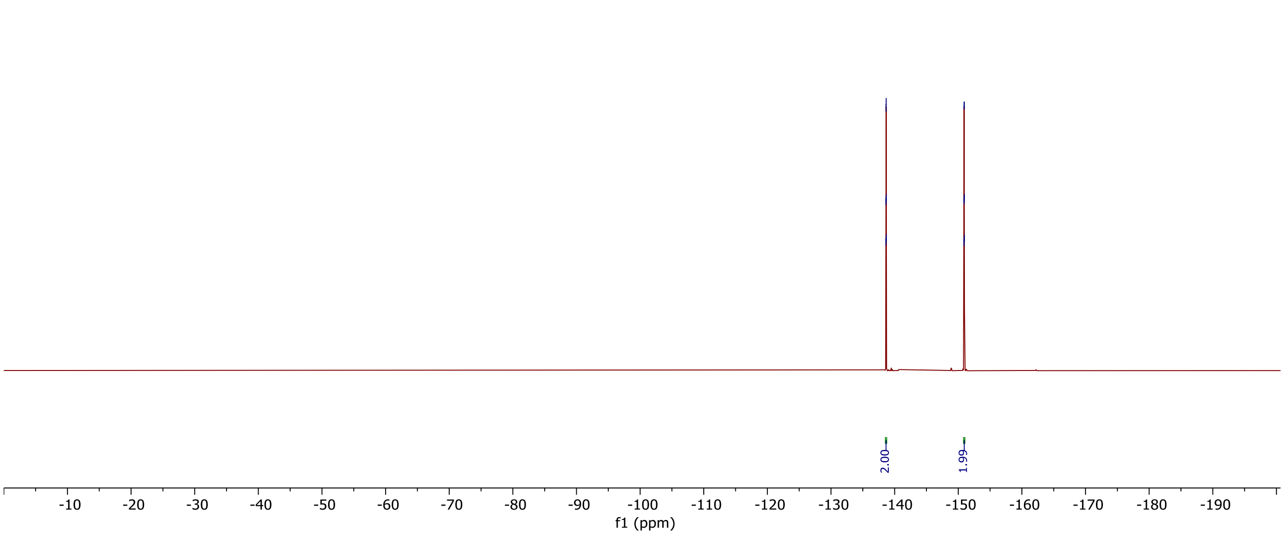

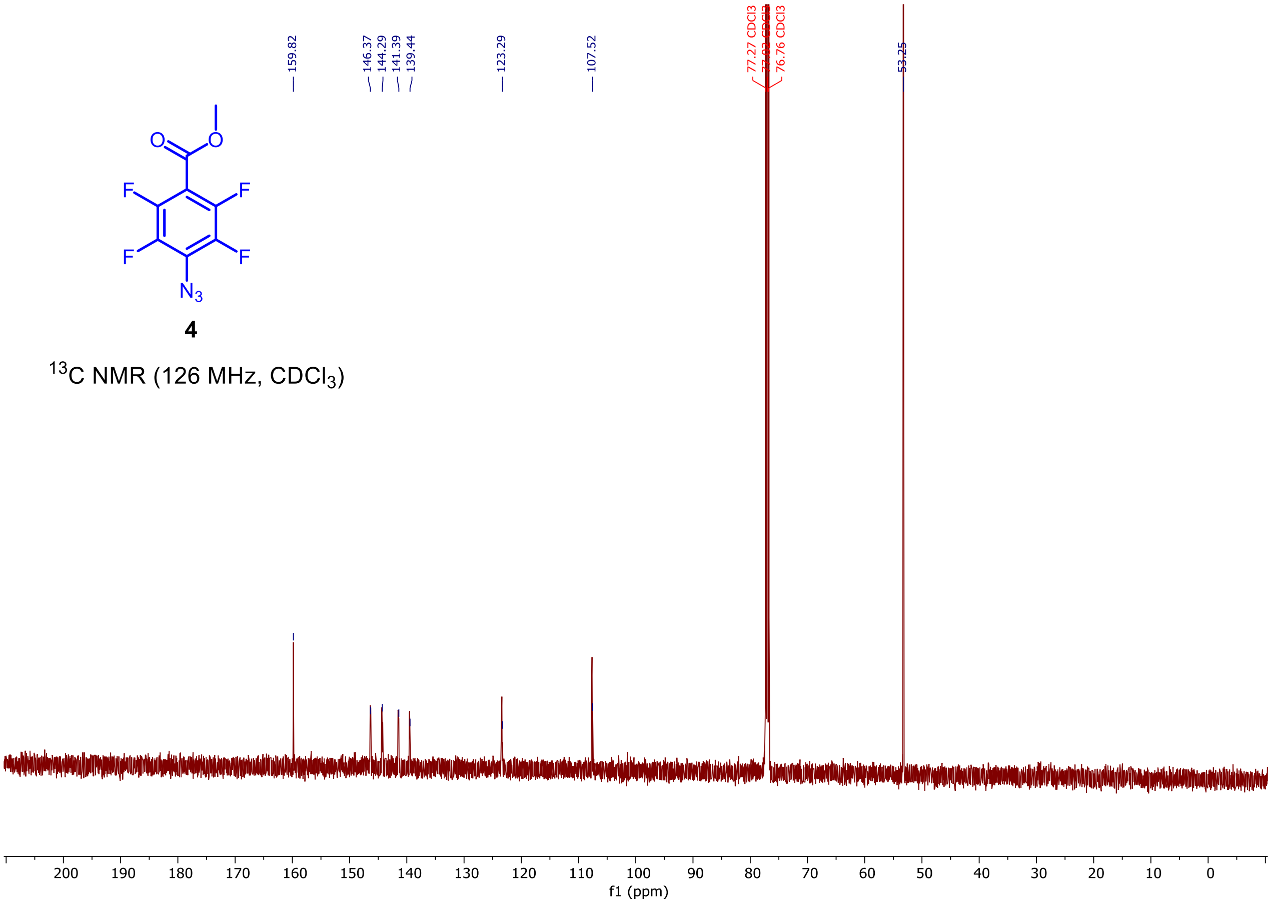

159.82
146.37
144.29
141.39
139.44
123.29
107.52
77.27 CDCl3
76.76 CDCl3
53.25
O
O
F
F
F
F
N3
4
^{13}C NMR (126 MHz, $CDCl_3$)
200 190 180 170 160 150 140 130 120 110 100 90 80 70 60 50 40 30 20 10 0
f1 (ppm)

S

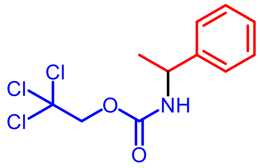

5

^{1}H NMR (500 MHz, $CDCl_3$)

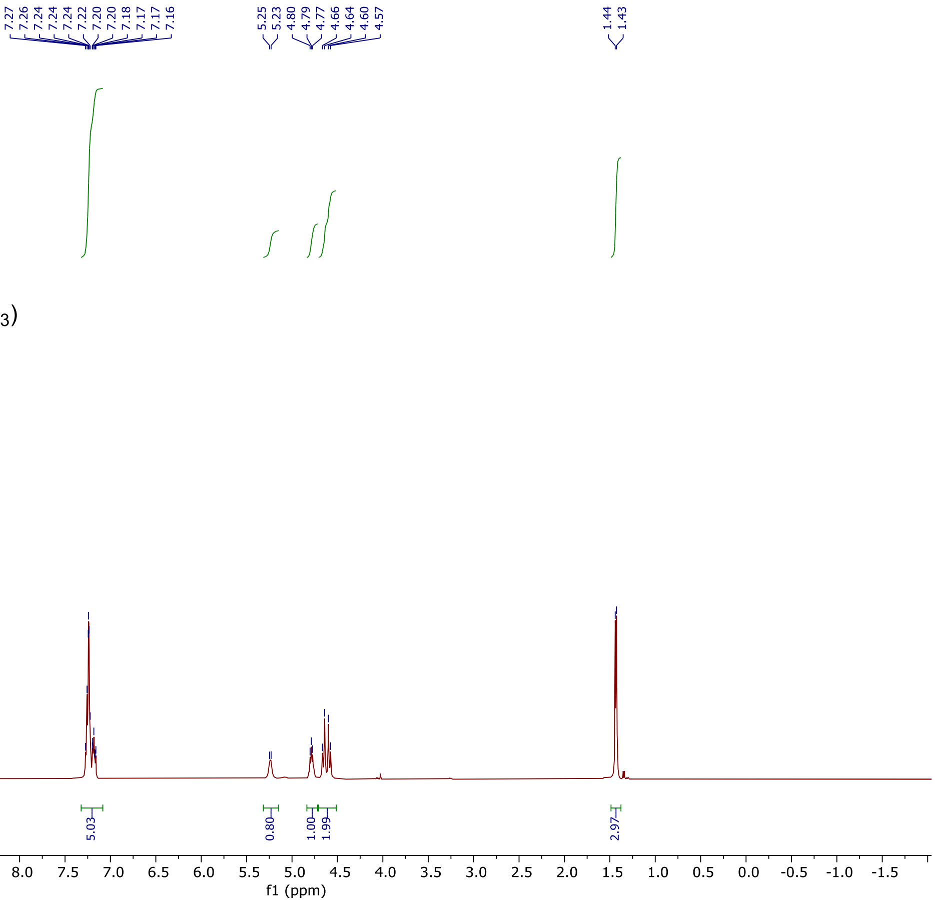

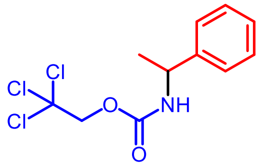

5

^{13}C NMR (126 MHz, $CDCl_3$)

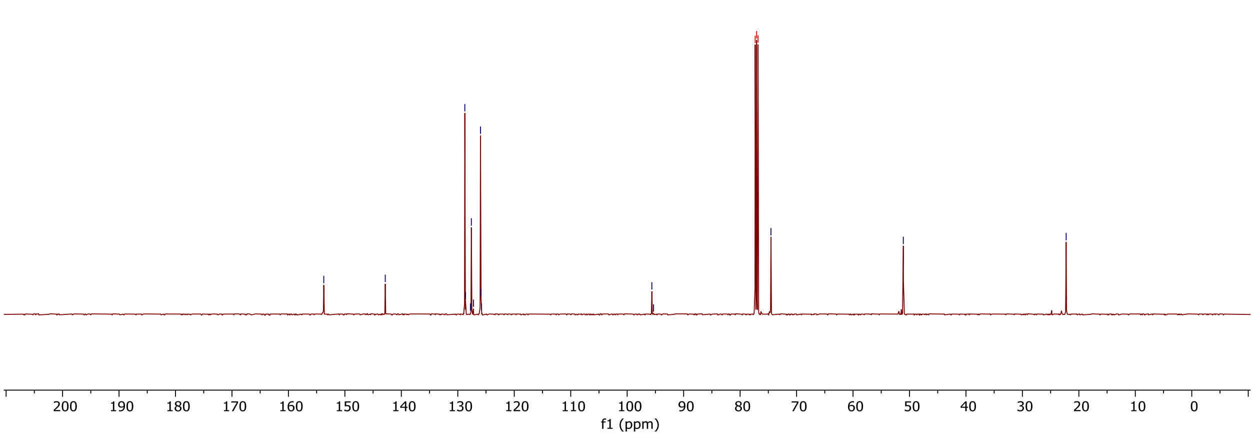

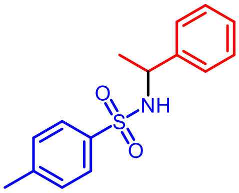

6

^{1}H NMR (500 MHz, $CDCl_3$)

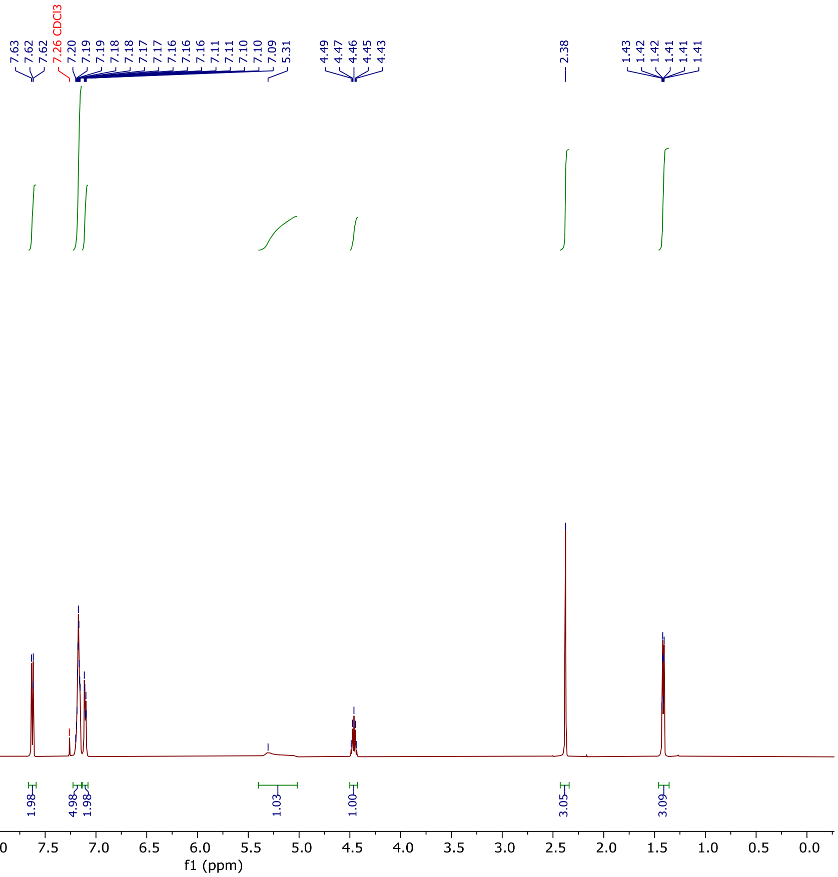

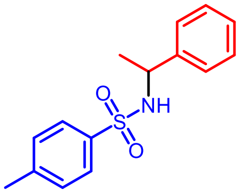

6

^{13}C NMR (126 MHz, $CDCl_3$)

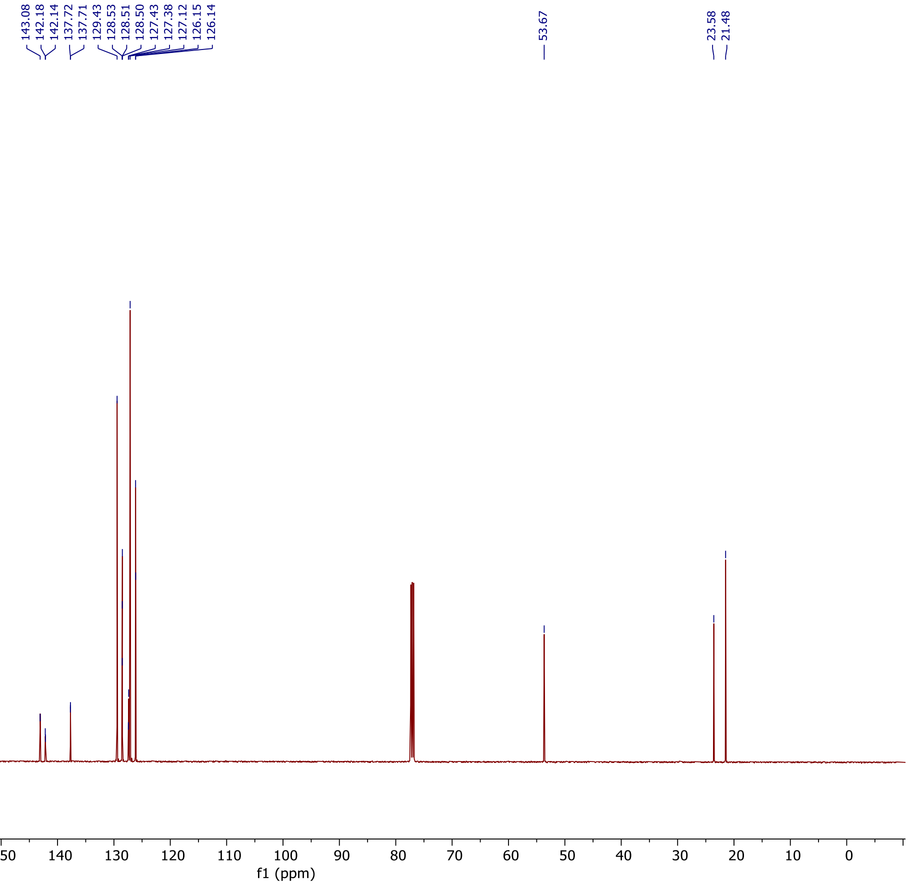

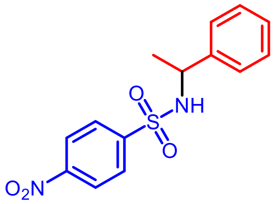

7

^{1}H NMR (500 MHz, DMSO-d_6)

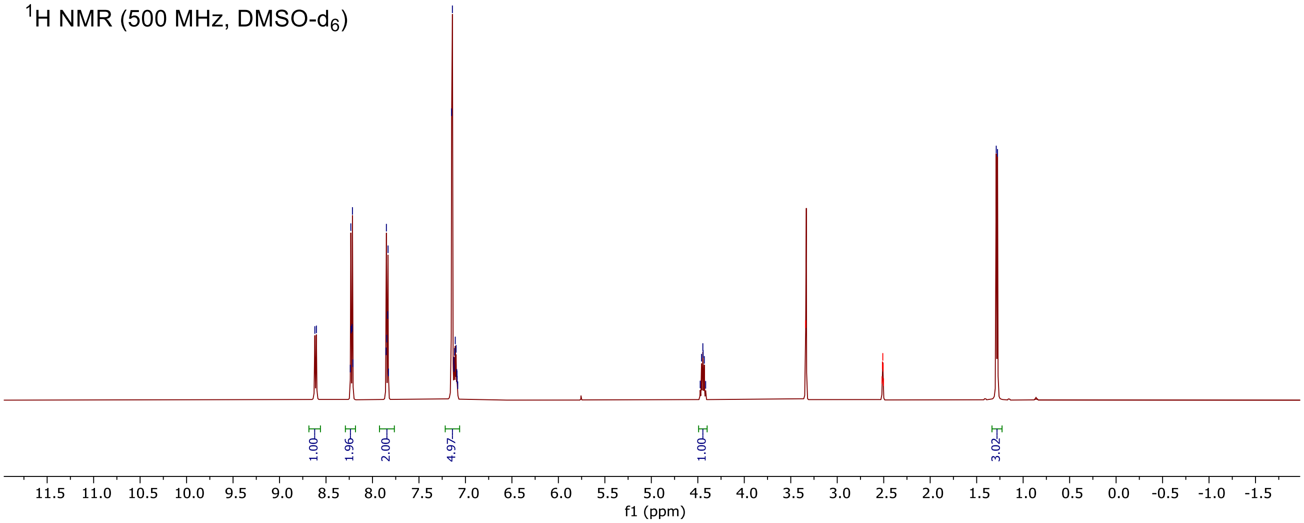

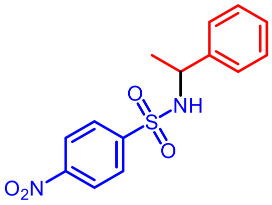

7

^{13}C NMR (126 MHz, DMSO-d_6)

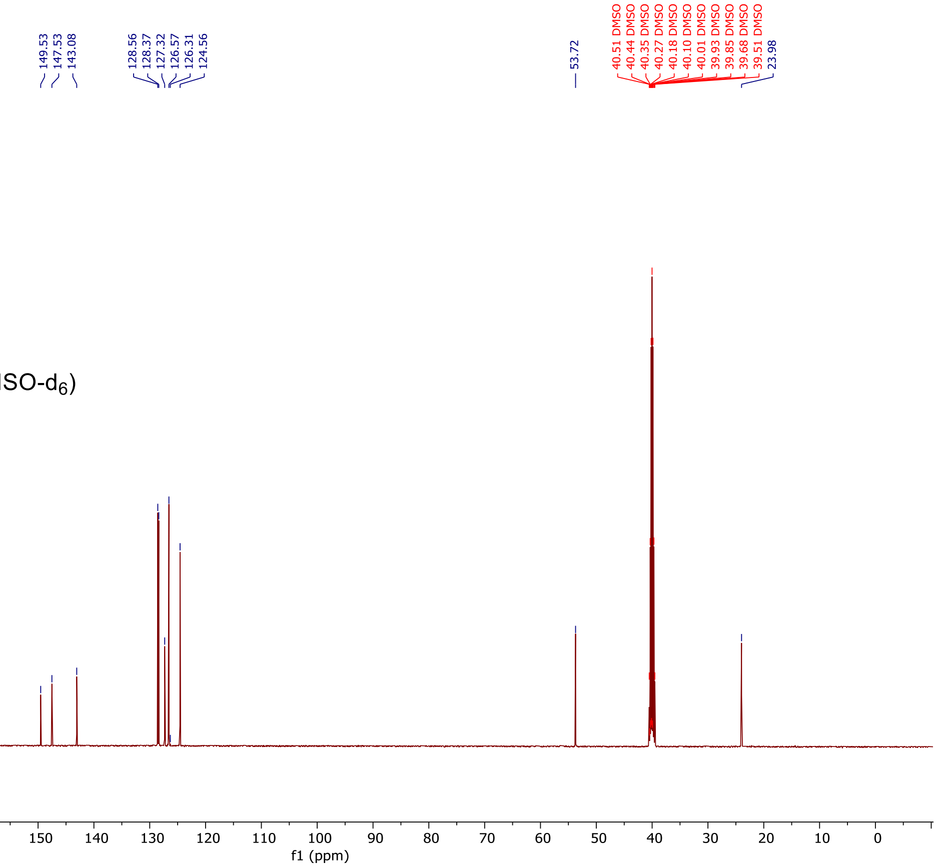

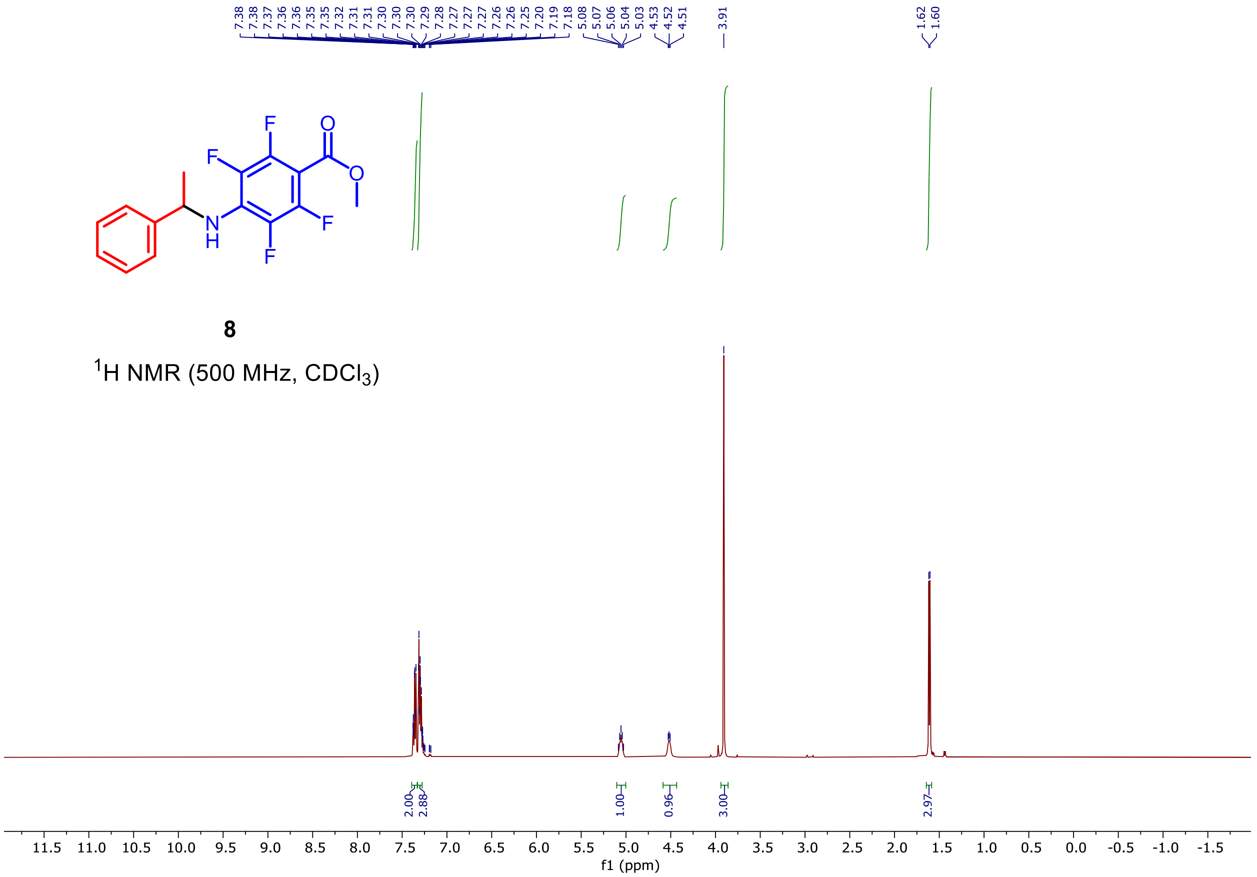

8
^{1}H NMR (500 MHz, CDCl$_3$)
7.38 7.38 7.37 7.36 7.36 7.35 7.35 7.32 7.31 7.31 7.30 7.30 7.30 7.29 7.28 7.27 7.27 7.27 7.26 7.26 7.25 7.20 7.19 7.18 5.08 5.07 5.06 5.04 5.03 4.53 4.52 4.51 3.91 1.62 1.60
2.00 2.88 1.00 0.96 3.00 2.97
11.5 11.0 10.5 10.0 9.5 9.0 8.5 8.0 7.5 7.0 6.5 6.0 5.5 5.0 4.5 4.0 3.5 3.0 2.5 2.0 1.5 1.0 0.5 0.0 -0.5 -1.0 -1.5
f1 (ppm)

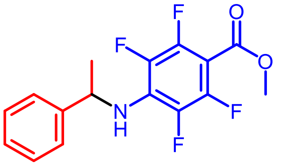

8

^{19}F NMR (471 MHz, $CDCl_3$)

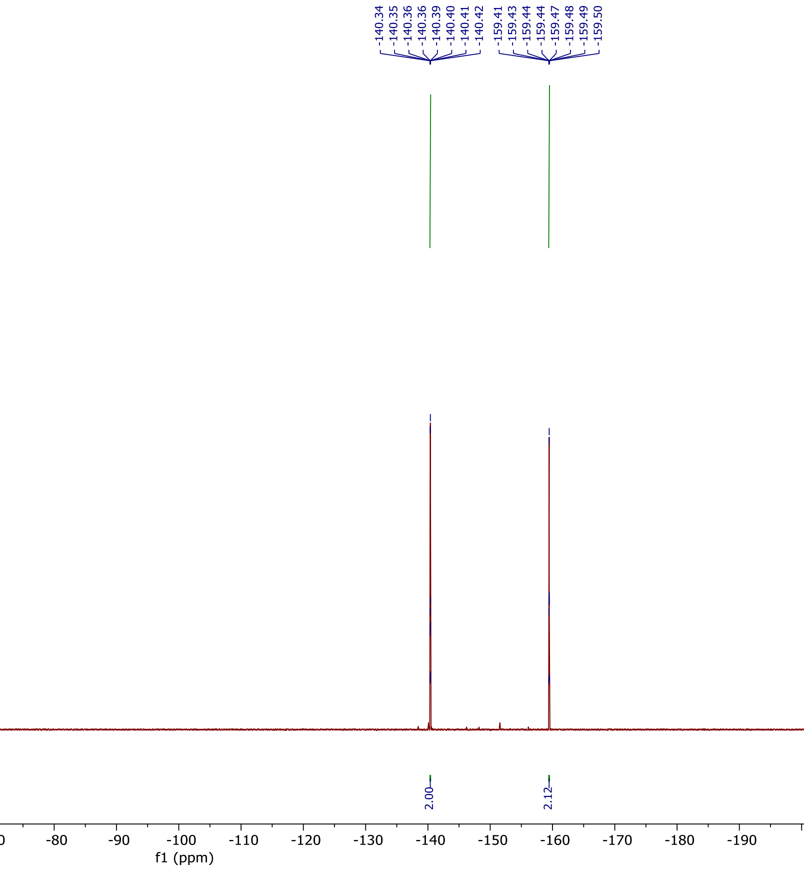

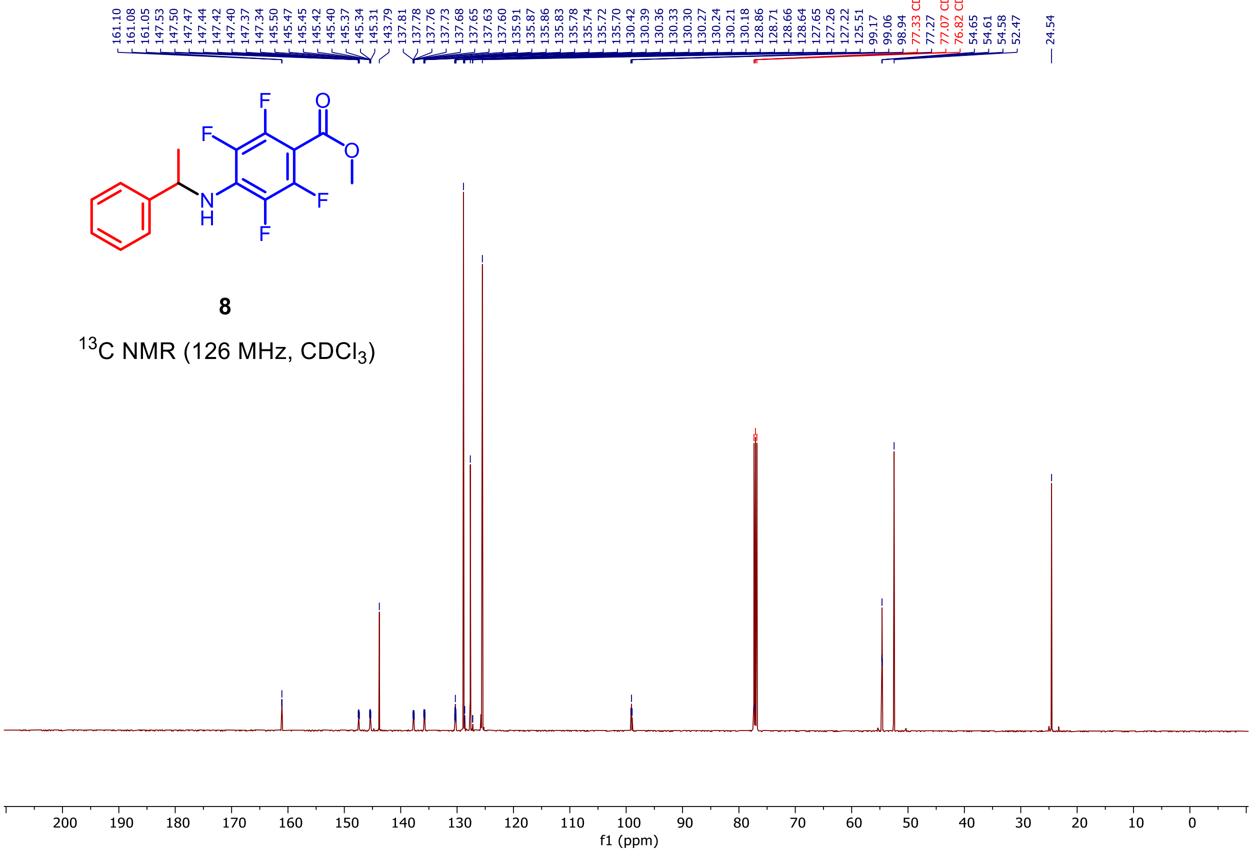

8

^{13}C NMR (126 MHz, $CDCl_3$)

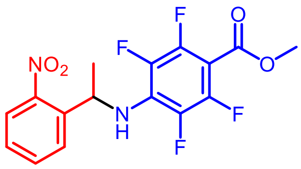

9

^{1}H NMR (500 MHz, $CDCl_3$)

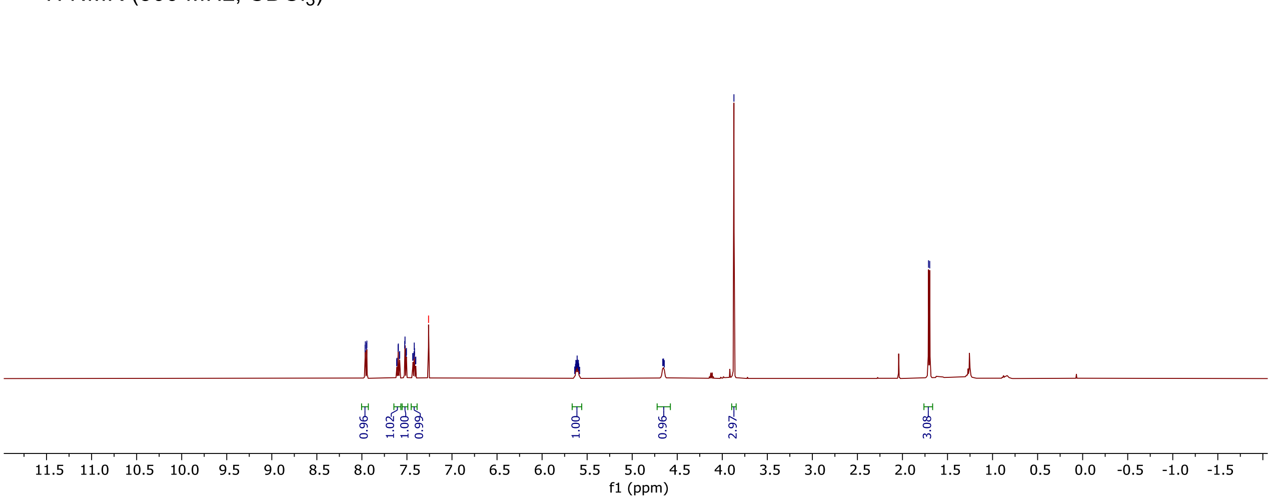

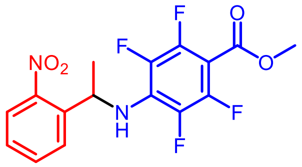

9

^{19}F NMR (471 MHz, $CDCl_3$)

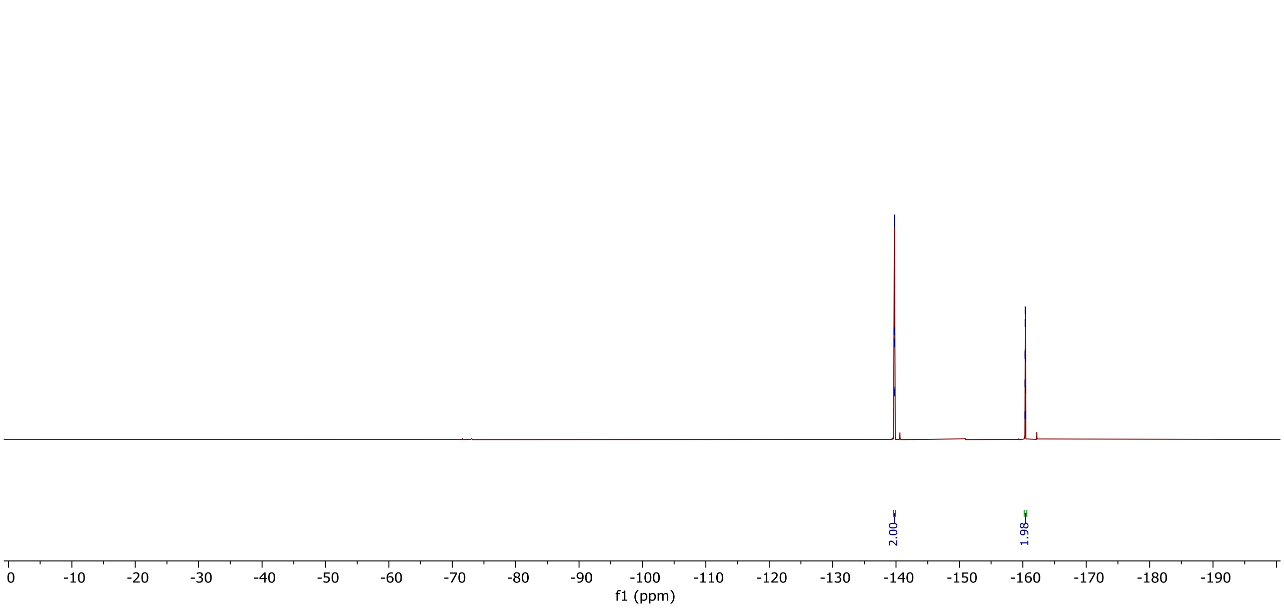

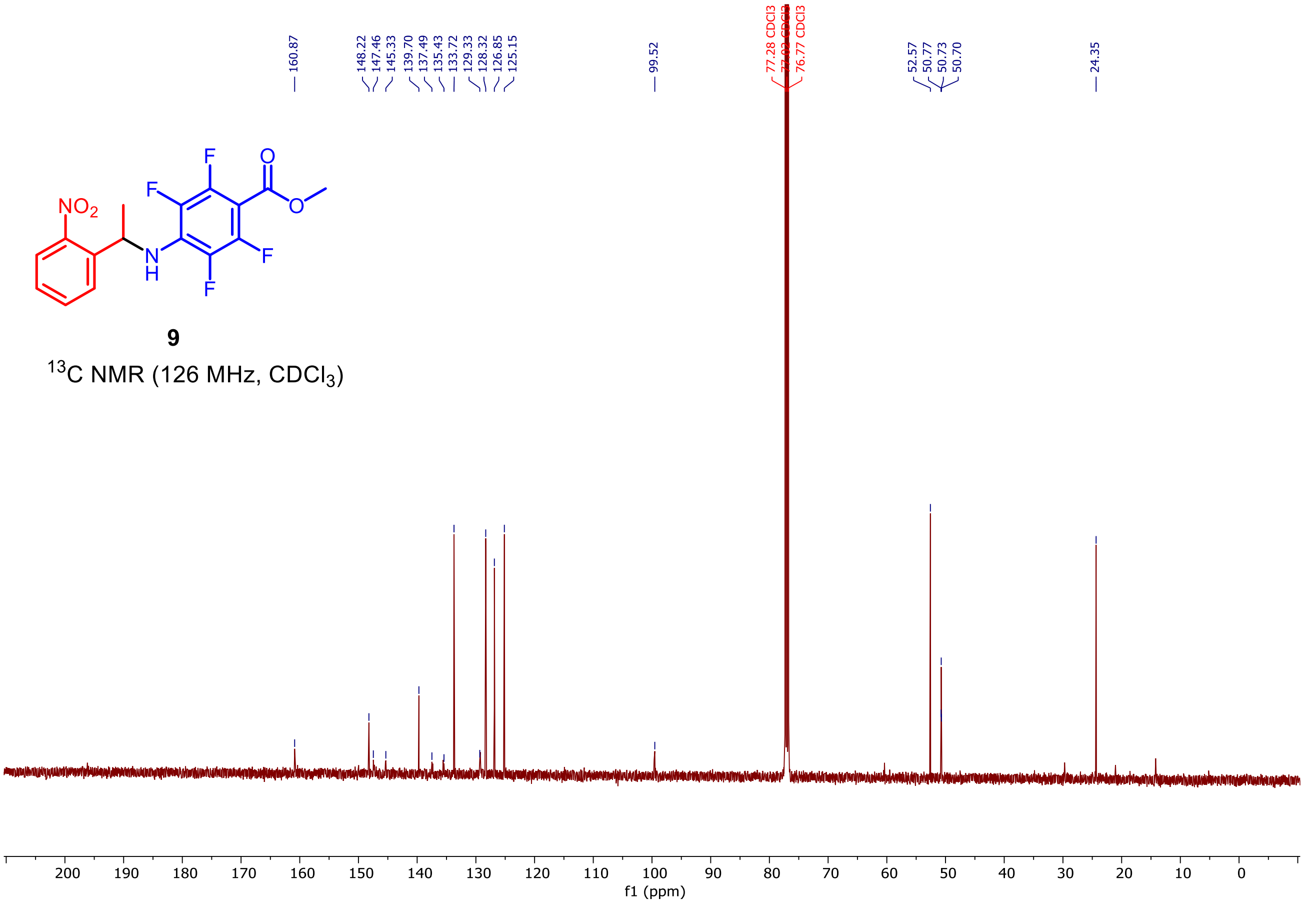

9

^{13}C NMR (126 MHz, $CDCl_3$)

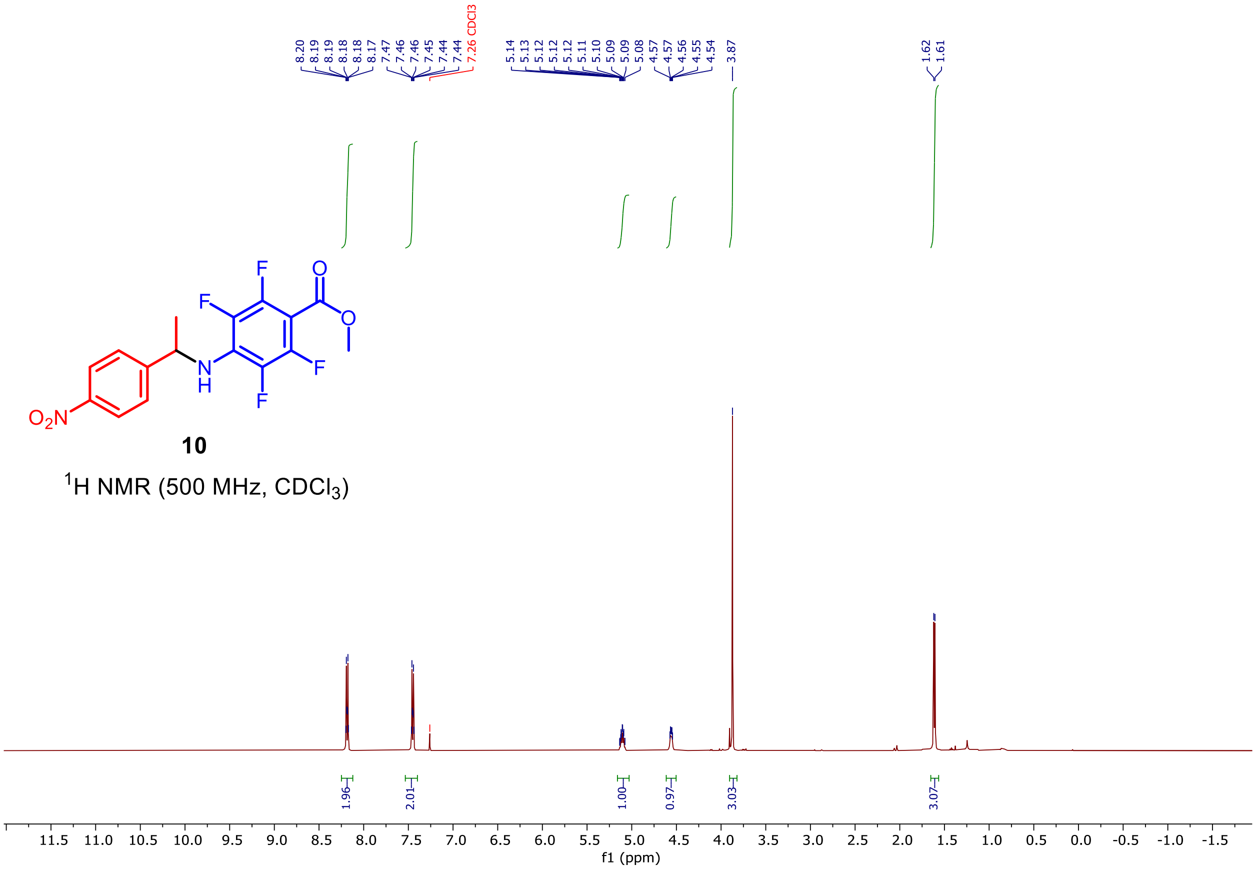

10
^{1}H NMR (500 MHz, CDCl$_3$)
8.20
8.19
8.19
8.18
8.18
8.17
7.47
7.46
7.46
7.45
7.44
7.44
7.26 CDCl3
5.14
5.13
5.12
5.12
5.12
5.11
5.10
5.09
5.09
5.08
4.57
4.57
4.56
4.55
4.54
3.87
1.62
1.61
1.96
2.01
1.00
0.97
3.03
3.07
f1 (ppm)

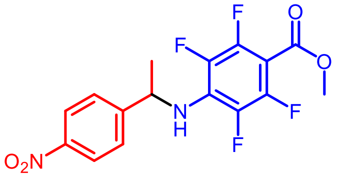

10

^{19}F NMR (471 MHz, $CDCl_3$)

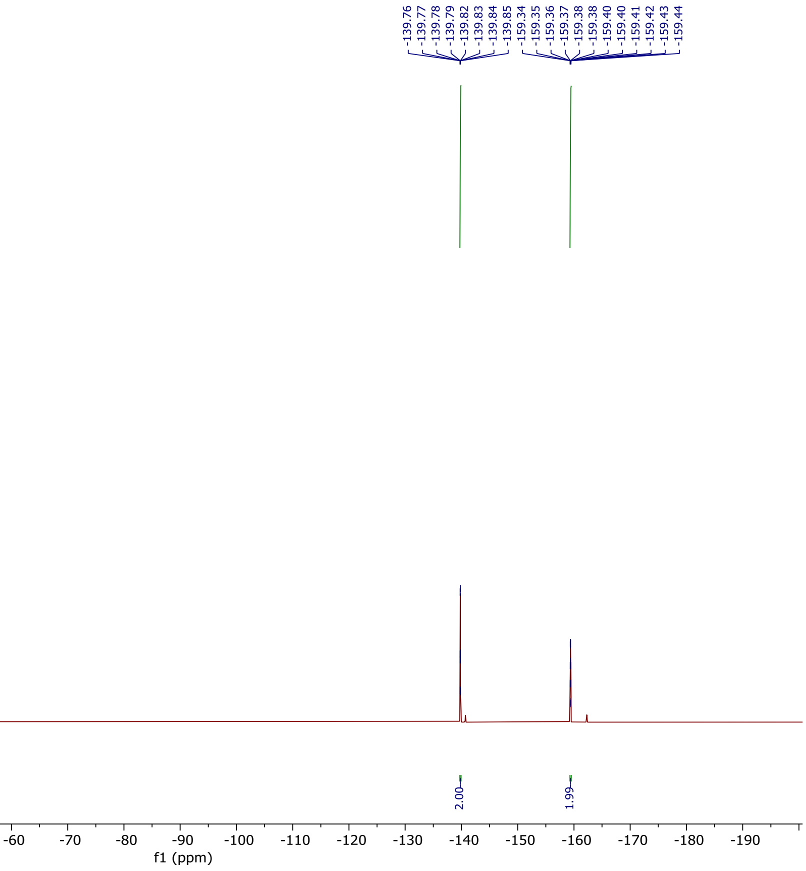

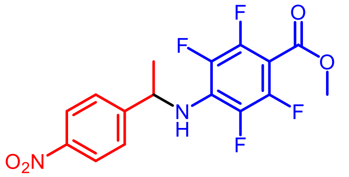

10

^{13}C NMR (126 MHz, $CDCl_3$)

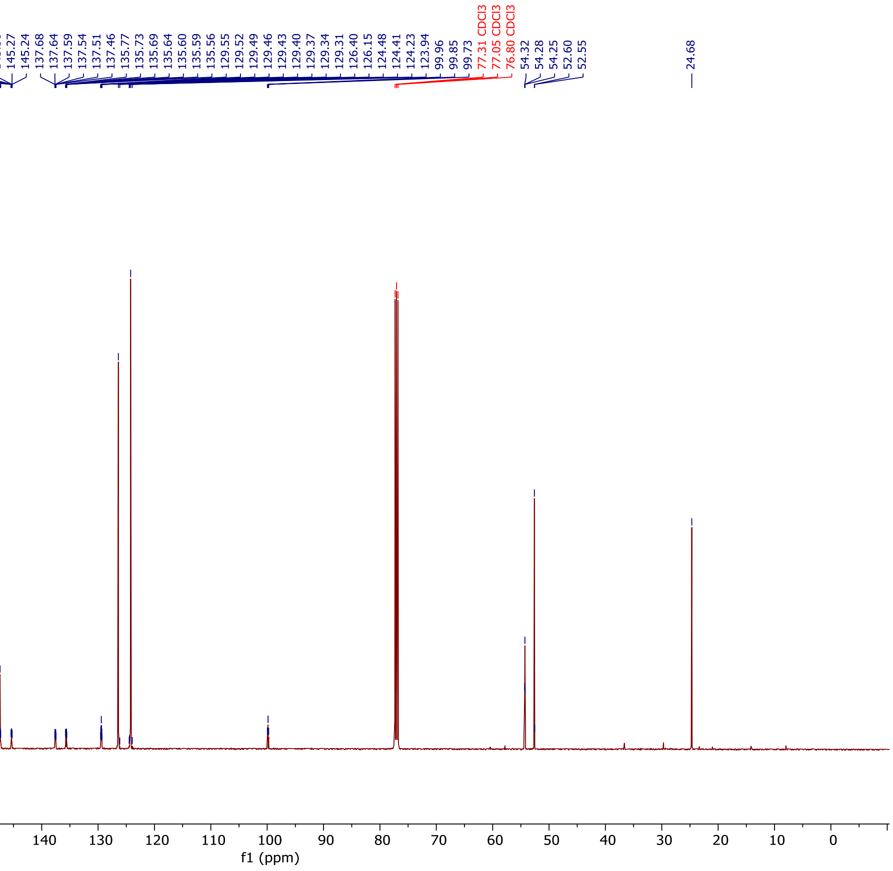

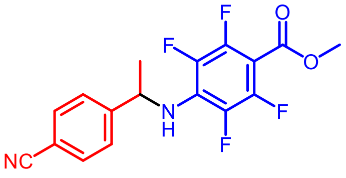

11

^{1}H NMR (500 MHz, $CDCl_3$)

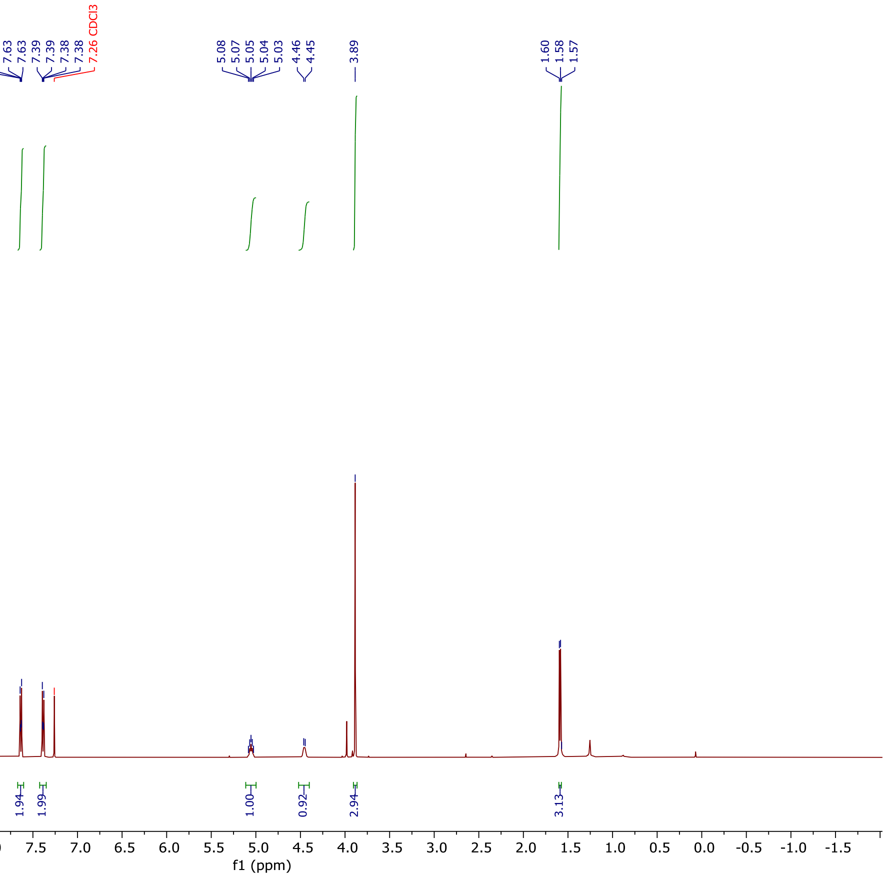

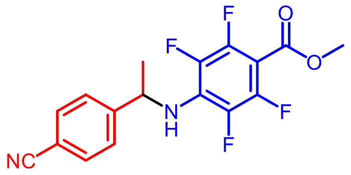

11

^{19}F NMR (471 MHz, $CDCl_3$)

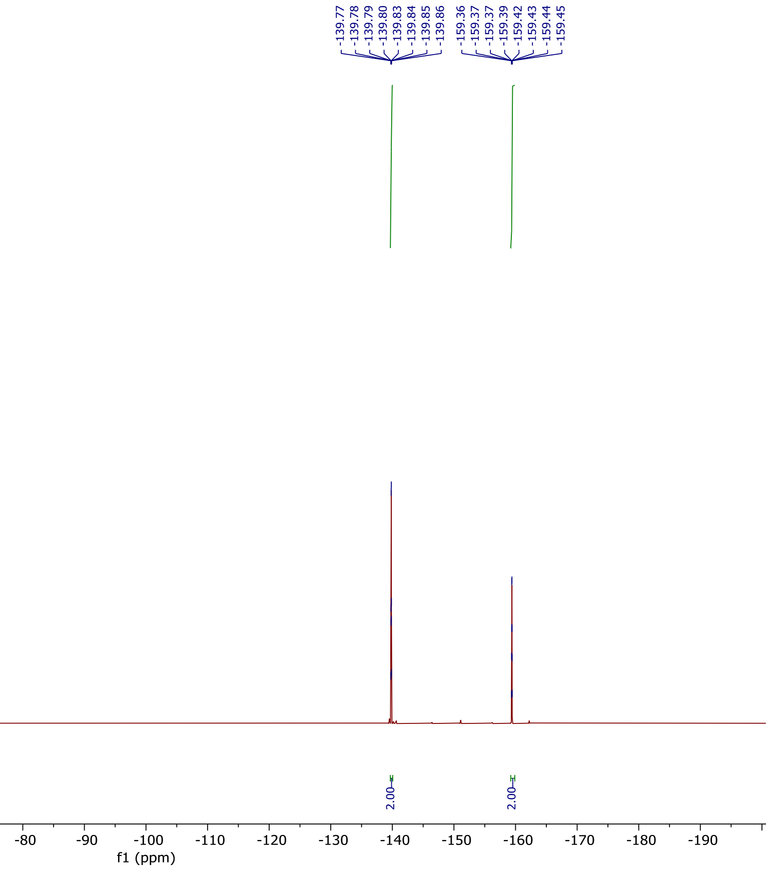

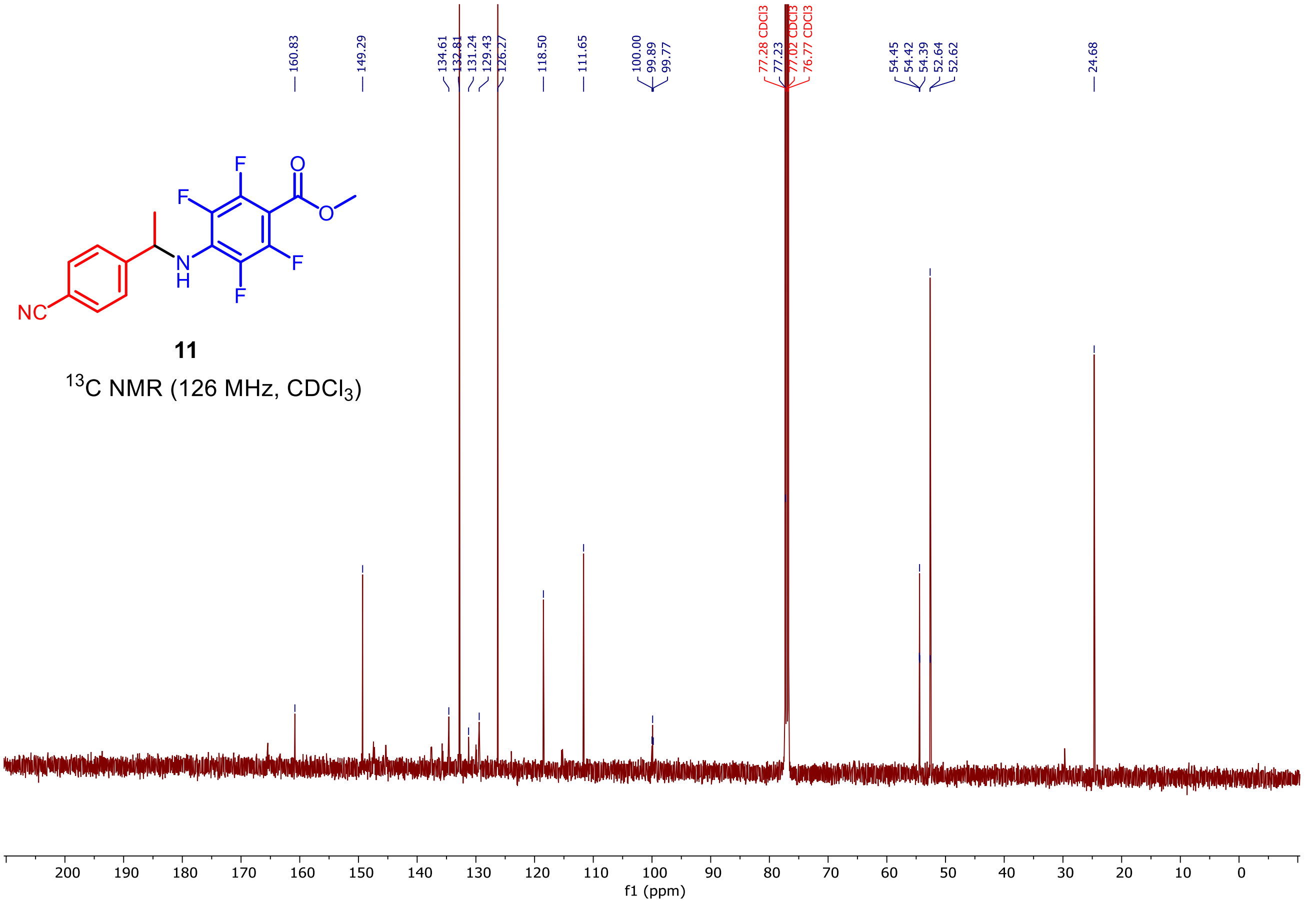

11

^{13}C NMR (126 MHz, $CDCl_3$)

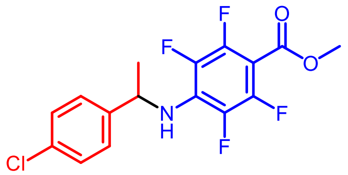

12

^{1}H NMR (500 MHz, $CDCl_3$)

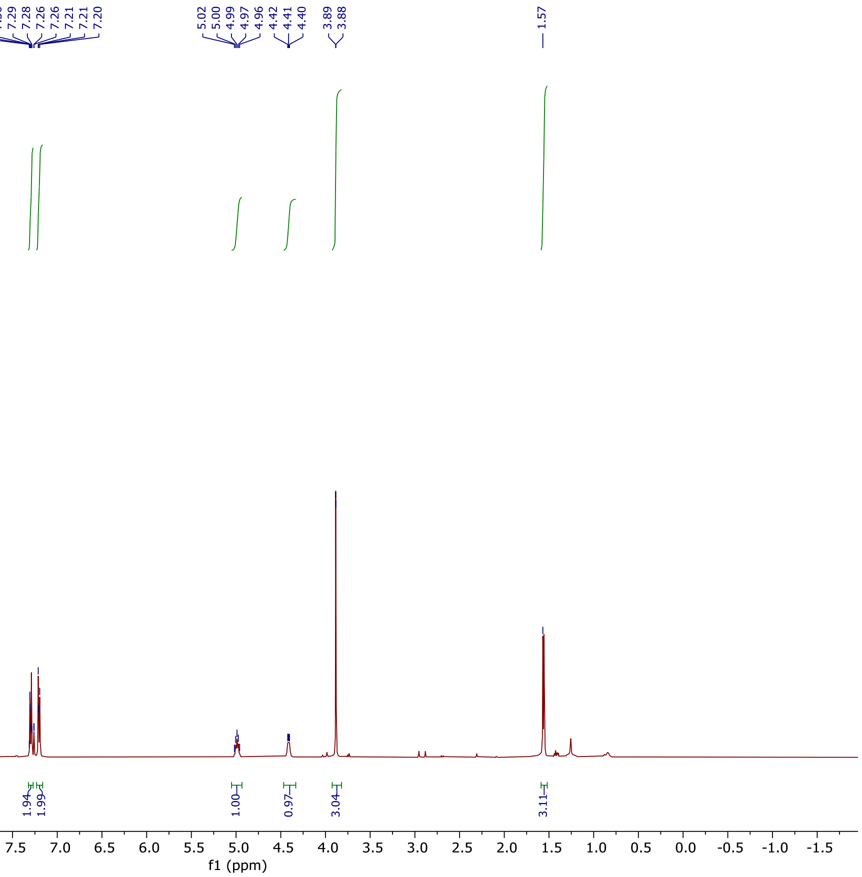

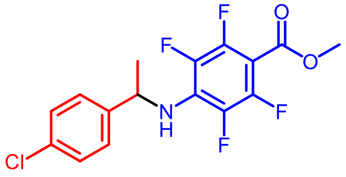

12

^{19}F NMR (471 MHz, $CDCl_3$)

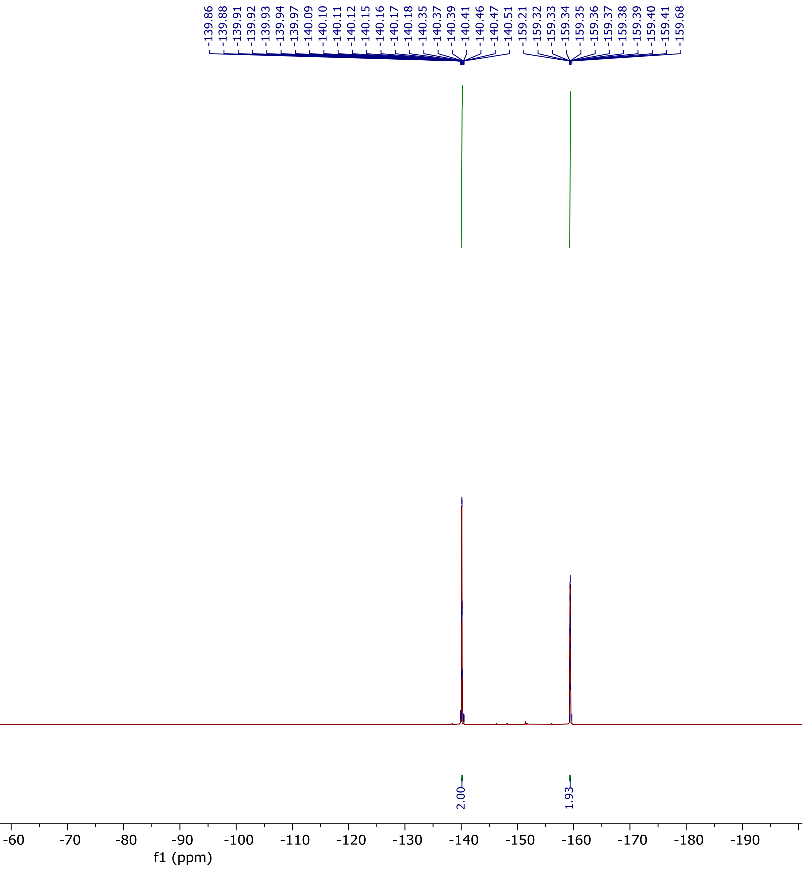

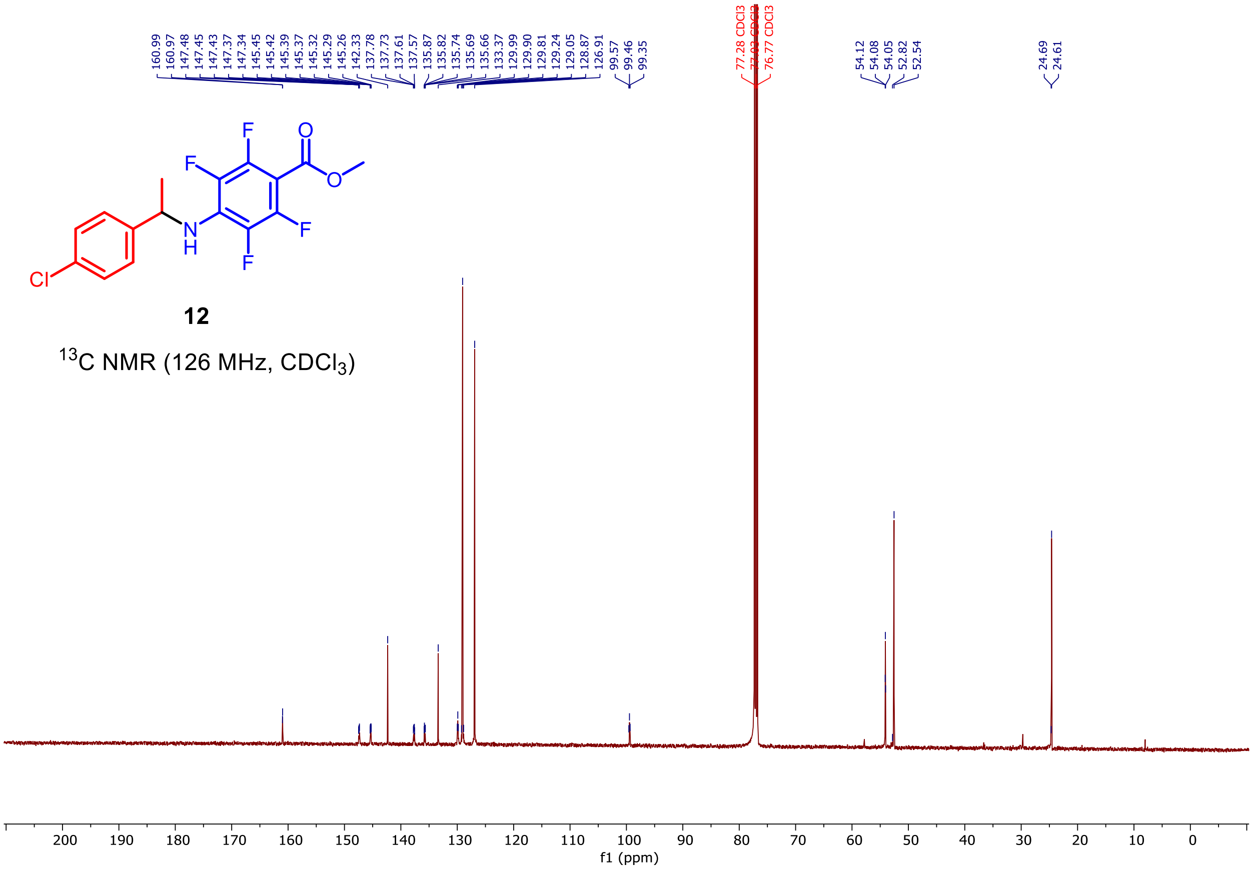

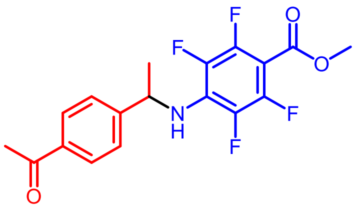

13

^{1}H NMR (500 MHz, $CDCl_3$)

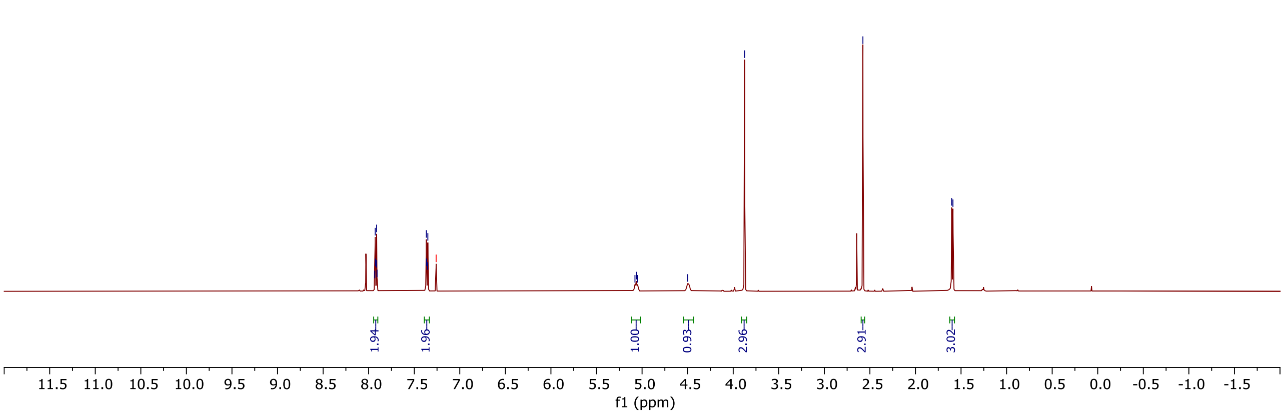

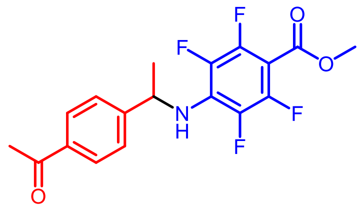

13

^{19}F NMR (471 MHz, $CDCl_3$)

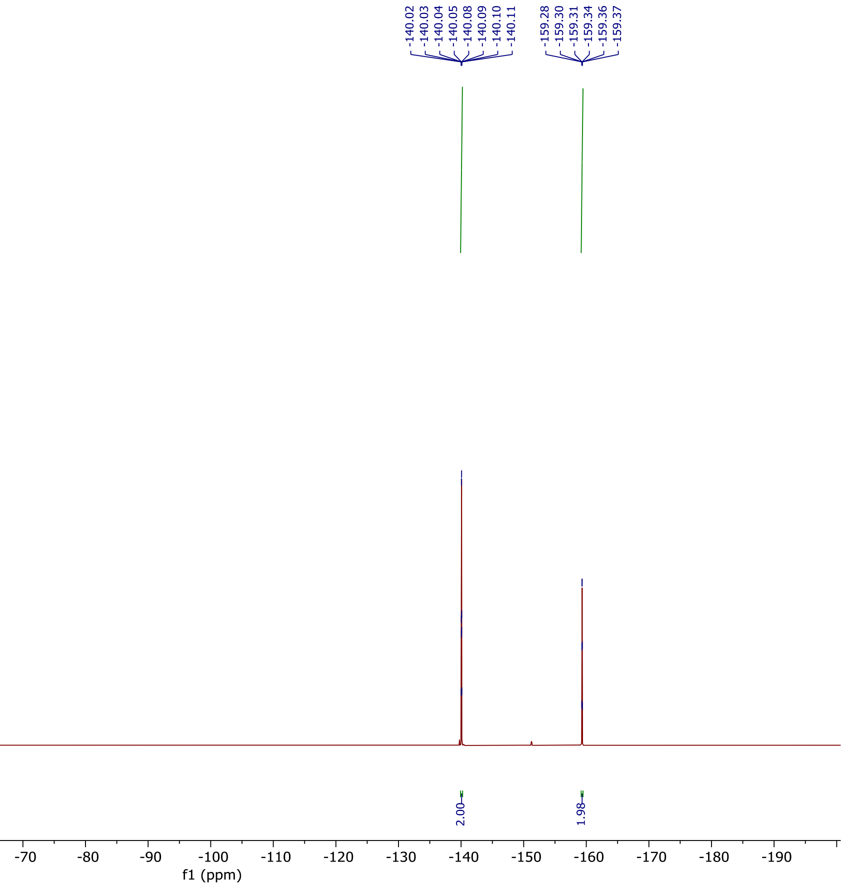

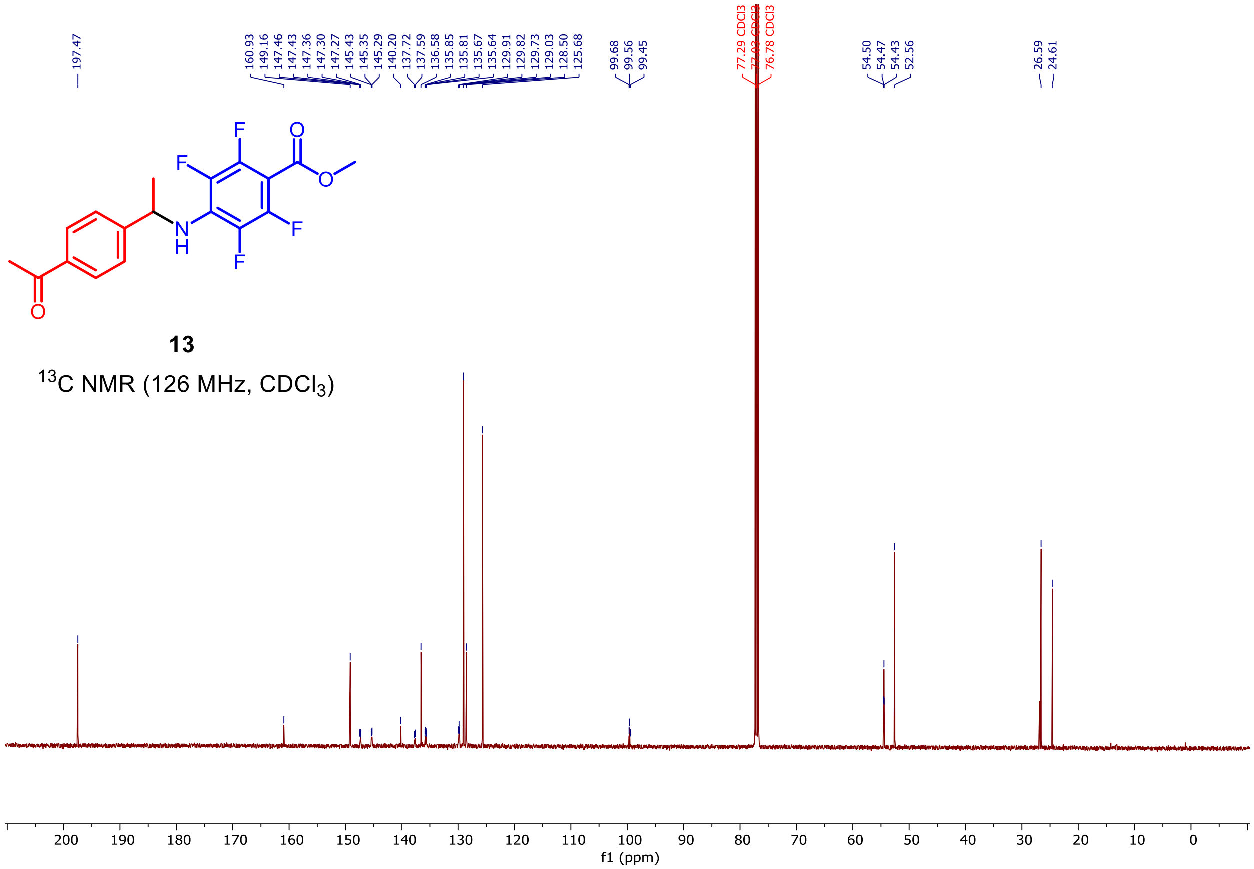
197.47
160.93
149.16
147.46
147.43
147.36
147.30
147.27
145.43
145.35
145.29
140.20
137.72
137.59
136.58
135.85
135.81
135.67
135.64
129.91
129.82
129.73
129.03
128.50
125.68
99.68
99.56
99.45
77.29 CDCl3
76.78 CDCl3
54.50
54.47
54.43
52.56
26.59
24.61
F
F
F
F
O
O
N
H
O
13
^{13}C NMR (126 MHz, $CDCl_3$)
200
190
180
170
160
150
140
130
120
110
100
90
80
70
60
50
40
30
20
10
0
f1 (ppm)

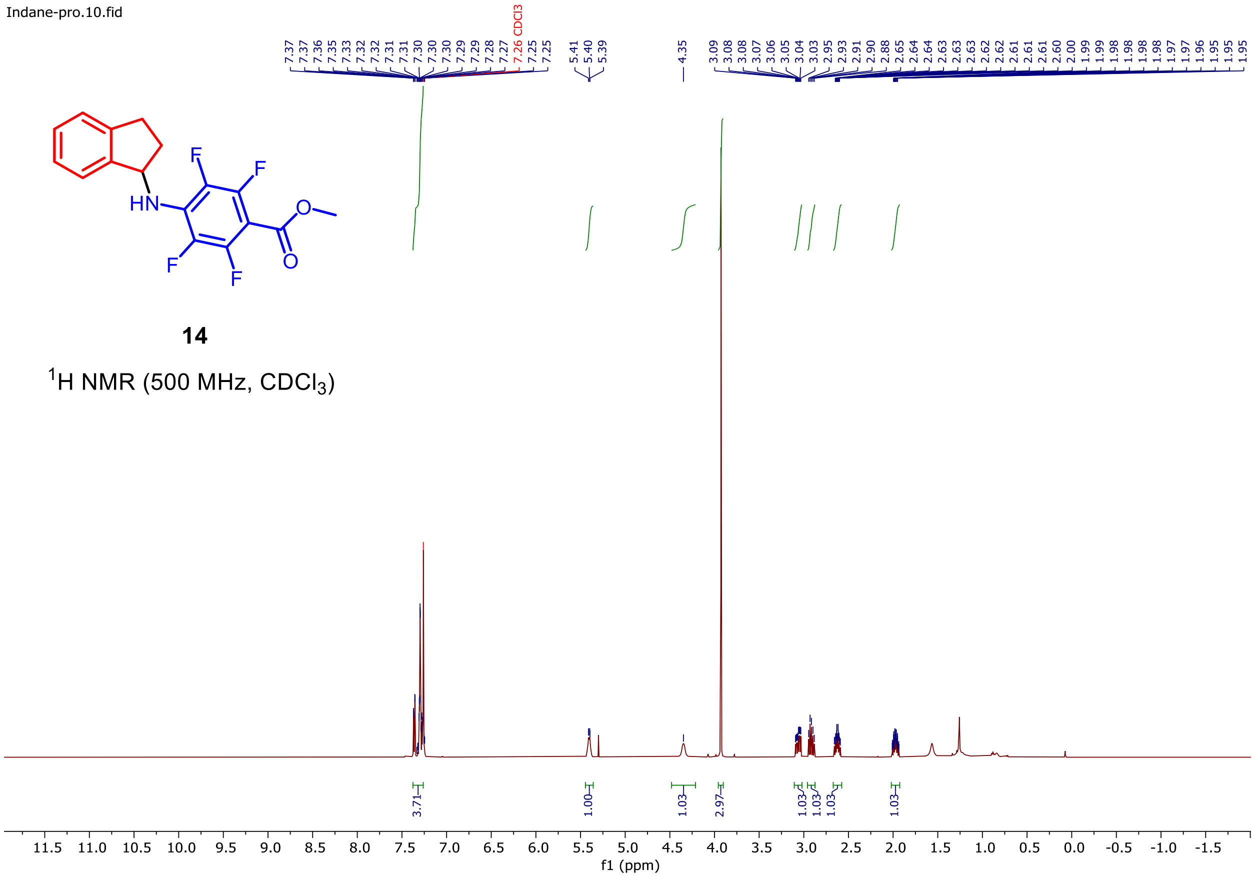
Indane-pro.10.fid
14
^{1}H NMR (500 MHz, CDCl$_3$)
f1 (ppm)

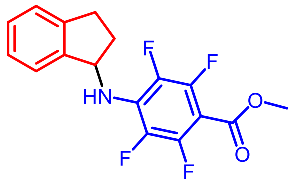

14

^{19}F NMR (471 MHz, $CDCl_3$)

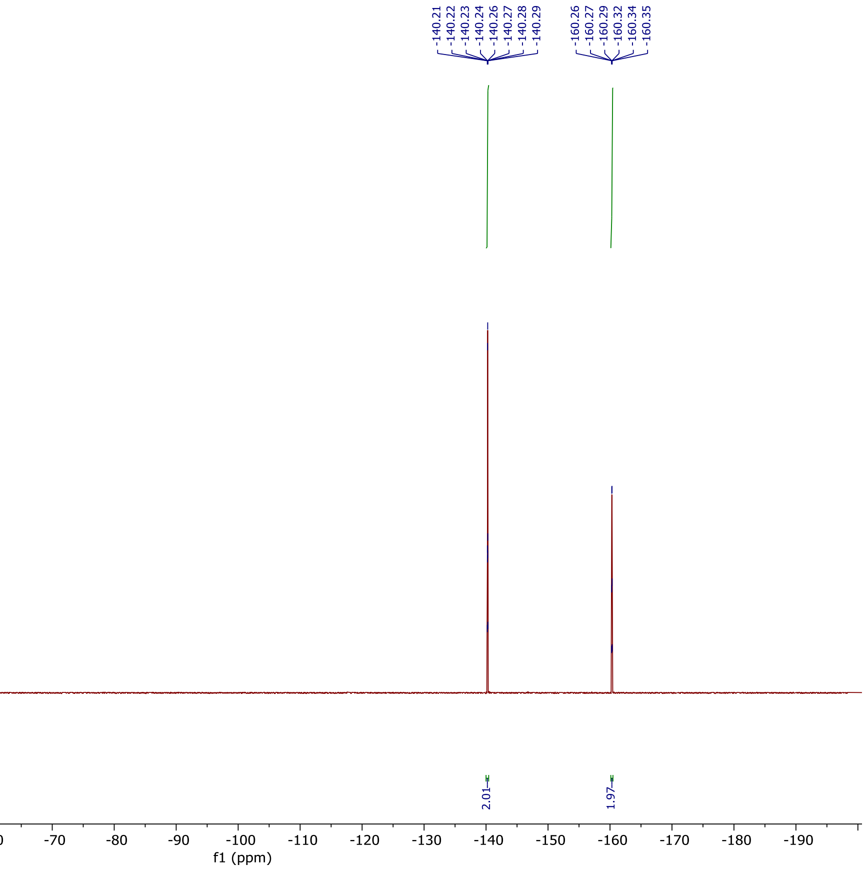

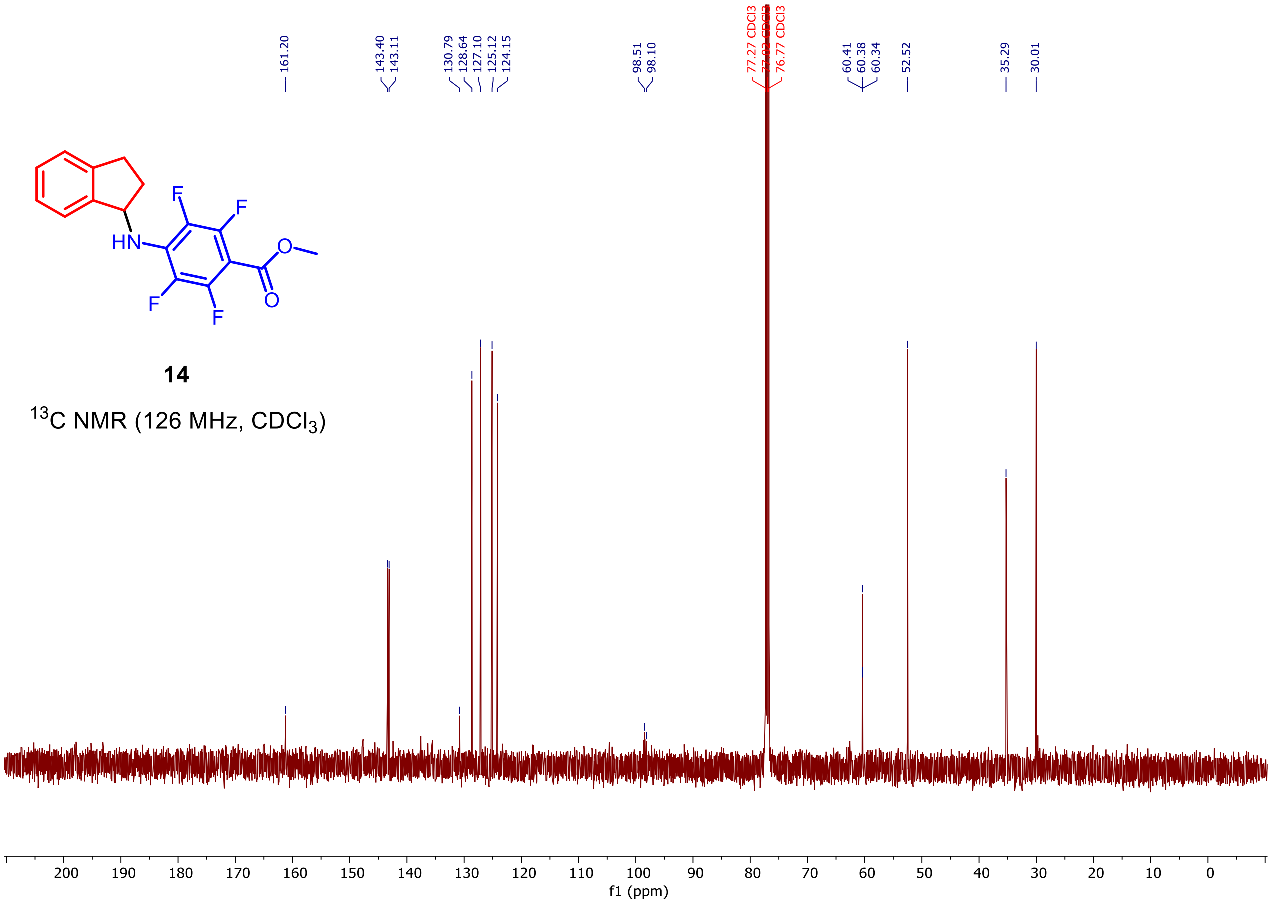

14

^{13}C NMR (126 MHz, $CDCl_3$)

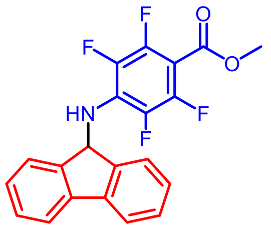

15

^{1}H NMR (500 MHz, $CDCl_3$)

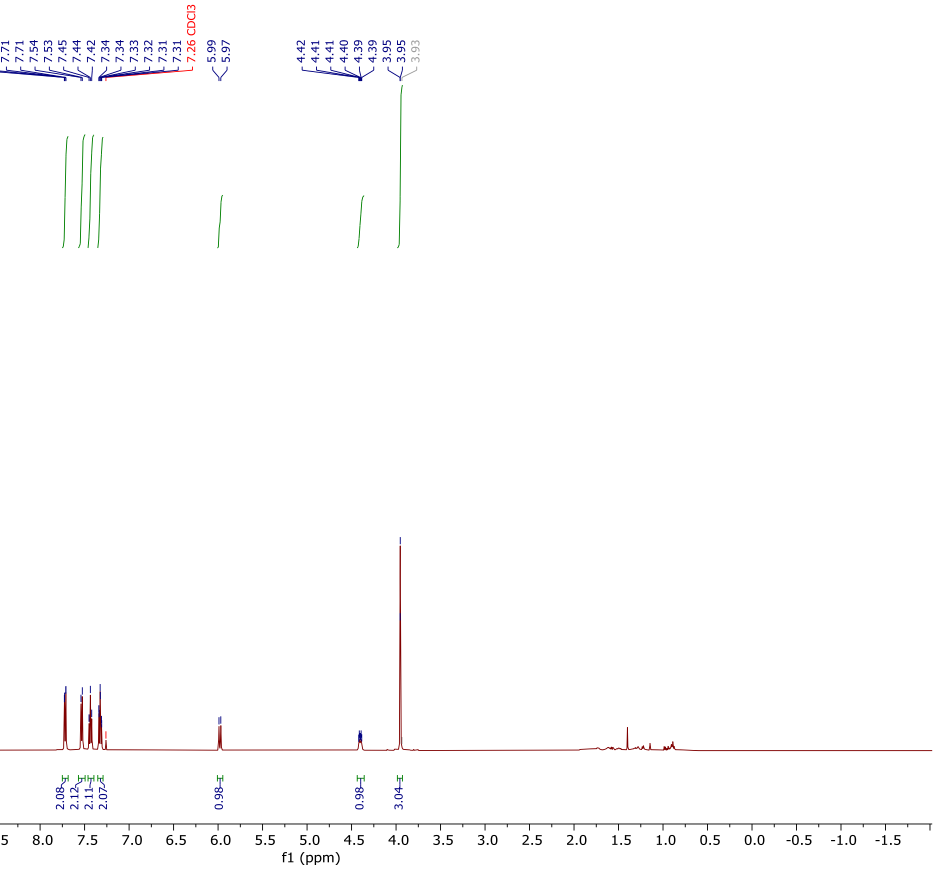

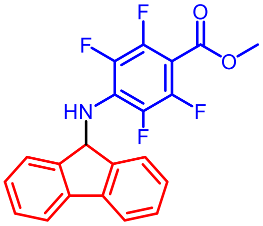

15

^{19}F NMR (471 MHz, $CDCl_3$)

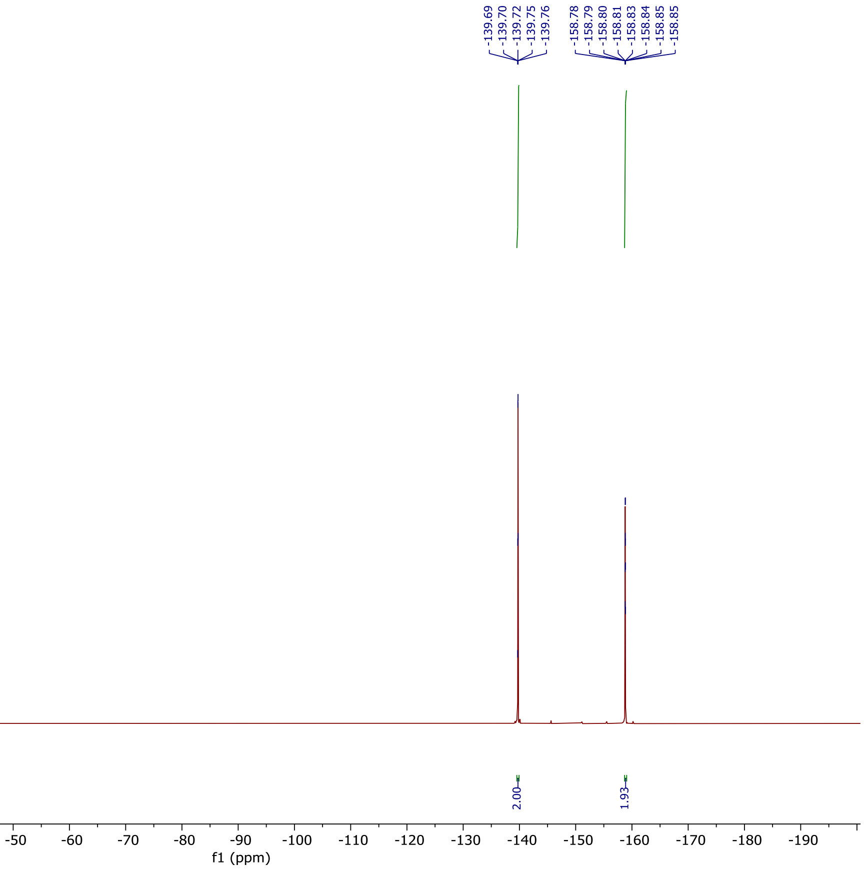

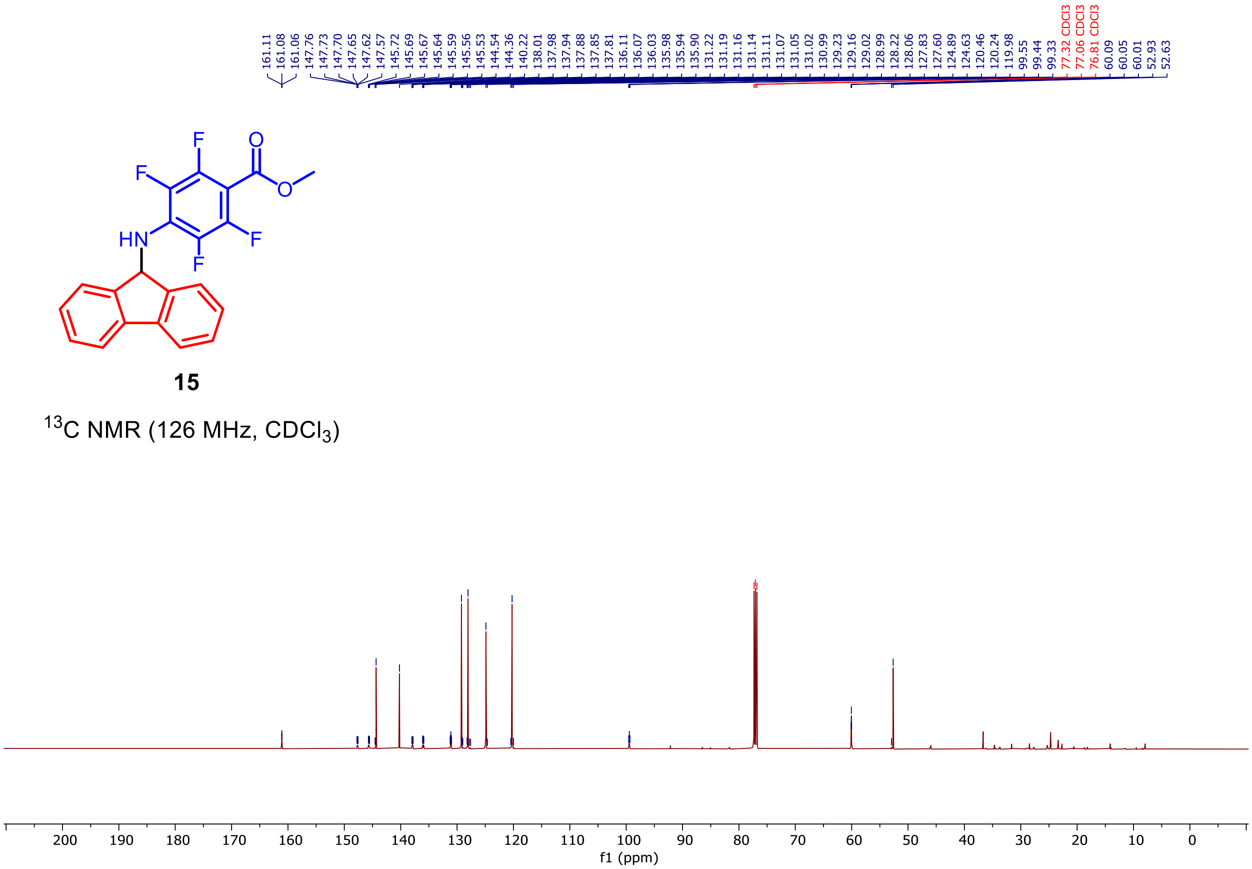

15

^{13}C NMR (126 MHz, $CDCl_3$)

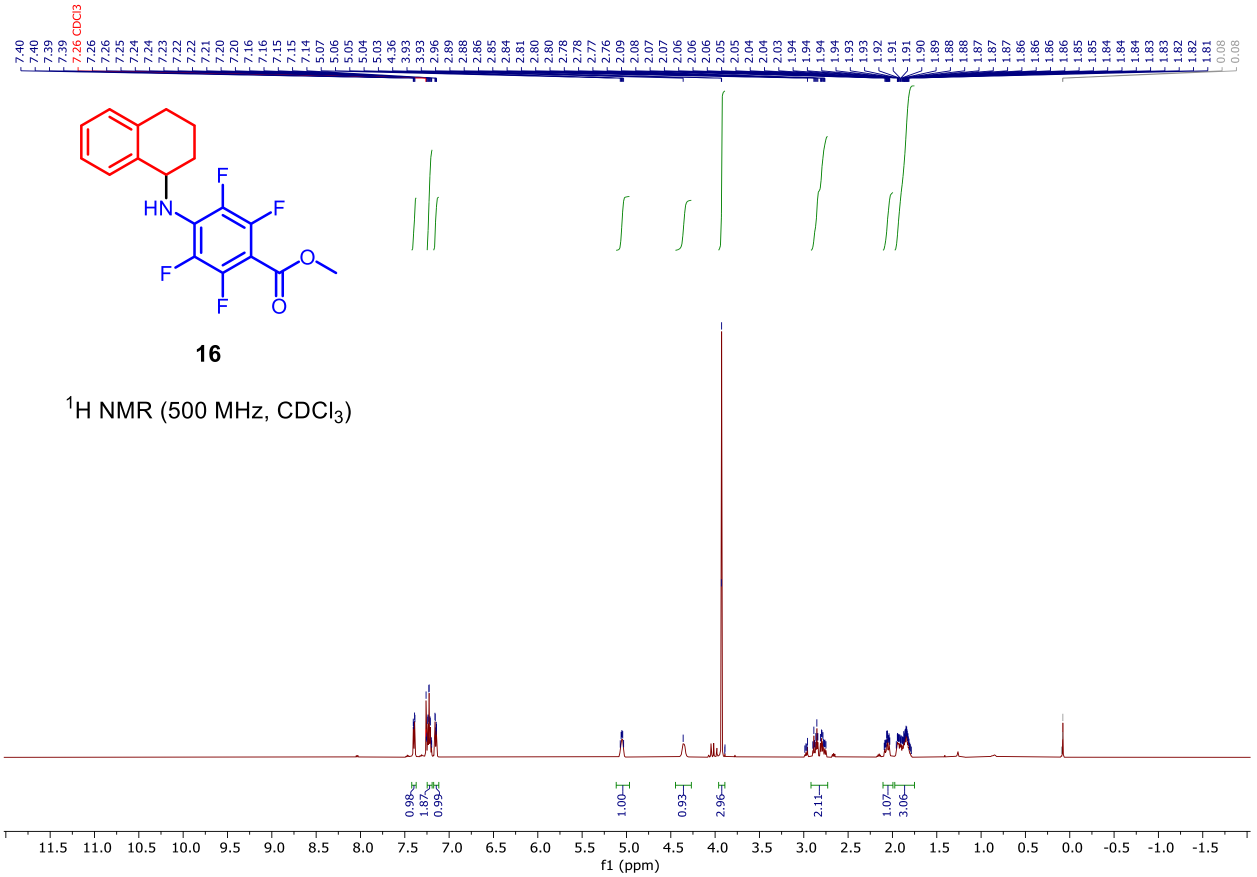

16

^{1}H NMR (500 MHz, $CDCl_3$)

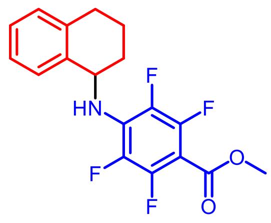

16

^{19}F NMR (471 MHz, $CDCl_3$)

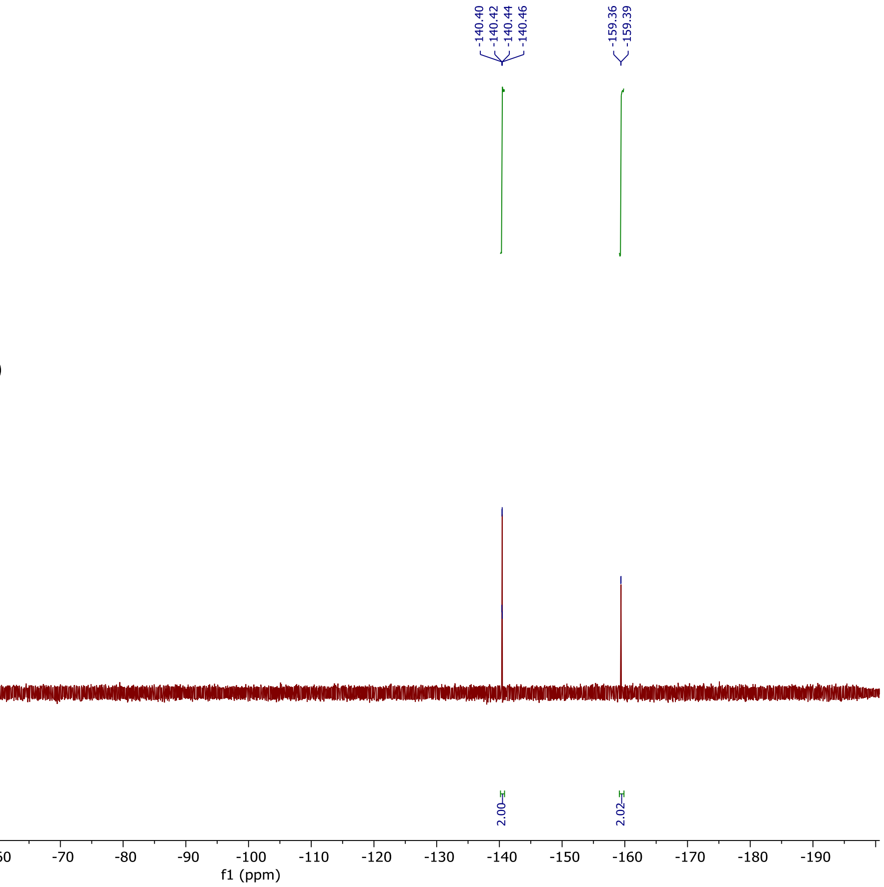

16

^{13}C NMR (126 MHz, $CDCl_3$)

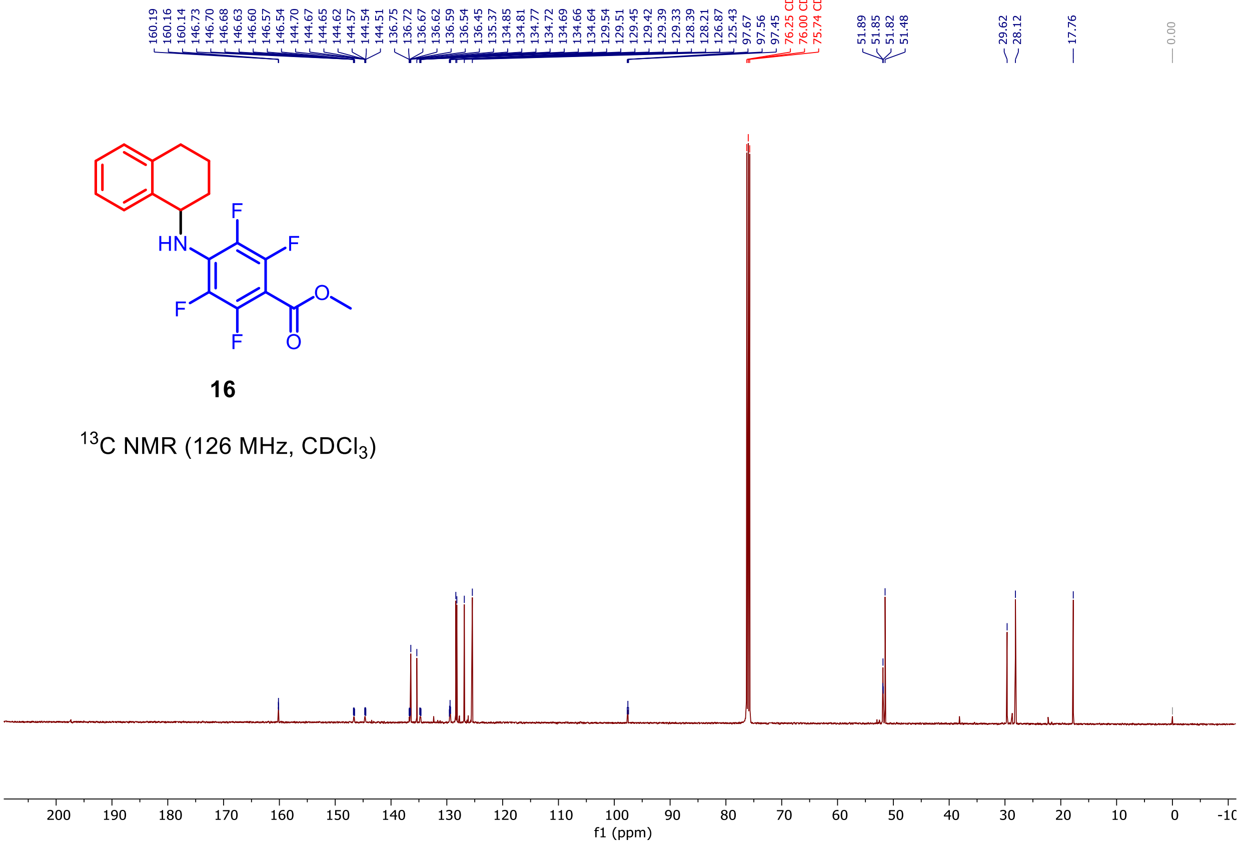

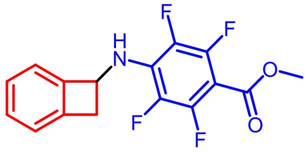

17

^{1}H NMR (500 MHz, $CDCl_3$)

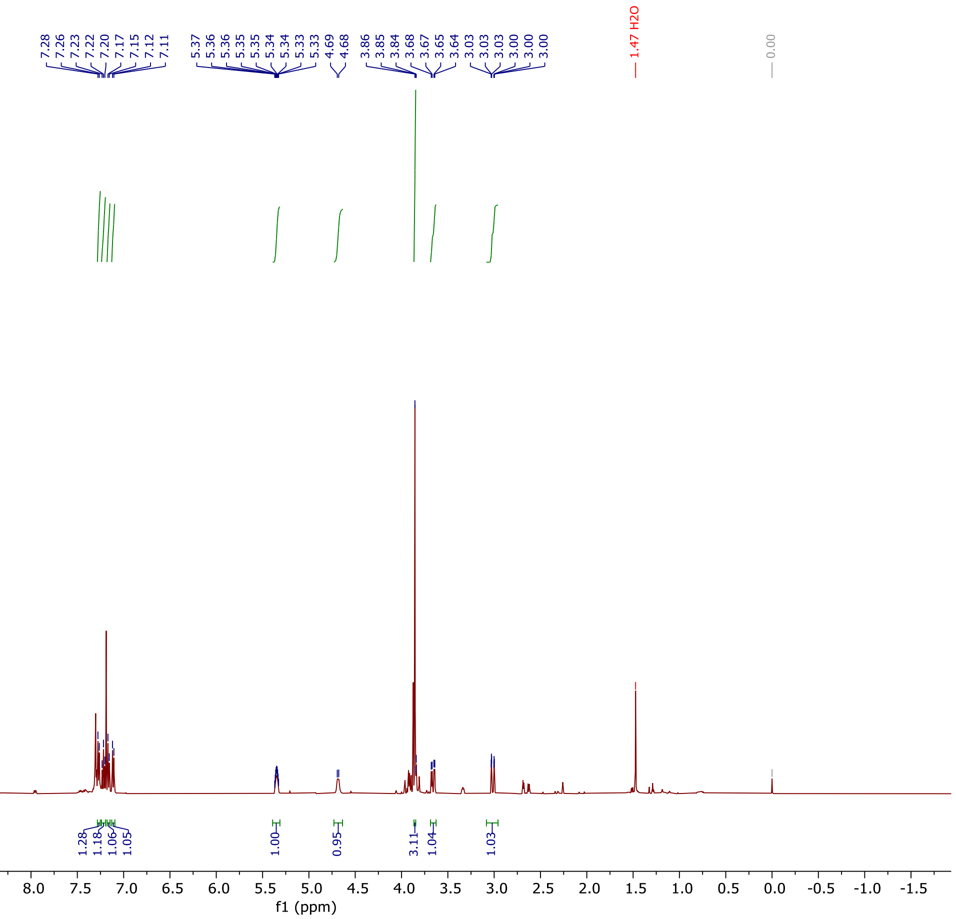

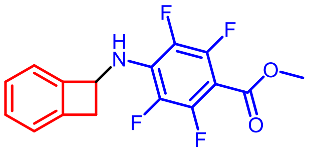

17

^{19}F NMR (471 MHz, $CDCl_3$)

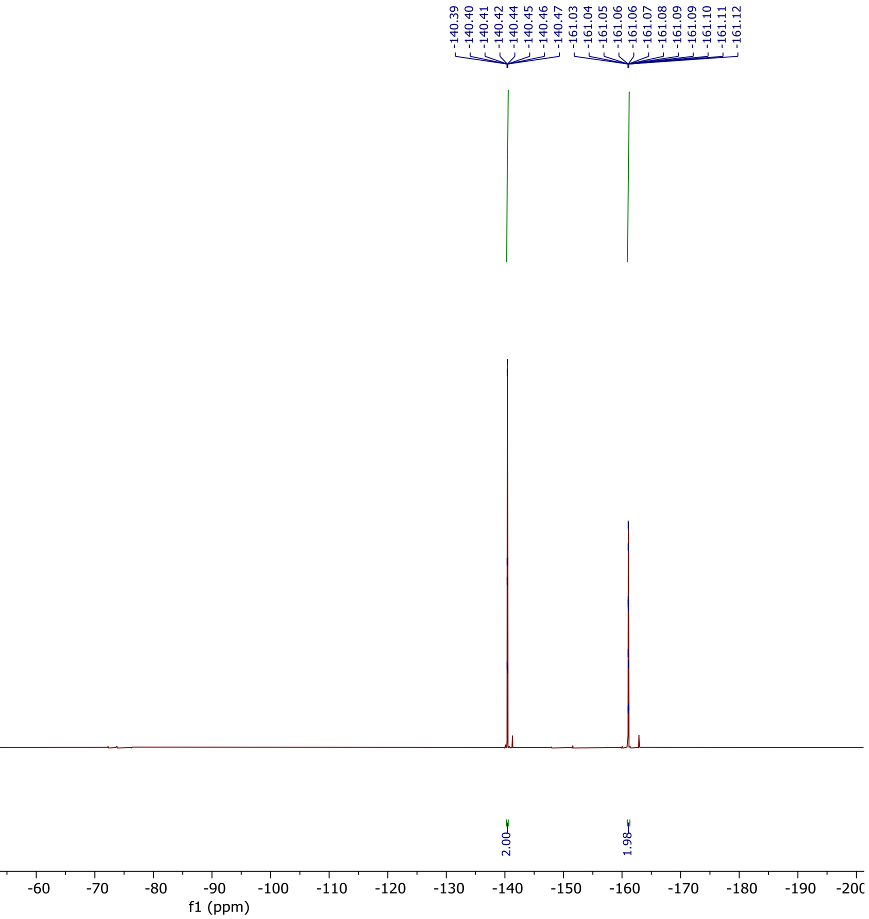

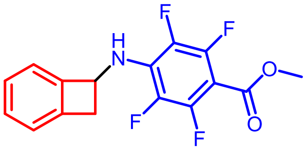

17

^{13}C NMR (126 MHz, $CDCl_3$)

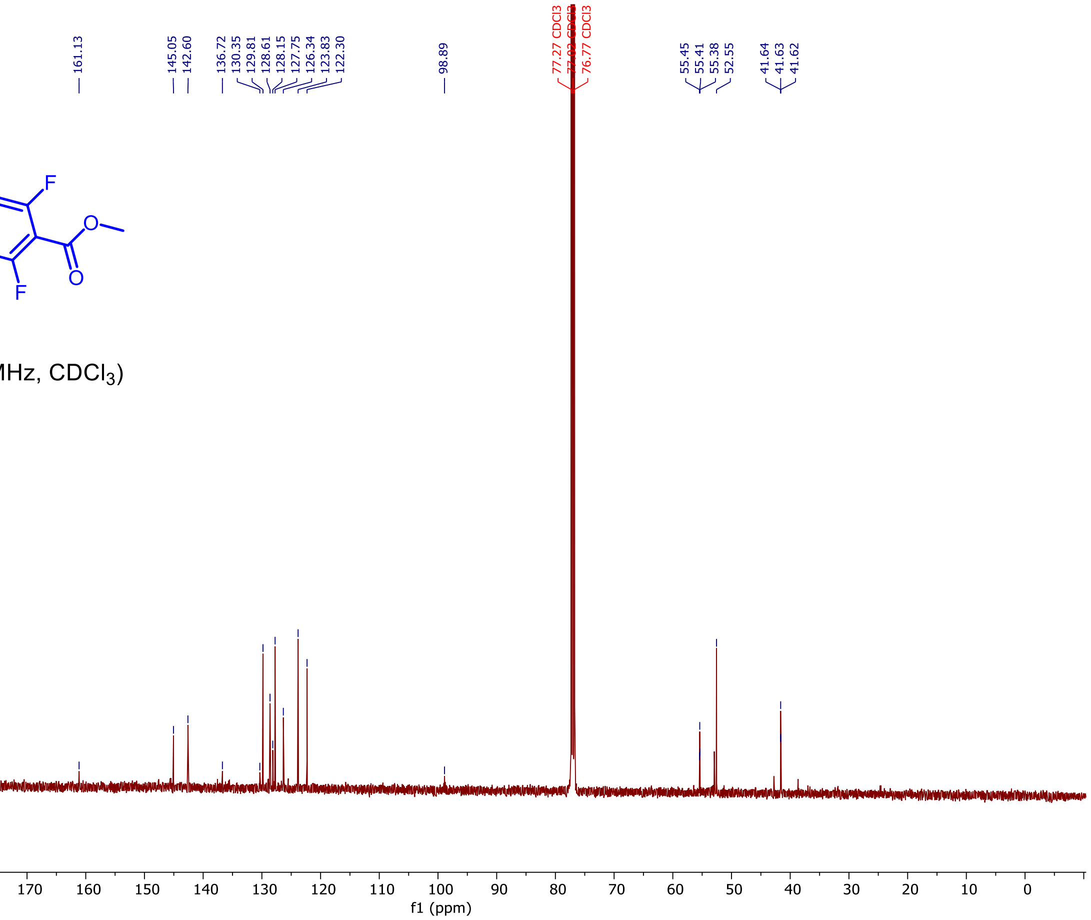

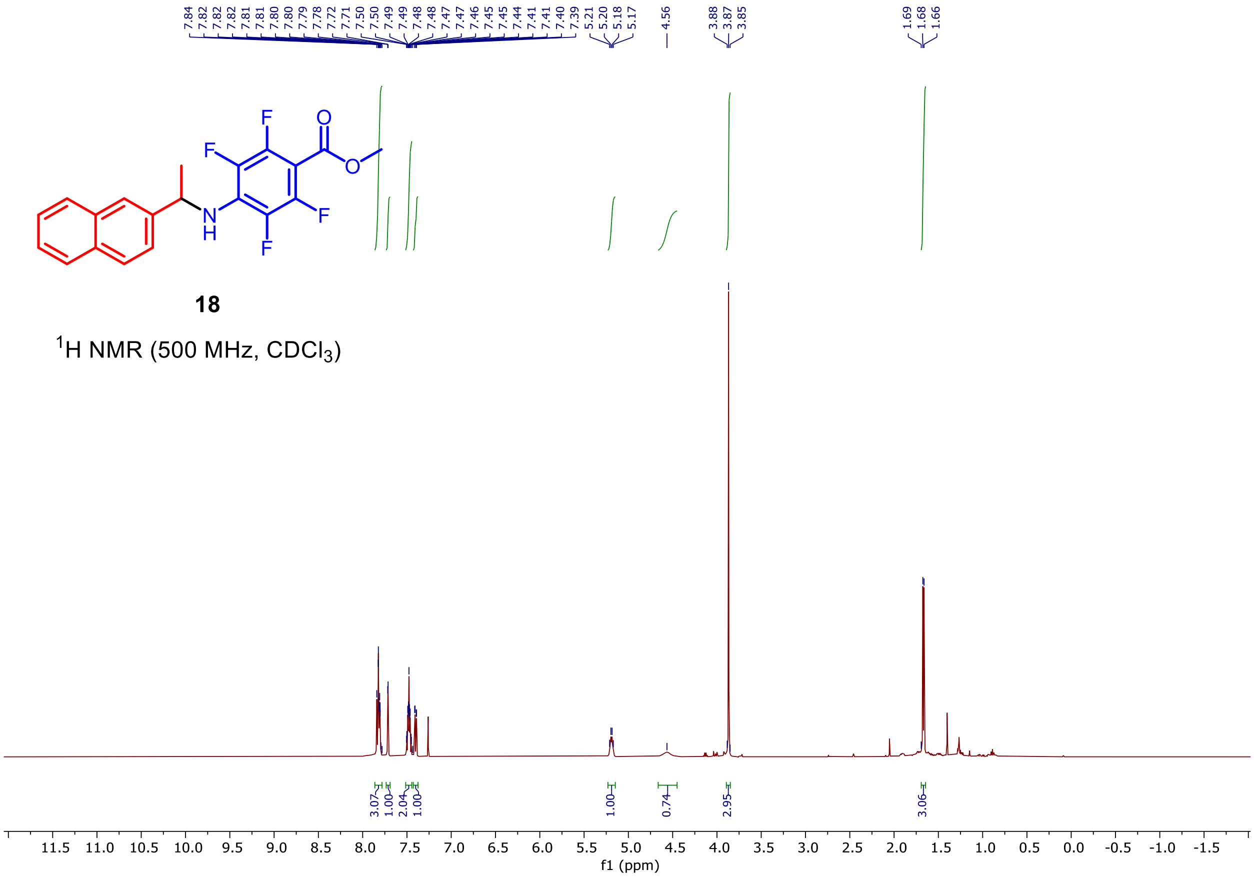

18

^1H NMR (500 MHz, $CDCl_3$)

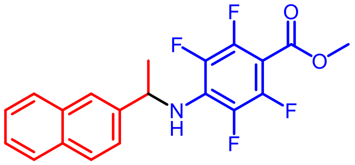

18

^{19}F NMR (471 MHz, $CDCl_3$)

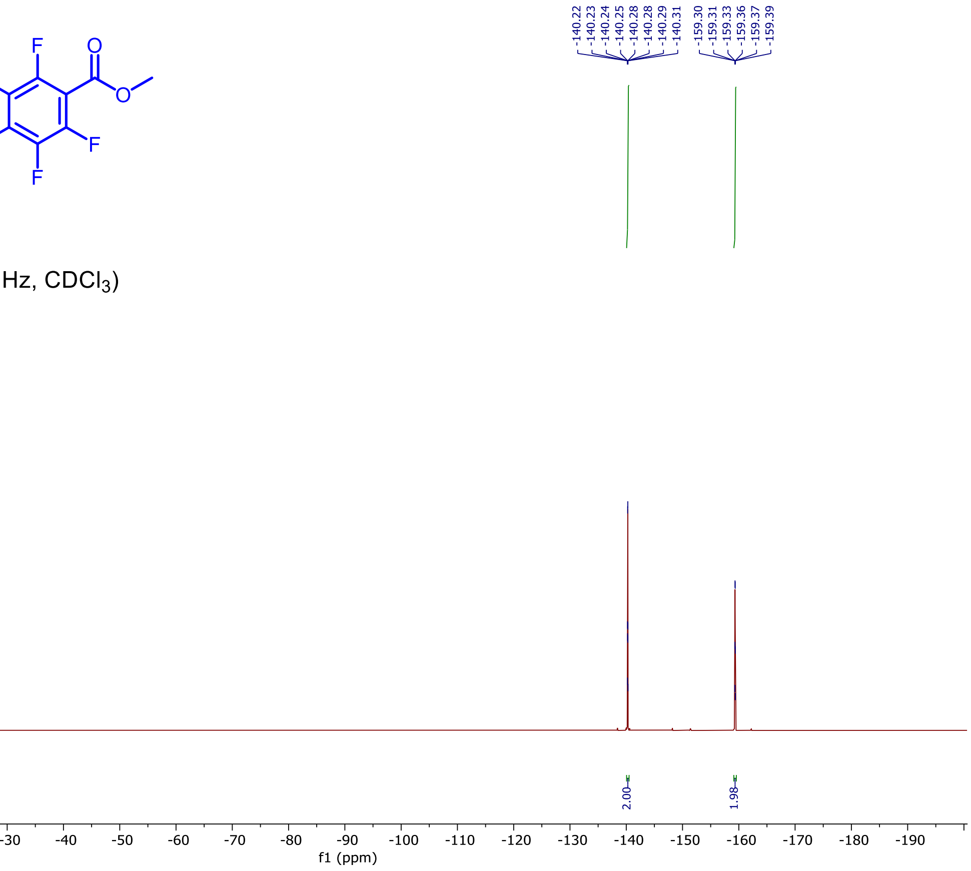

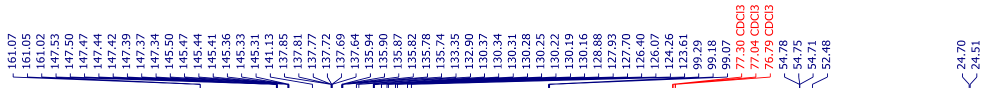

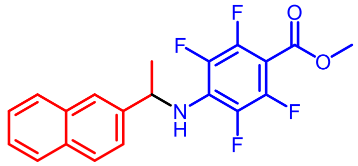

18

^{13}C NMR (126 MHz, $CDCl_3$)

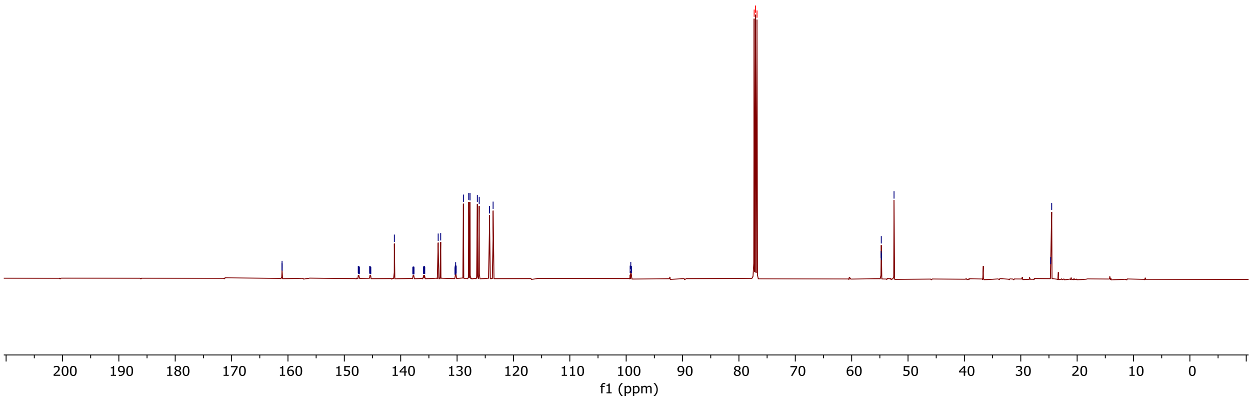

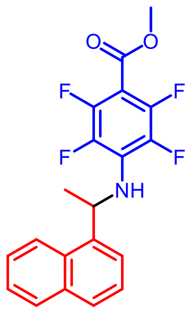

19

^{1}H NMR (500 MHz, $CDCl_3$)

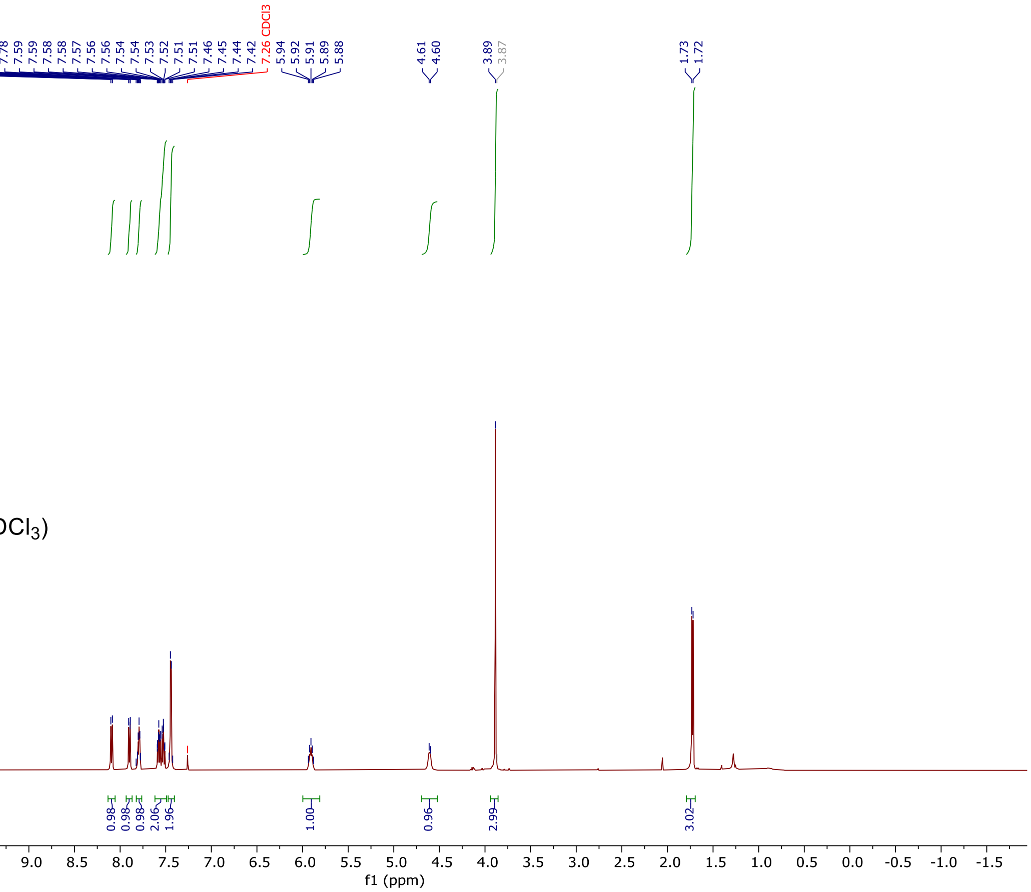

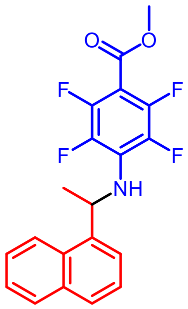

19

^{19}F NMR (471 MHz, $CDCl_3$)

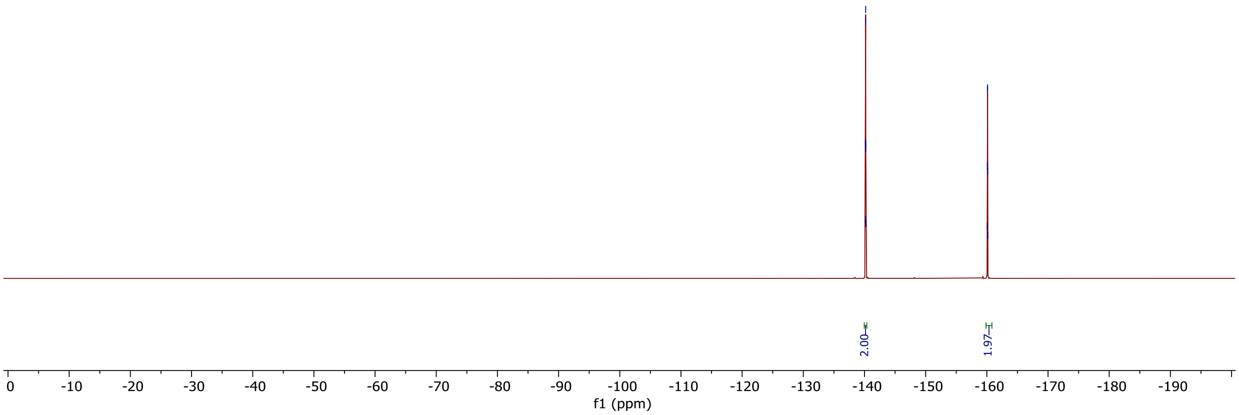

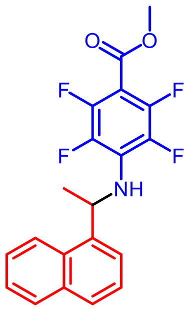

19

^{13}C NMR (126 MHz, $CDCl_3$)

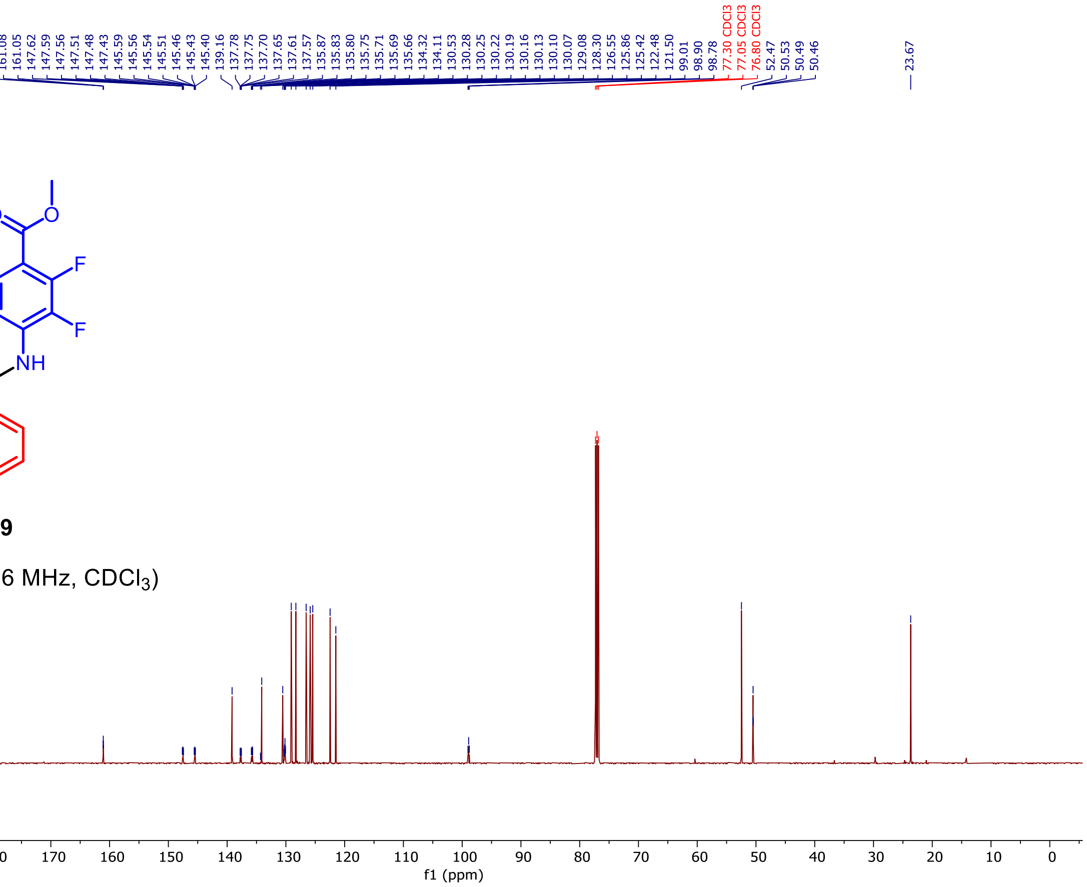

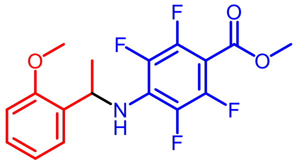

20

^{1}H NMR (500 MHz, $CDCl_3$)

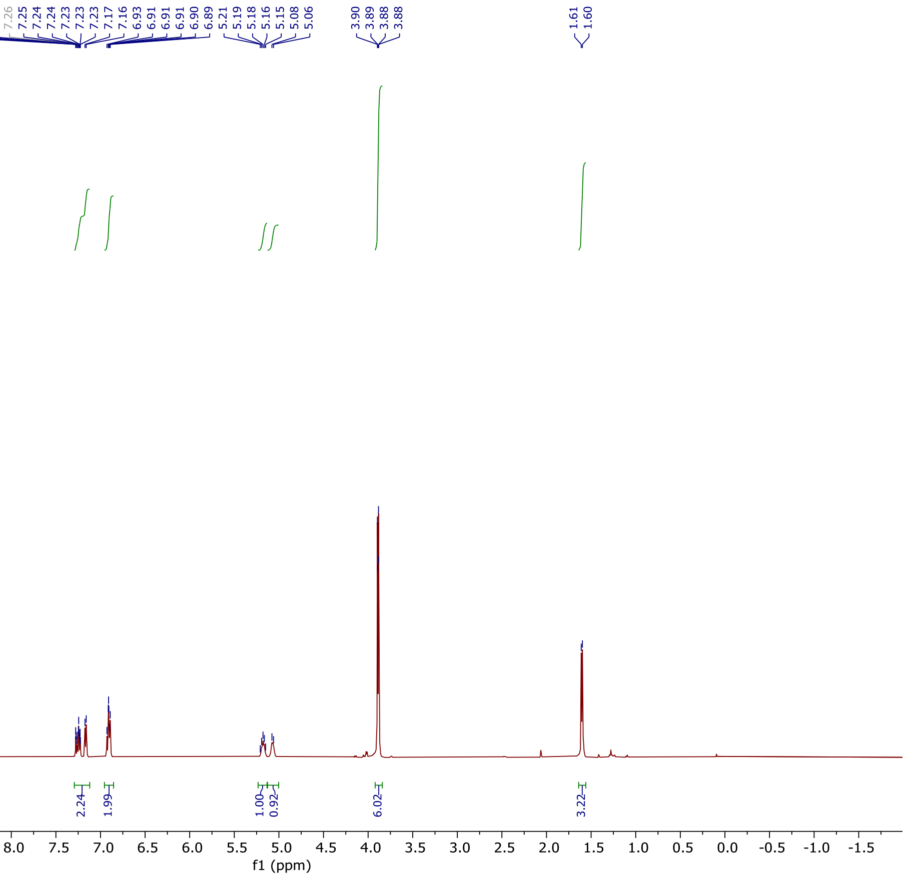

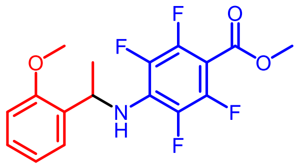

20

^{19}F NMR (471 MHz, $CDCl_3$)

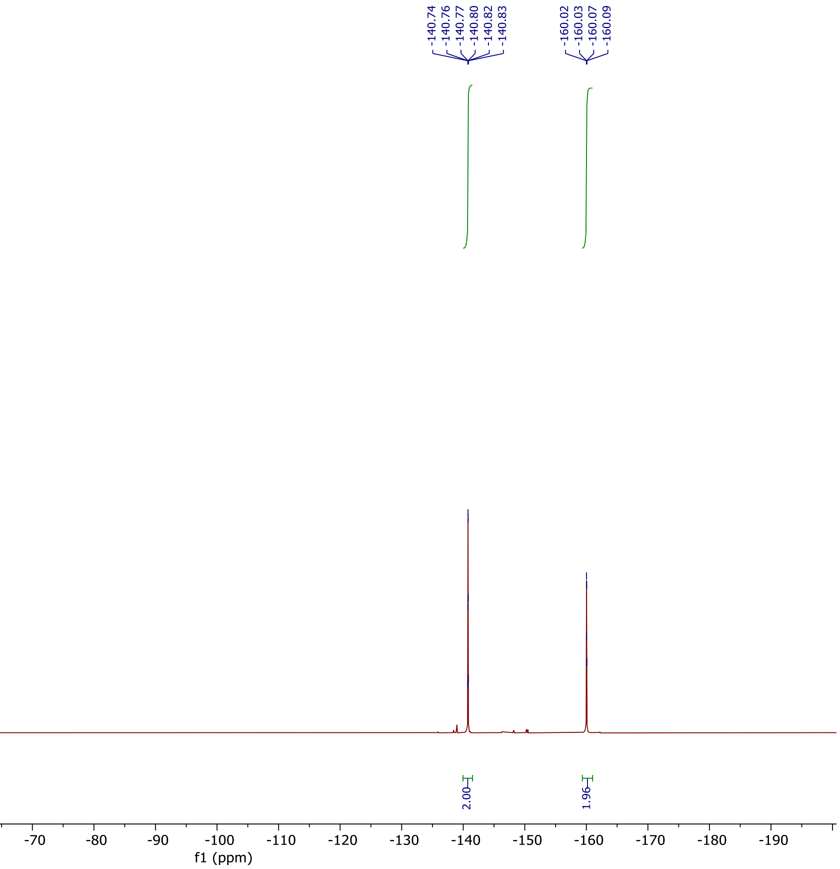

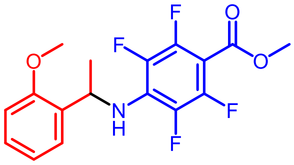

20

^{13}C NMR (126 MHz, $CDCl_3$)

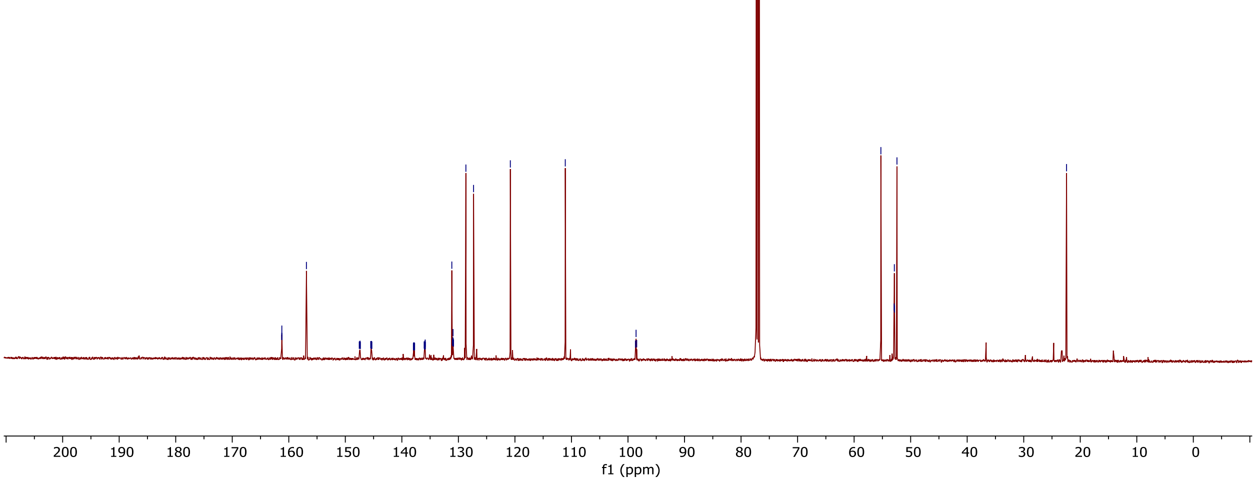

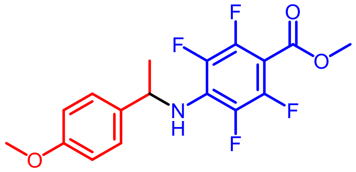

21

^1H NMR (500 MHz, $CDCl_3$)

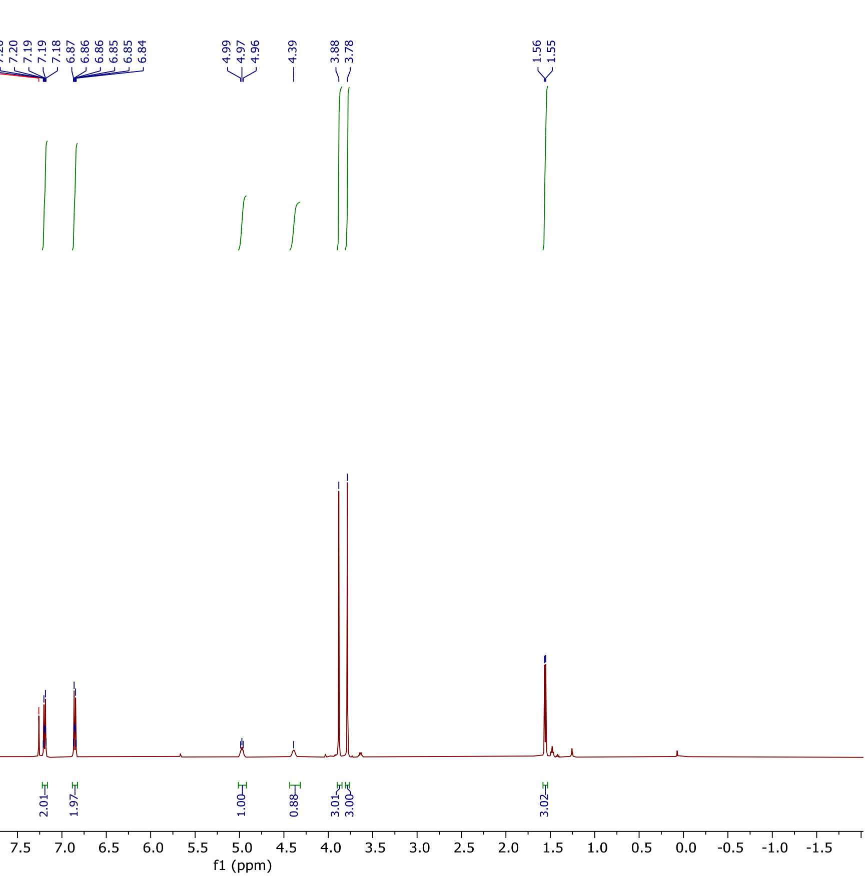

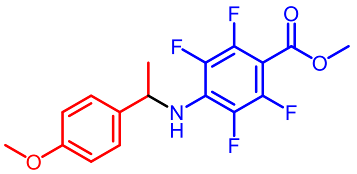

21

^{19}F NMR (471 MHz, $CDCl_3$)

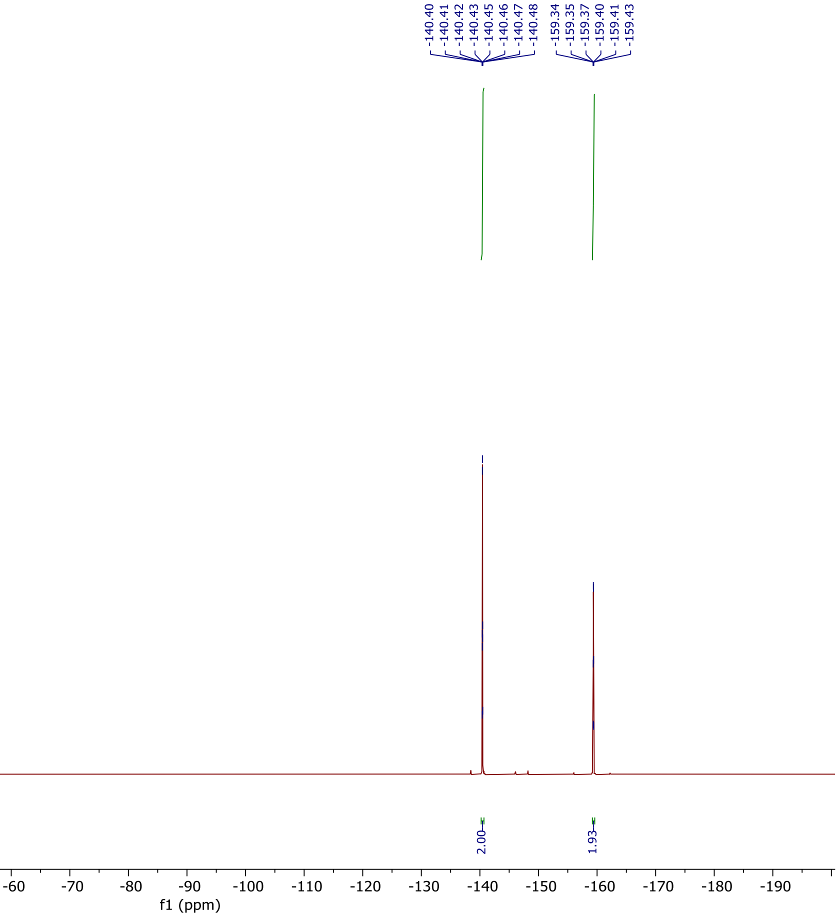

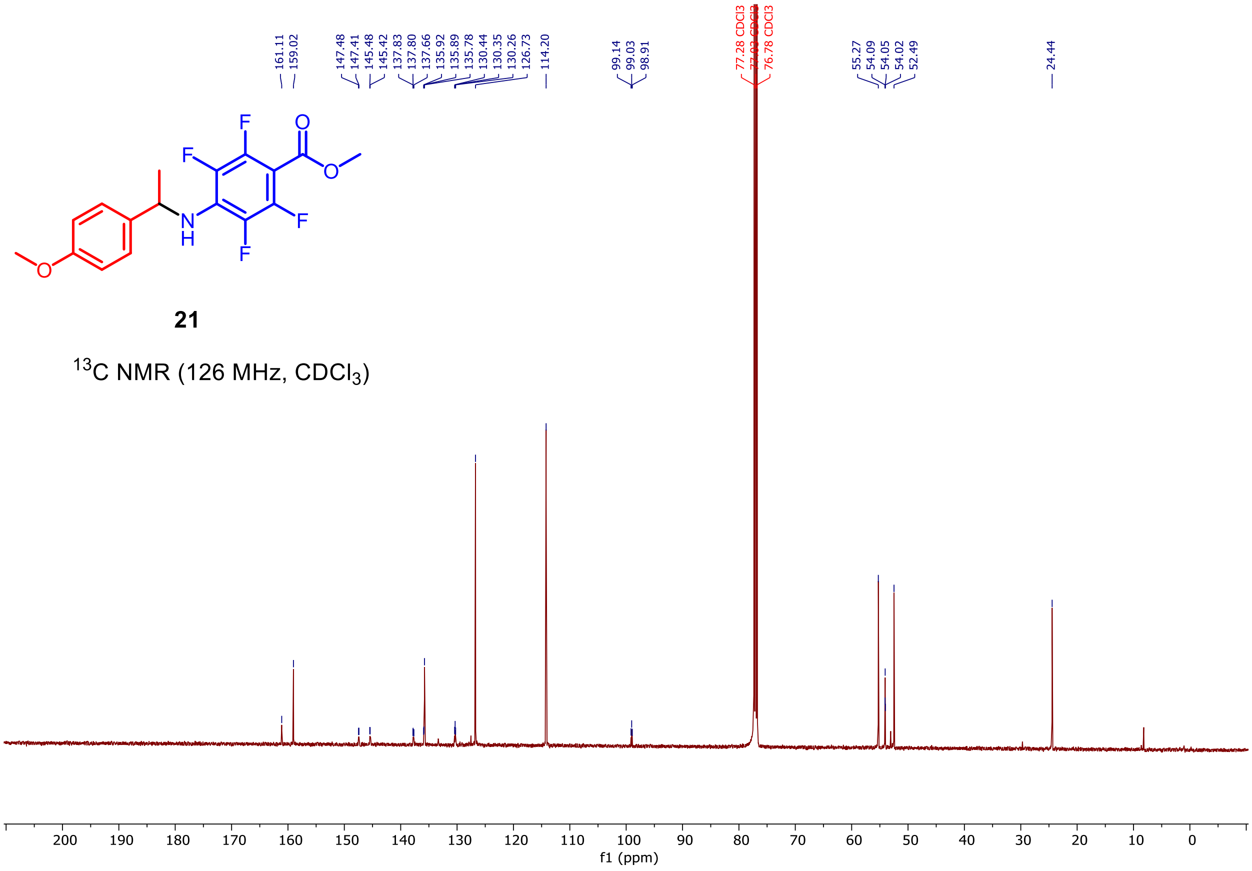

21

^{13}C NMR (126 MHz, $CDCl_3$)

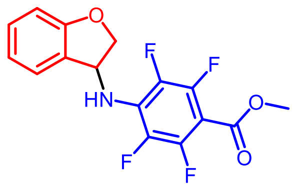

22

^{1}H NMR (500 MHz, $CDCl_3$)

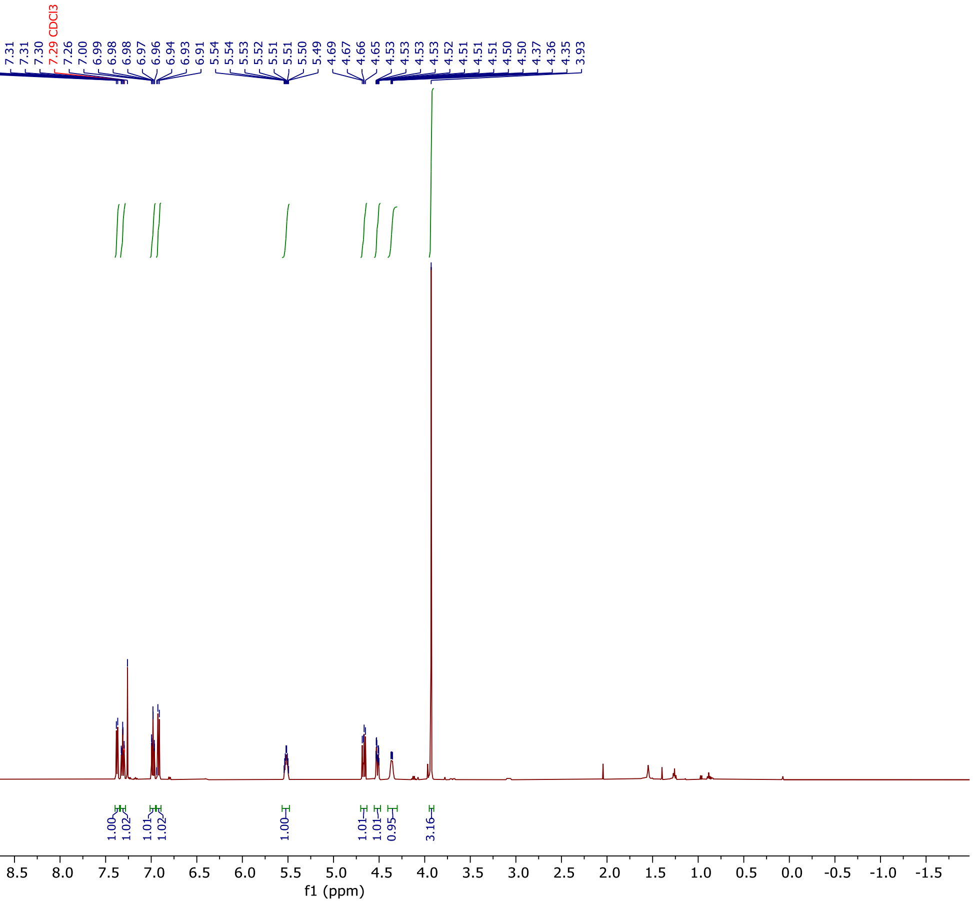

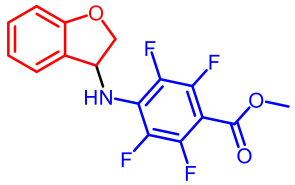

22

^{19}F NMR (471 MHz, $CDCl_3$)

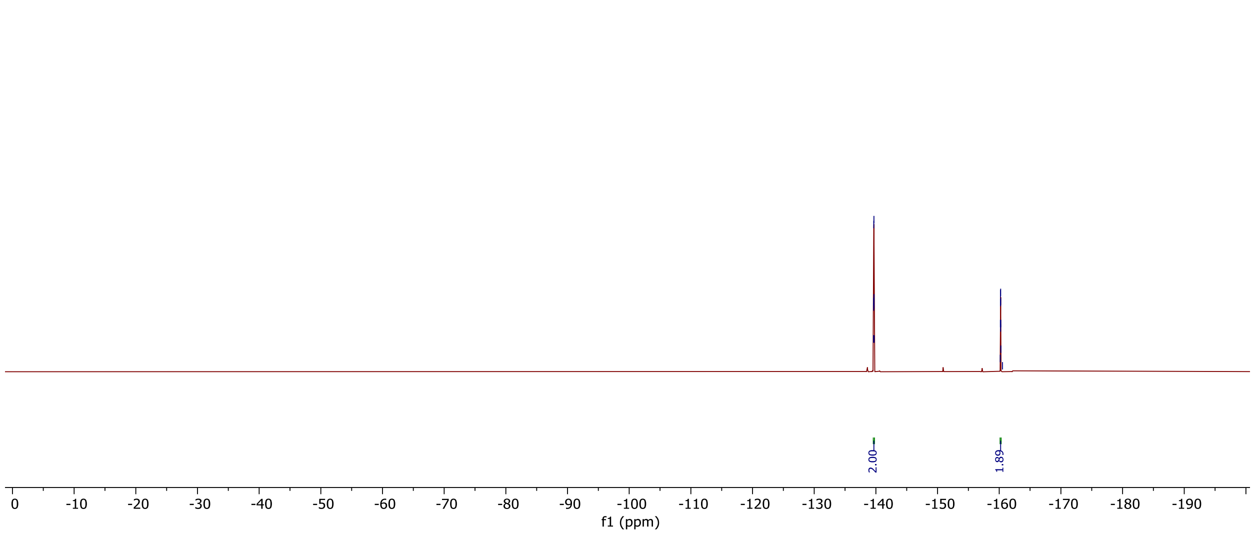

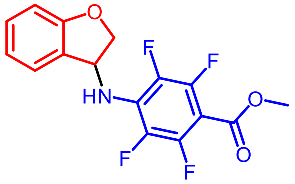

22

^{13}C NMR (126 MHz, $CDCl_3$)

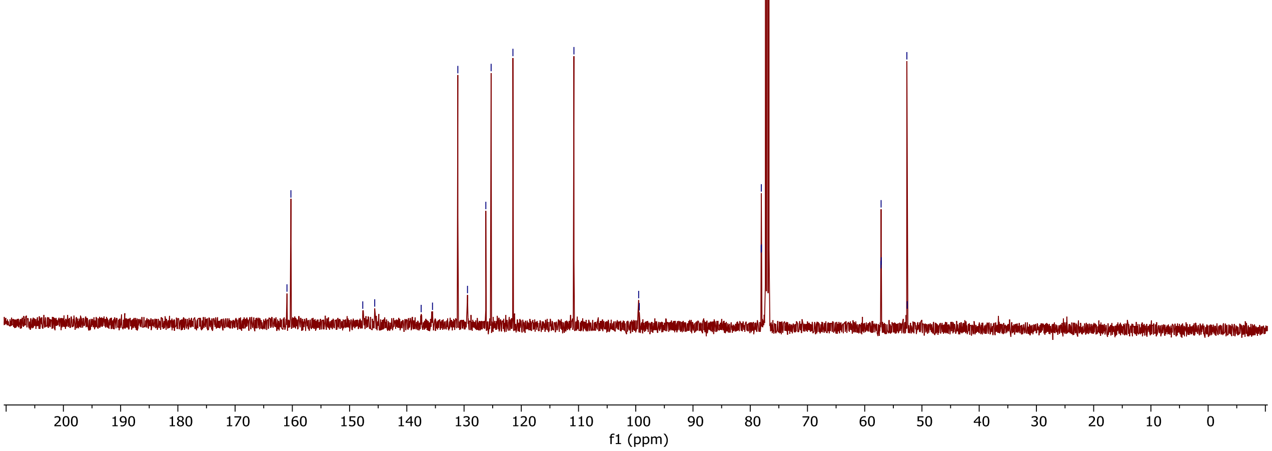

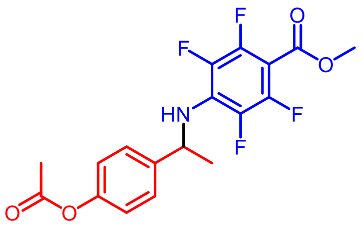

23

^{1}H NMR (500 MHz, $CDCl_3$)

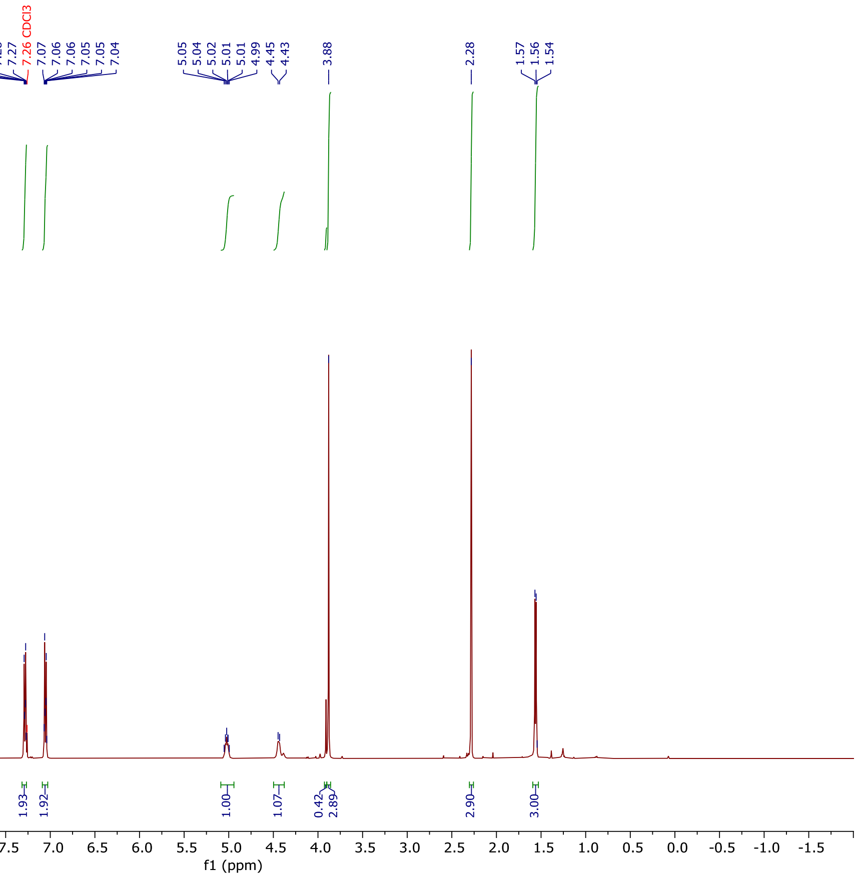

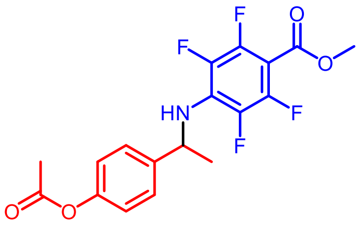

23

^{19}F NMR (471 MHz, $CDCl_3$)

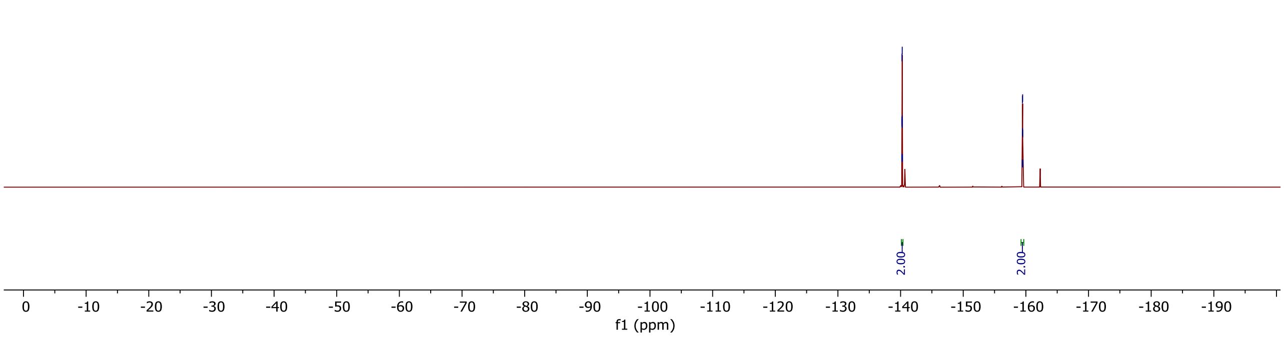

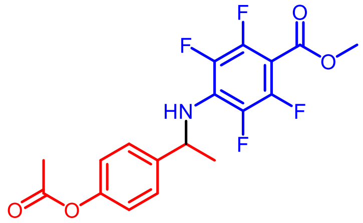

23

^{13}C NMR (126 MHz, $CDCl_3$)

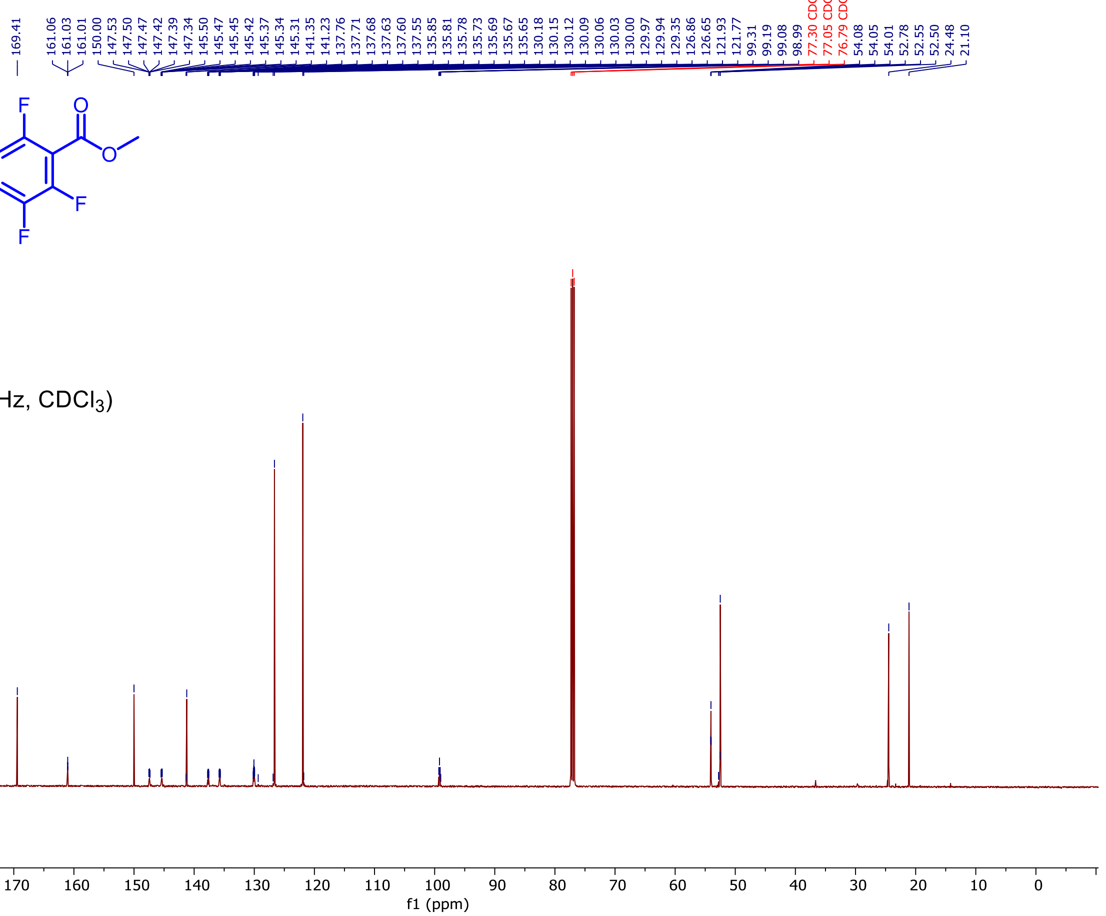

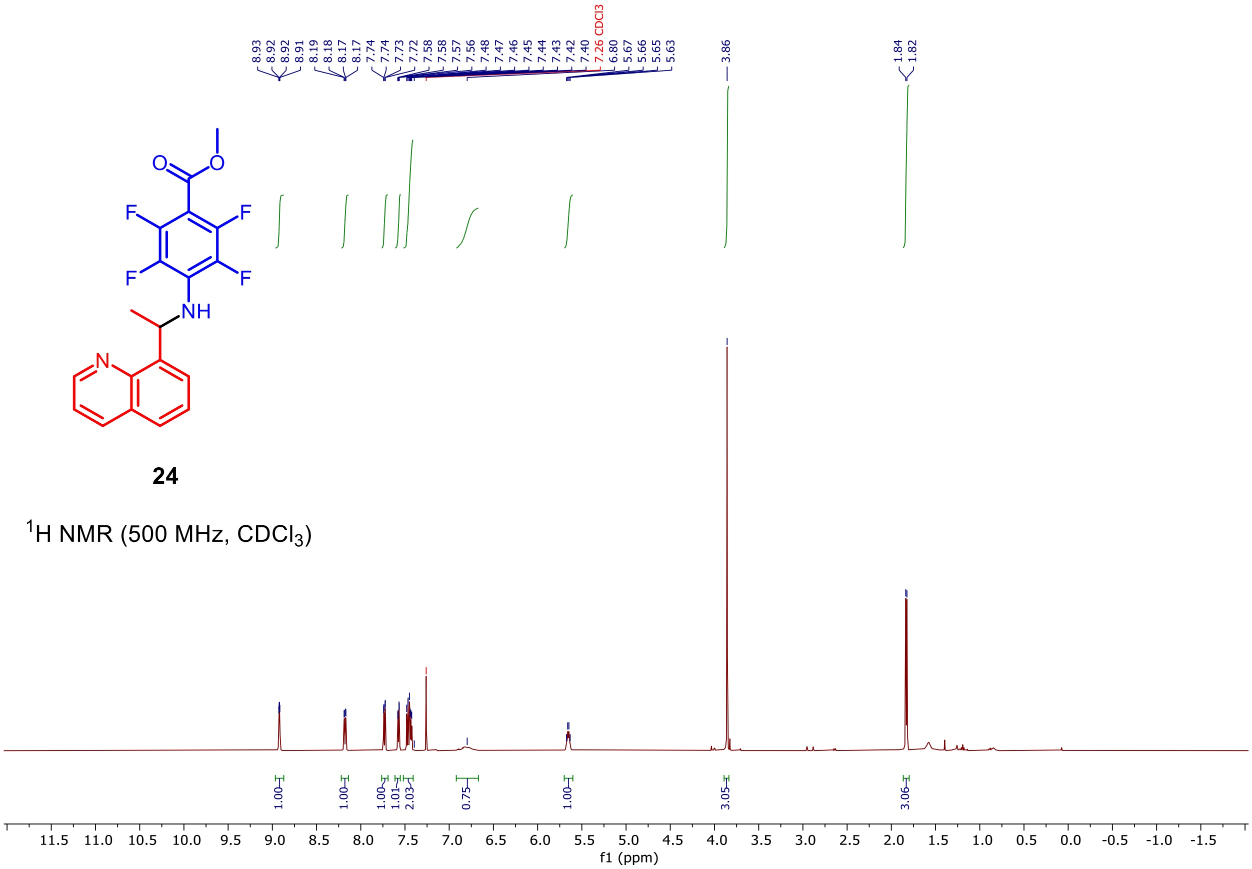

24
^{1}H NMR (500 MHz, CDCl$_3$)
8.93
8.92
8.92
8.91
8.19
8.18
8.17
8.17
7.74
7.74
7.73
7.72
7.58
7.58
7.57
7.56
7.48
7.47
7.46
7.45
7.44
7.43
7.42
7.40
7.26 CDCl3
6.80
5.67
5.66
5.65
5.63
3.86
1.84
1.82
1.00
1.00
1.00
1.01
2.03
0.75
1.00
3.05
3.06
f1 (ppm)

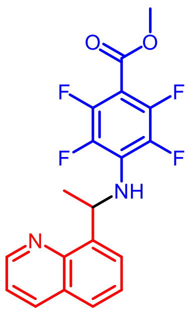

24

^{19}F NMR (471 MHz, $CDCl_3$)

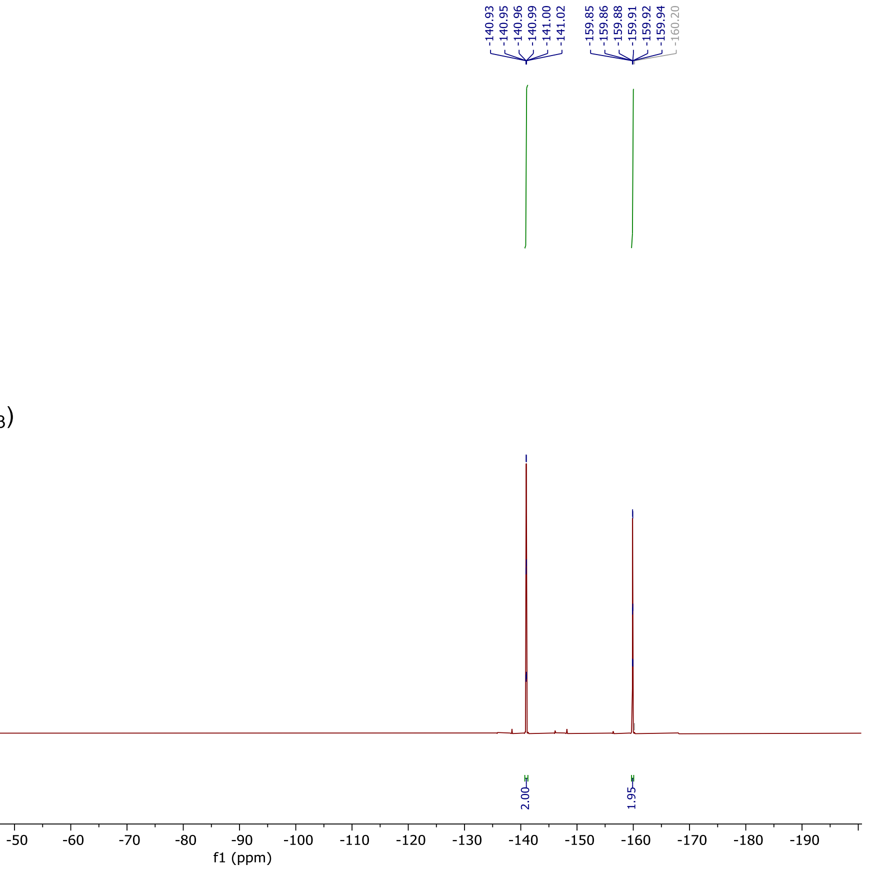

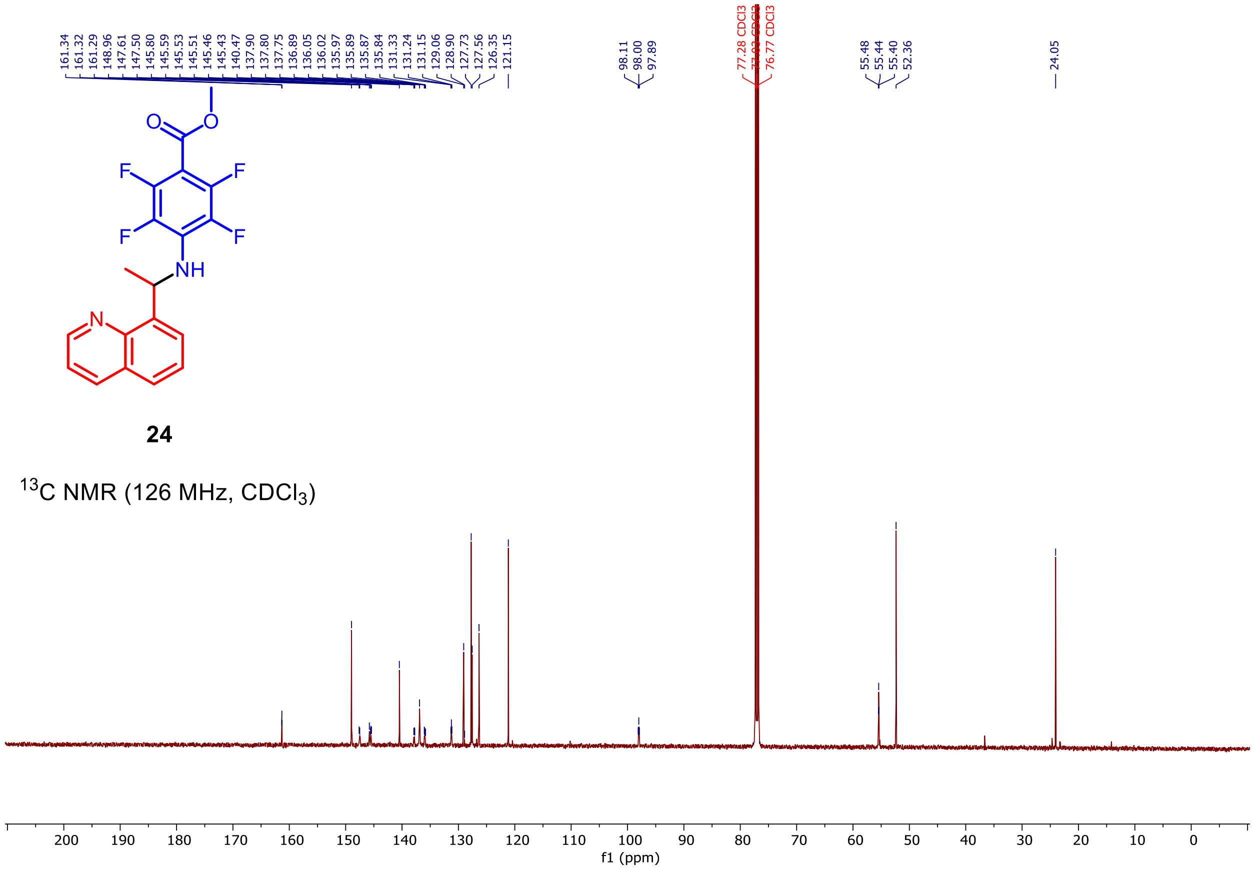

161.34
161.32
161.29
148.96
147.61
147.50
145.80
145.59
145.53
145.51
145.46
145.43
140.47
137.90
137.80
137.75
136.89
136.05
136.02
135.97
135.89
135.87
135.84
131.33
131.24
131.15
129.06
128.90
127.73
127.56
126.35
121.15
98.11
98.00
97.89
77.28 CDCl3
76.77 CDCl3
55.48
55.44
55.40
52.36
24.05
O
O
F
F
F
F
NH
N
24
^{13}C NMR (126 MHz, CDCl$_3$)
200 190 180 170 160 150 140 130 120 110 100 90 80 70 60 50 40 30 20 10 0
f1 (ppm)

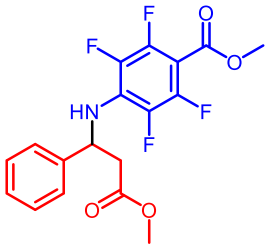

25

^{1}H NMR (500 MHz, $CDCl_3$)

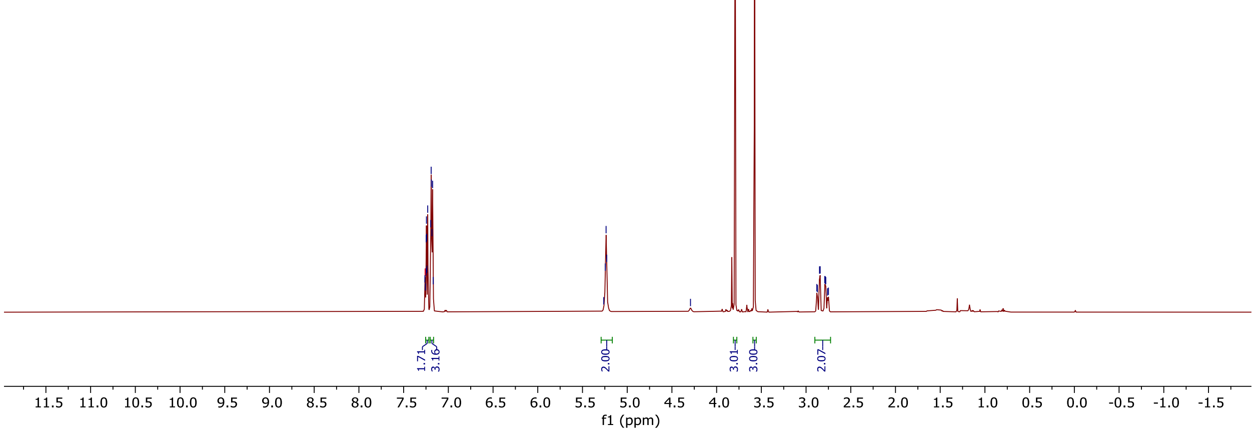

0+

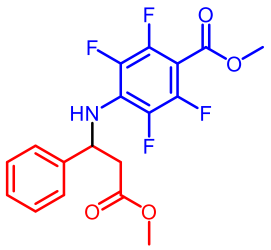

25

^{19}F NMR (471 MHz, $CDCl_3$)

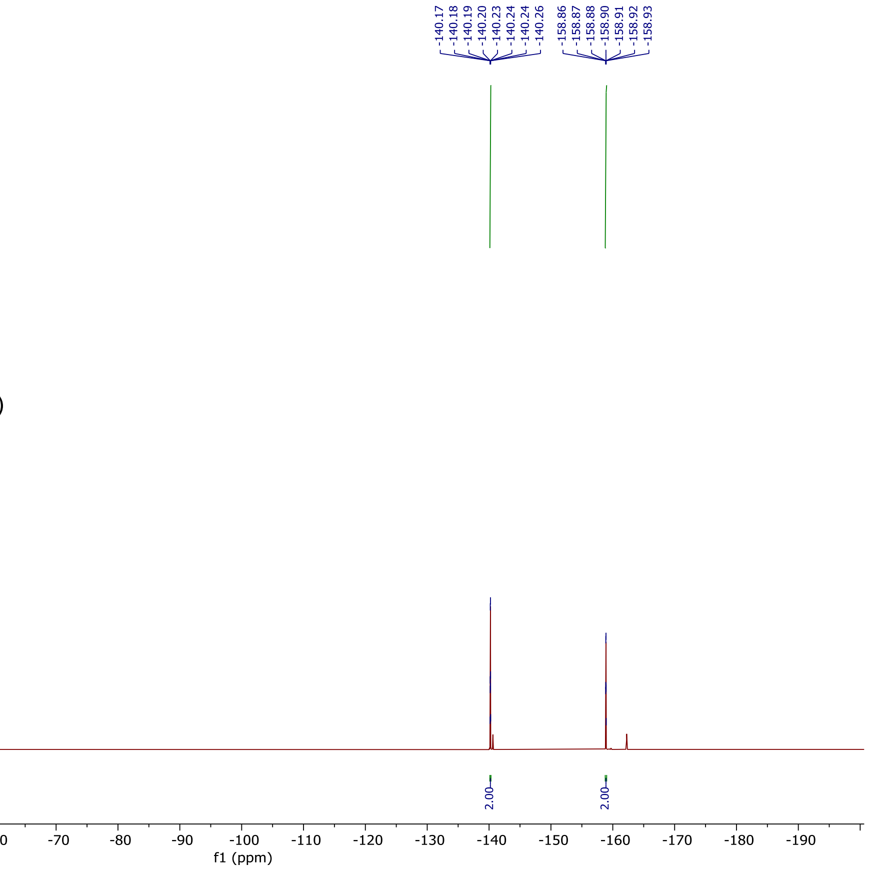

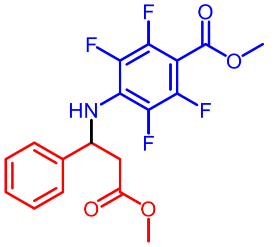

25

^{13}C NMR (126 MHz, $CDCl_3$)

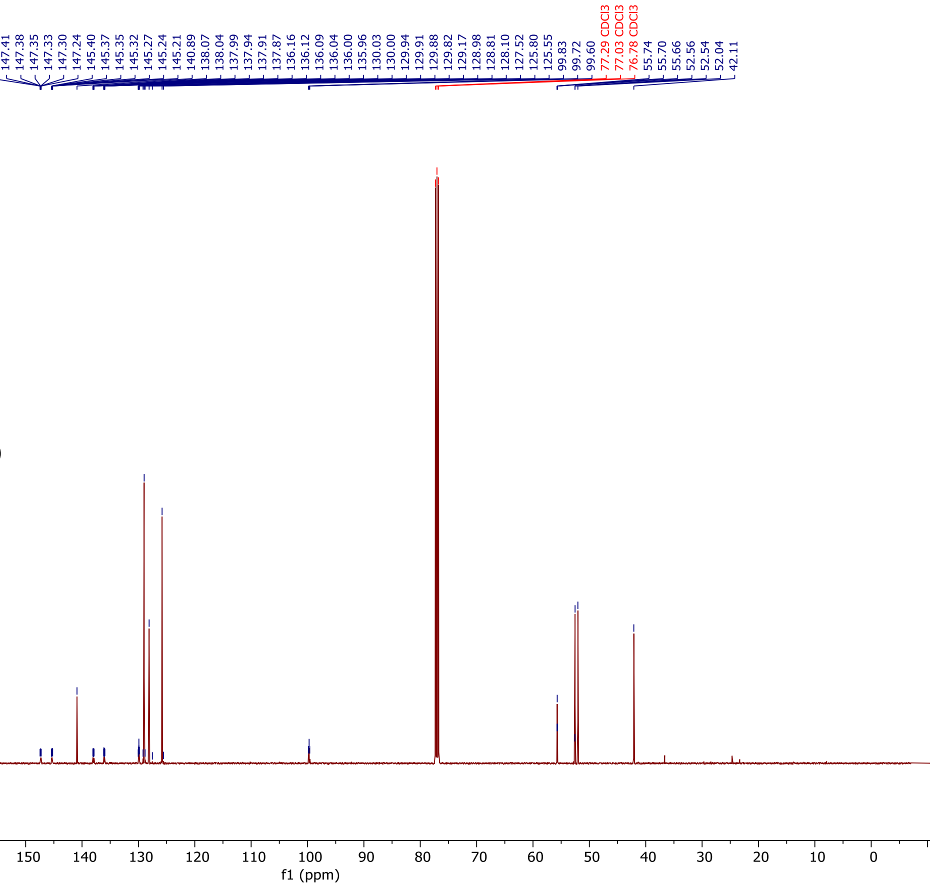

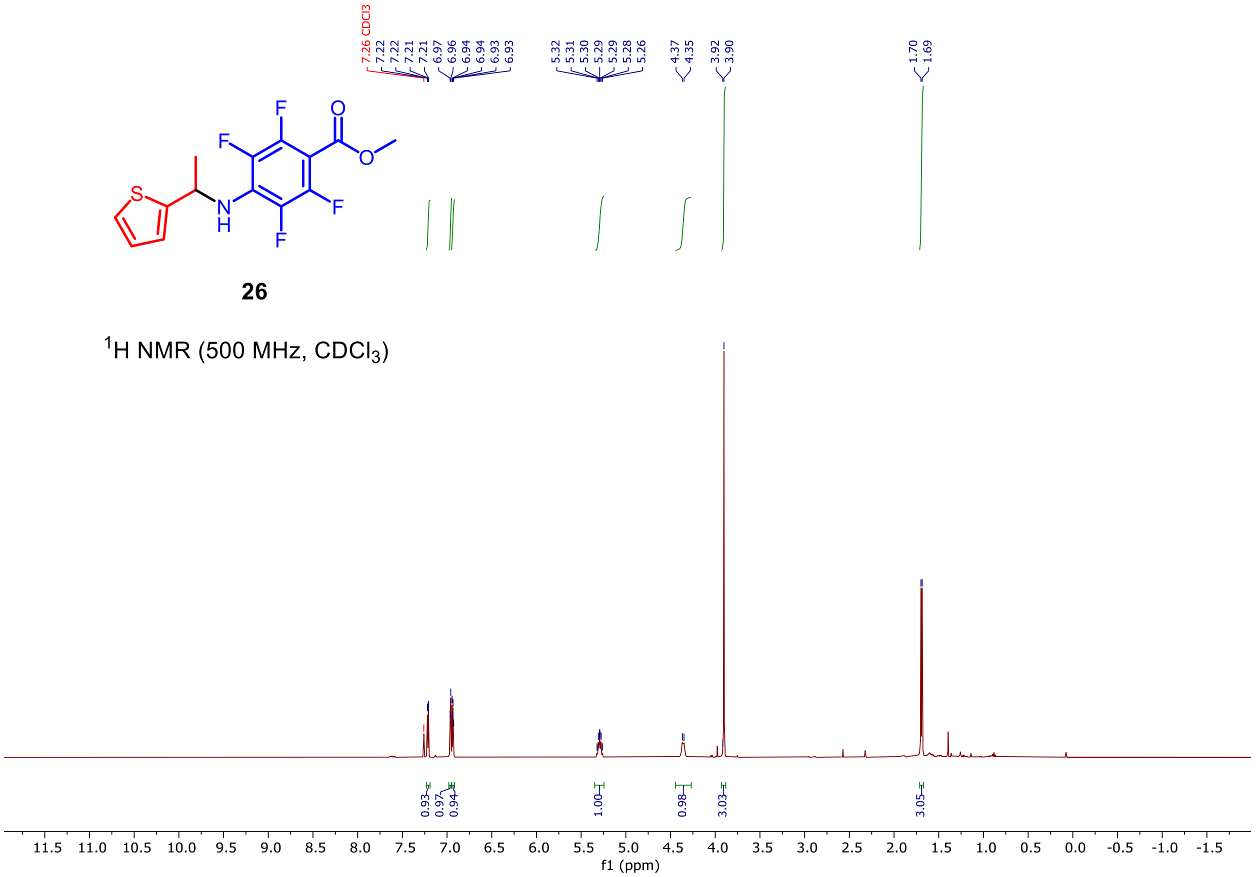
7.26 CDCl3
7.22
7.22
7.21
7.21
6.97
6.96
6.94
6.94
6.93
6.93
5.32
5.31
5.30
5.29
5.29
5.28
5.26
4.37
4.35
3.92
3.90
1.70
1.69
26
^{1}H NMR (500 MHz, $CDCl_3$)
0.93
0.97
0.94
1.00
0.98
3.03
3.05
11.5 11.0 10.5 10.0 9.5 9.0 8.5 8.0 7.5 7.0 6.5 6.0 5.5 5.0 4.5 4.0 3.5 3.0 2.5 2.0 1.5 1.0 0.5 0.0 -0.5 -1.0 -1.5
f1 (ppm)

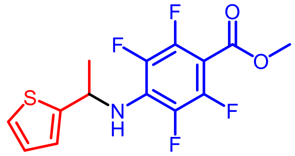

26

^{19}F NMR (471 MHz, $CDCl_3$)

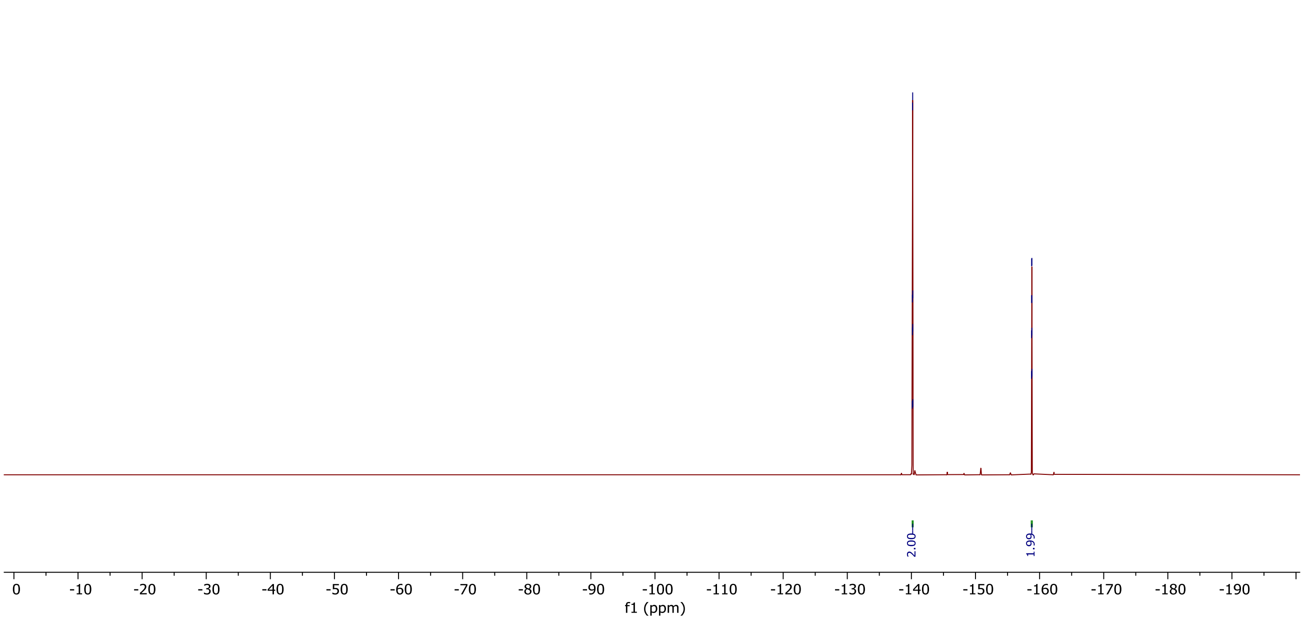

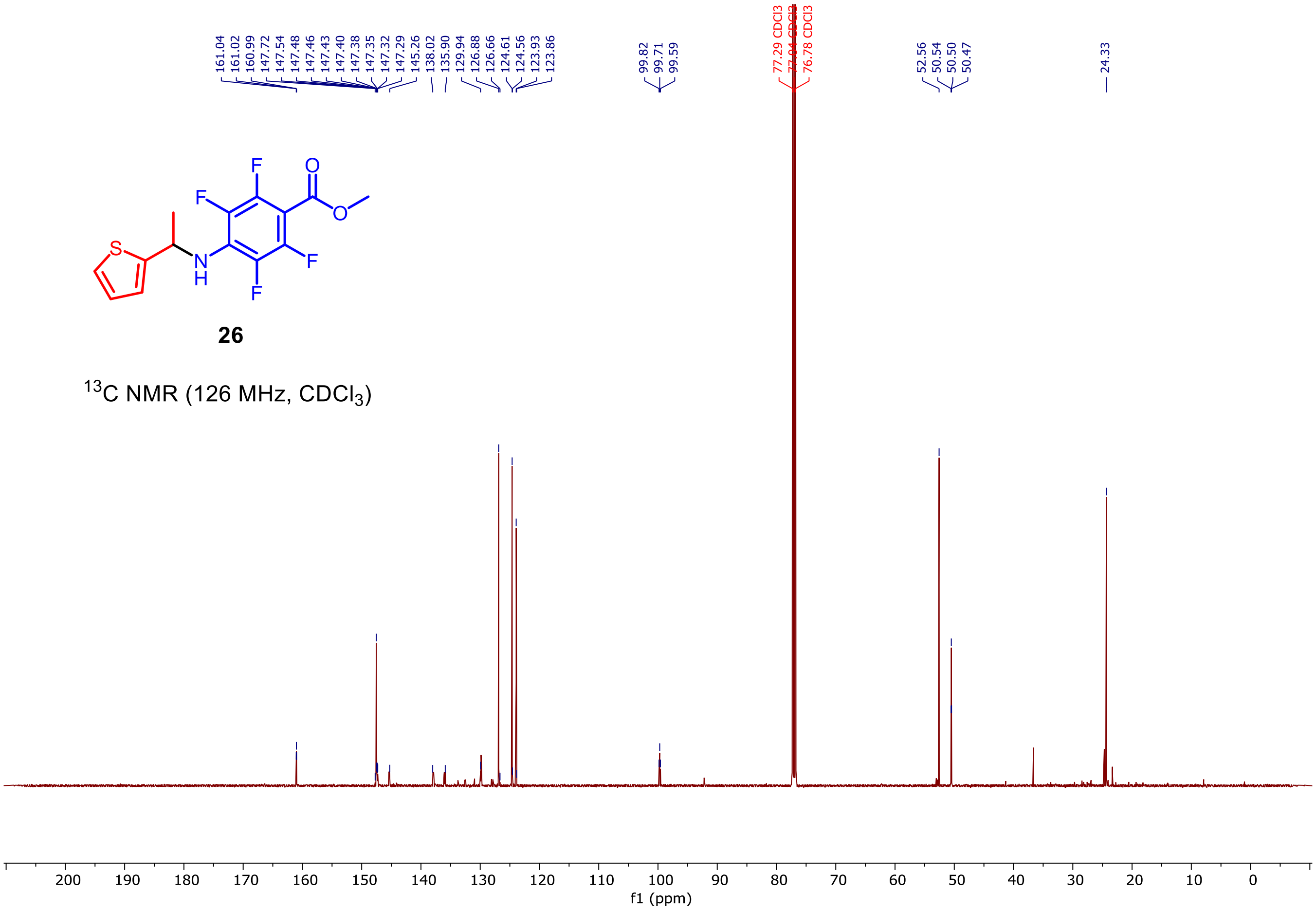

161.04
161.02
160.99
147.72
147.54
147.48
147.46
147.43
147.40
147.38
147.35
147.32
147.29
145.26
138.02
135.90
129.94
126.88
126.66
124.61
124.56
123.93
123.86
99.82
99.71
99.59
77.29 CDCl3
76.78 CDCl3
52.56
50.54
50.50
50.47
24.33
F
F
F
F
O
O
S
N
H
26
^{13}C NMR (126 MHz, CDCl$_3$)
200
190
180
170
160
150
140
130
120
110
100
90
80
70
60
50
40
30
20
10
0
f1 (ppm)

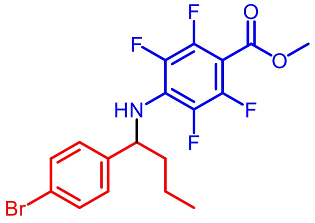

27

^{1}H NMR (500 MHz, $CDCl_3$)

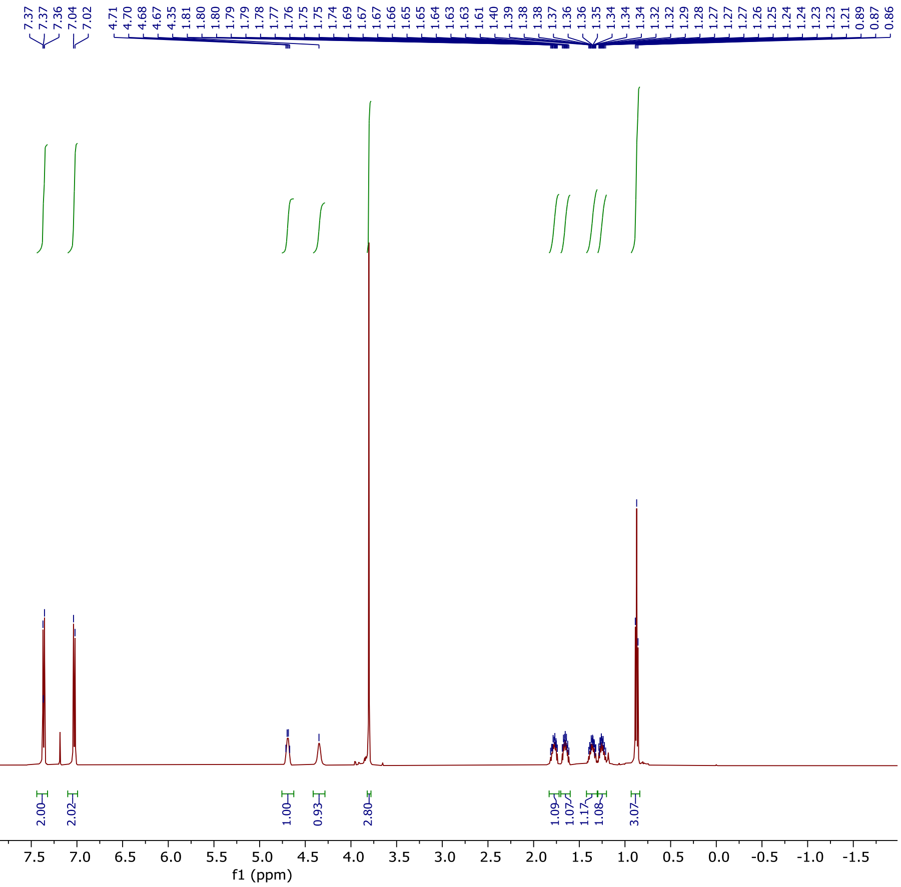

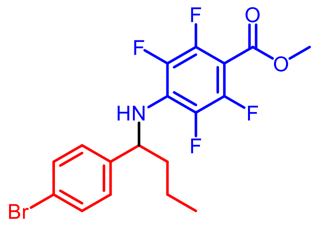

27

^{19}F NMR (471 MHz, $CDCl_3$)

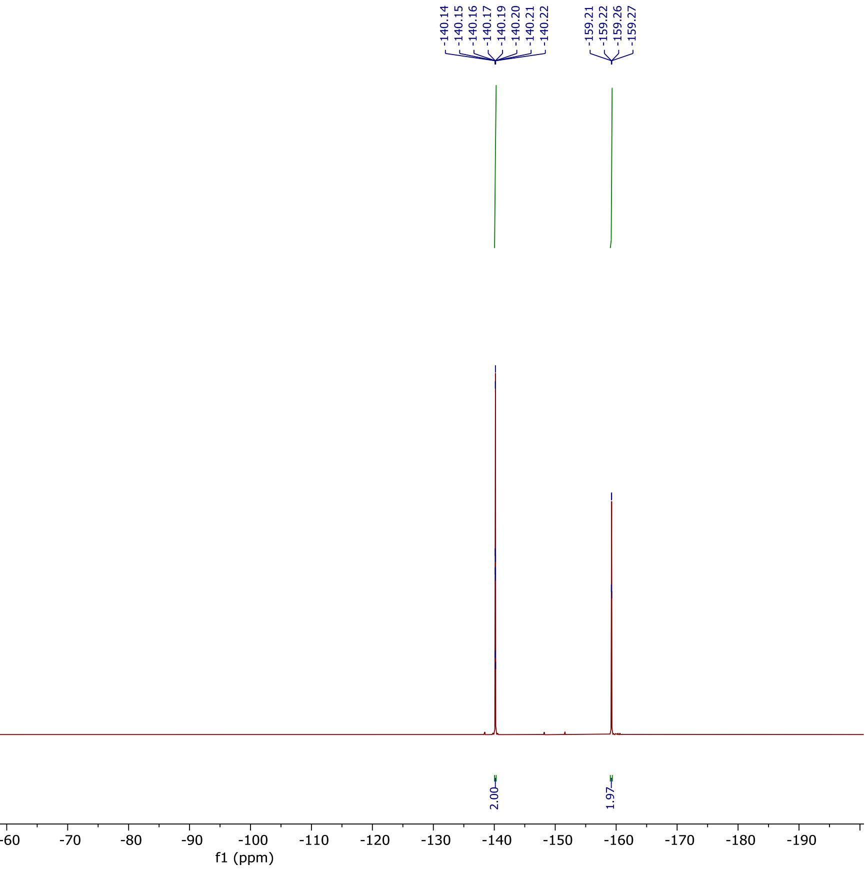

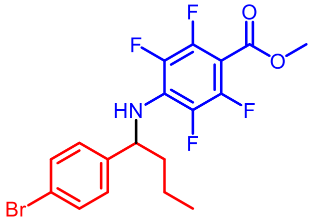

27

^{13}C NMR (126 MHz, $CDCl_3$)

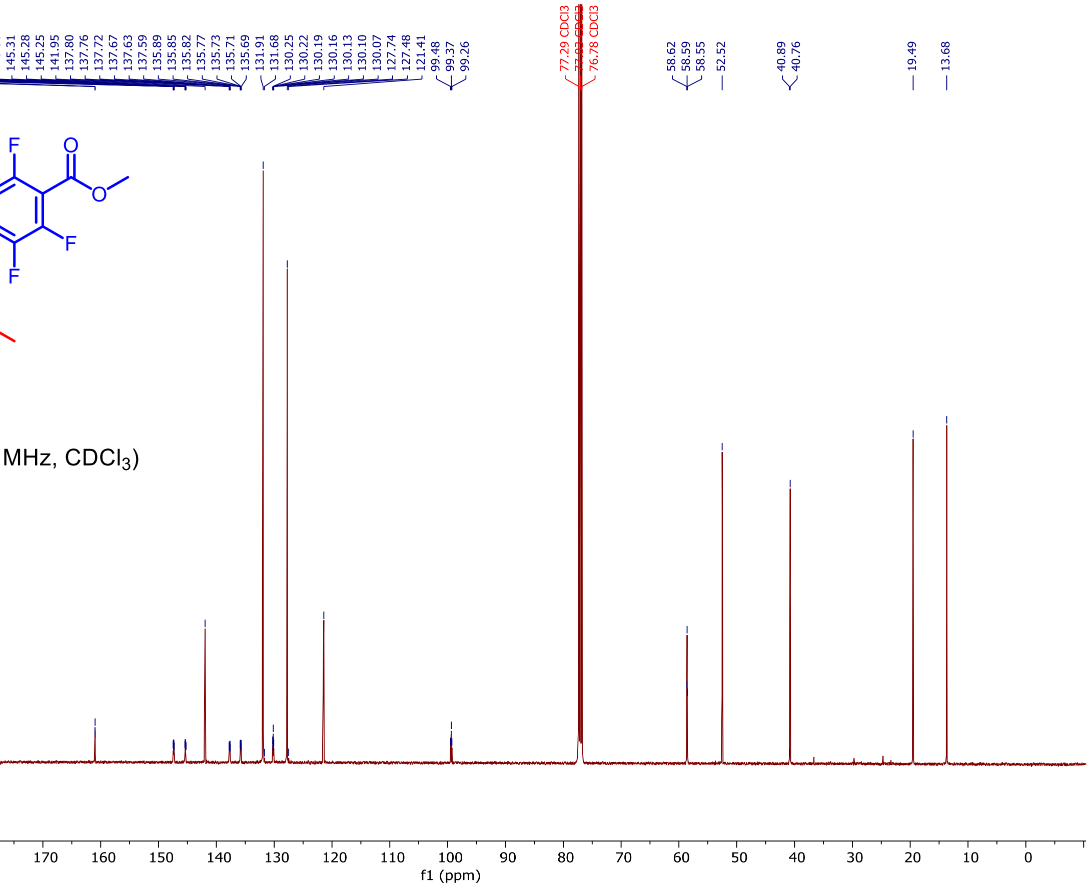

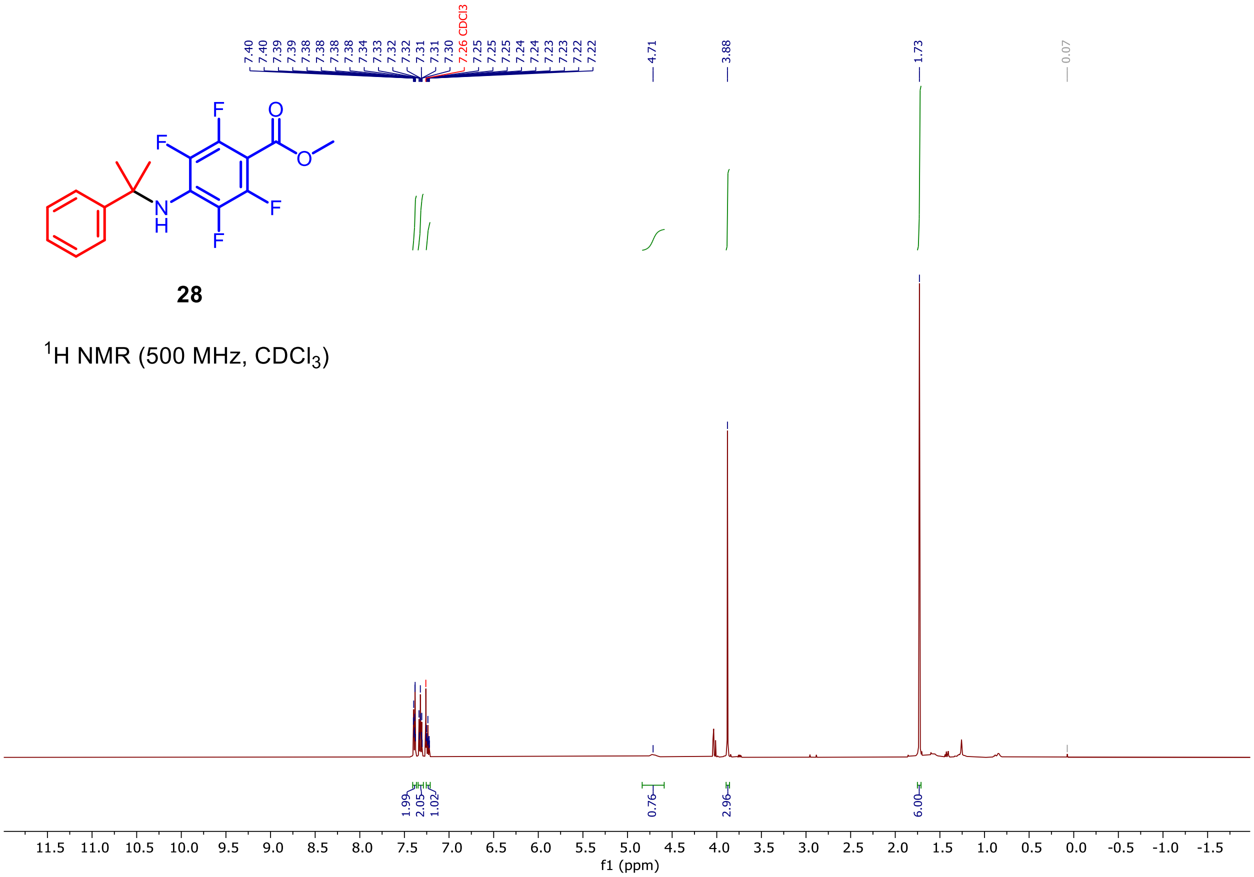

28

^{1}H NMR (500 MHz, $CDCl_3$)

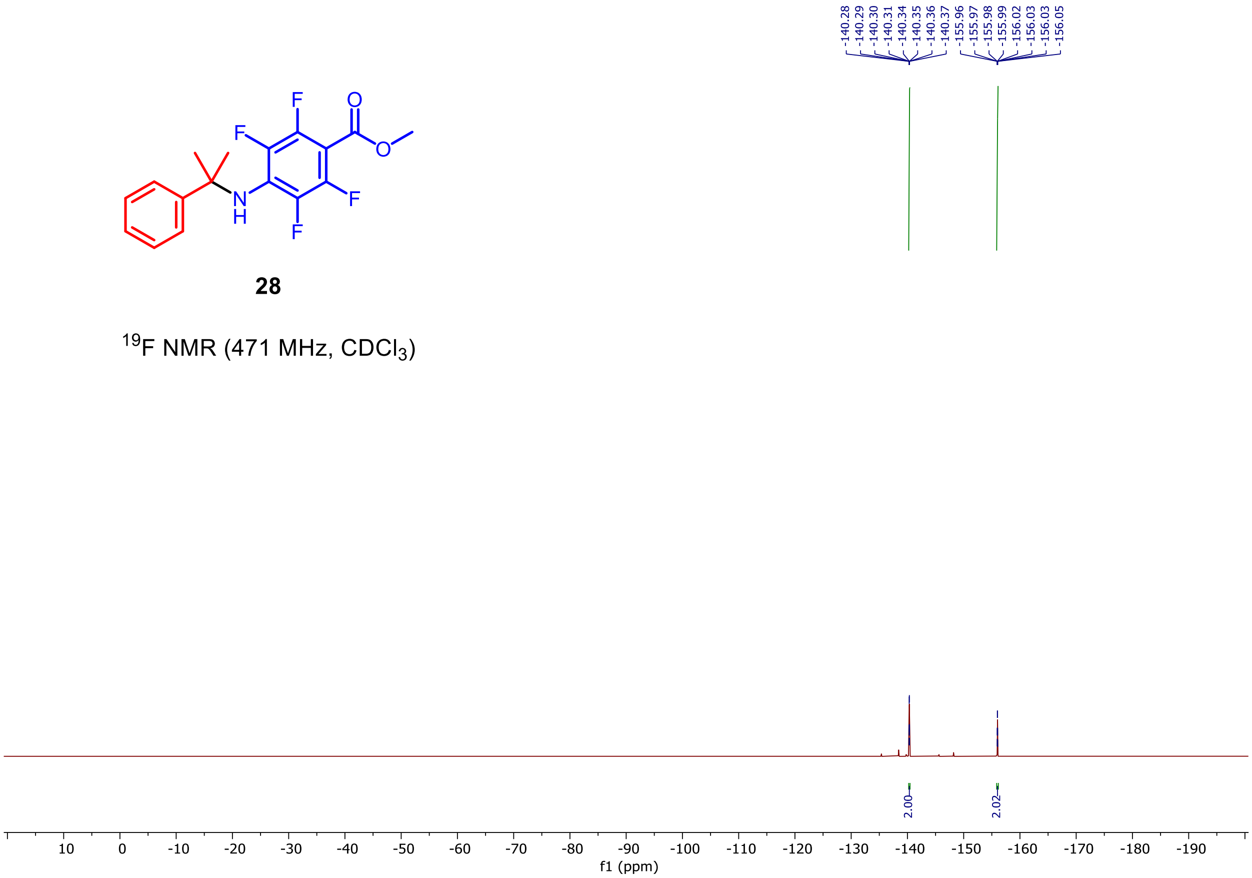

28

^{19}F NMR (471 MHz, $CDCl_3$)

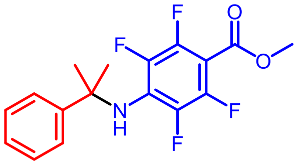

28

^{13}C NMR (126 MHz, $CDCl_3$)

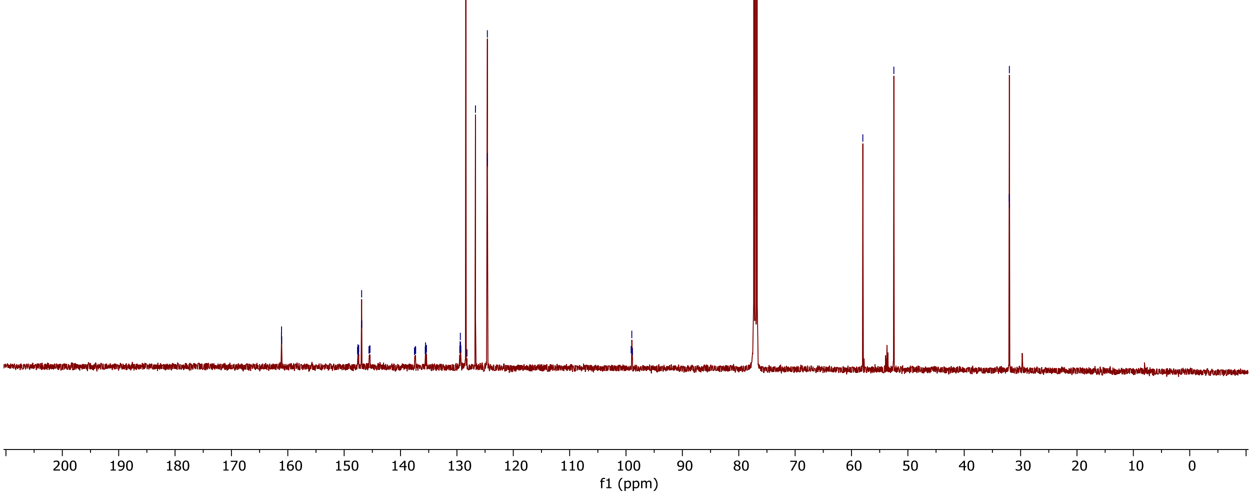

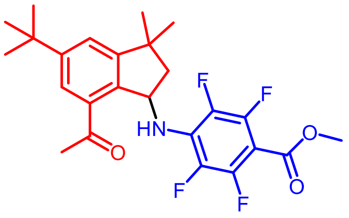

29

^1H NMR (500 MHz, $CDCl_3$)

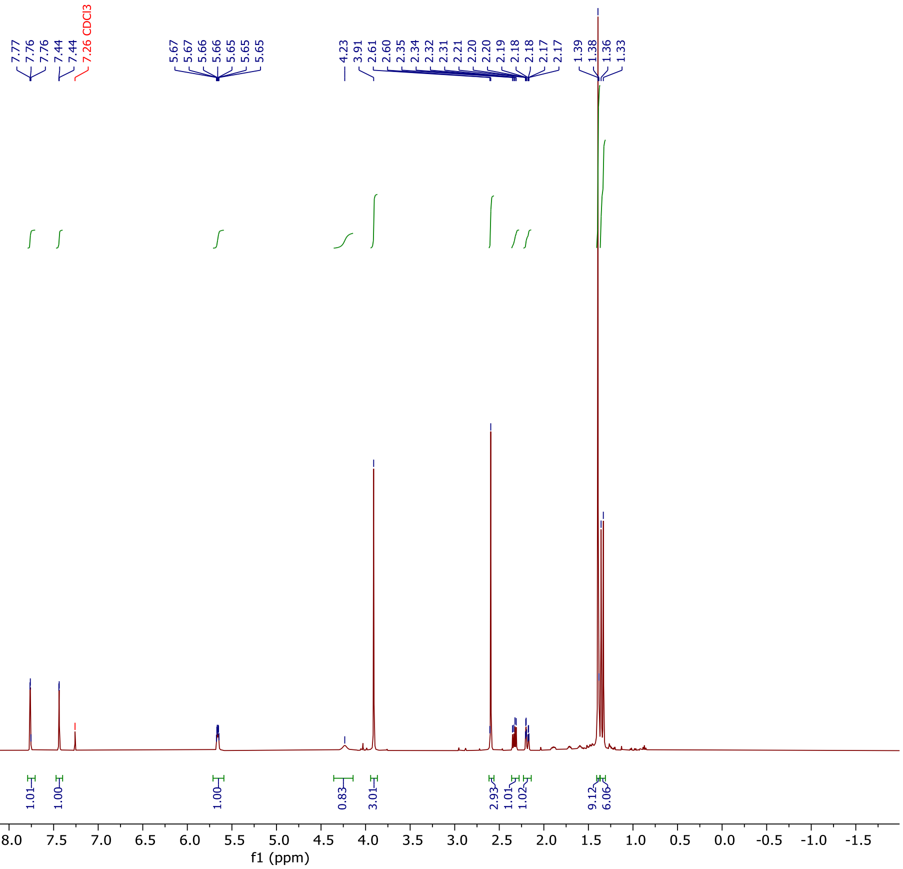

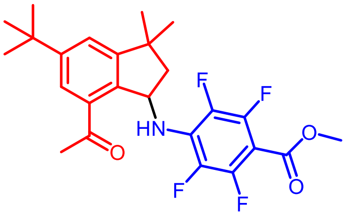

29

^{19}F NMR (471 MHz, $CDCl_3$)

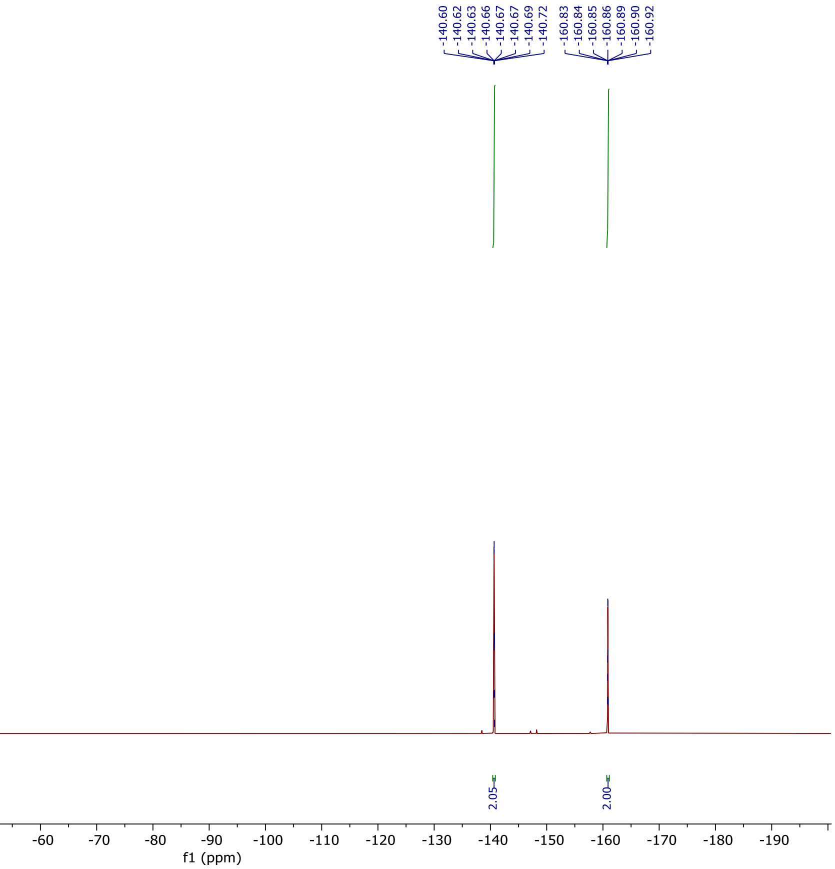

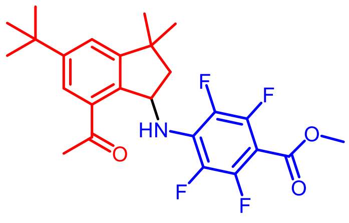

29

^{13}C NMR (126 MHz, $CDCl_3$)

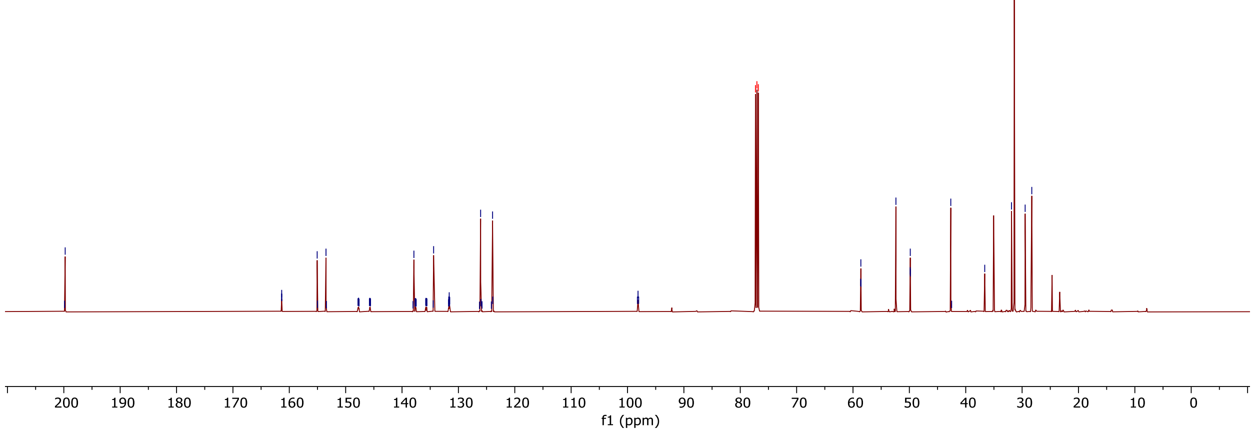

Supplement: CC-062-D6CC02735K-s001 [file CC-062-D6CC02735K-s001.pdf]
